# Infodemiology and Infoveillance: Scoping Review

**DOI:** 10.2196/16206

**Published:** 2020-04-28

**Authors:** Amaryllis Mavragani

**Affiliations:** 1 Department of Computing Science and Mathematics Faculty of Natural Sciences University of Stirling Stirling United Kingdom

**Keywords:** big data, infodemiology, infoveillance, internet, review, web-based data

## Abstract

**Background:**

Web-based sources are increasingly employed in the analysis, detection, and forecasting of diseases and epidemics, and in predicting human behavior toward several health topics. This use of the internet has come to be known as infodemiology, a concept introduced by Gunther Eysenbach. Infodemiology and infoveillance studies use web-based data and have become an integral part of health informatics research over the past decade.

**Objective:**

The aim of this paper is to provide a scoping review of the state-of-the-art in infodemiology along with the background and history of the concept, to identify sources and health categories and topics, to elaborate on the validity of the employed methods, and to discuss the gaps identified in current research.

**Methods:**

The PRISMA (Preferred Reporting Items for Systematic Reviews and Meta-Analyses) guidelines were followed to extract the publications that fall under the umbrella of infodemiology and infoveillance from the JMIR, PubMed, and Scopus databases. A total of 338 documents were extracted for assessment.

**Results:**

Of the 338 studies, the vast majority (n=282, 83.4%) were published with JMIR Publications. The Journal of Medical Internet Research features almost half of the publications (n=168, 49.7%), and JMIR Public Health and Surveillance has more than one-fifth of the examined studies (n=74, 21.9%). The interest in the subject has been increasing every year, with 2018 featuring more than one-fourth of the total publications (n=89, 26.3%), and the publications in 2017 and 2018 combined accounted for more than half (n=171, 50.6%) of the total number of publications in the last decade. The most popular source was Twitter with 45.0% (n=152), followed by Google with 24.6% (n=83), websites and platforms with 13.9% (n=47), blogs and forums with 10.1% (n=34), Facebook with 8.9% (n=30), and other search engines with 5.6% (n=19). As for the subjects examined, conditions and diseases with 17.2% (n=58) and epidemics and outbreaks with 15.7% (n=53) were the most popular categories identified in this review, followed by health care (n=39, 11.5%), drugs (n=40, 10.4%), and smoking and alcohol (n=29, 8.6%).

**Conclusions:**

The field of infodemiology is becoming increasingly popular, employing innovative methods and approaches for health assessment. The use of web-based sources, which provide us with information that would not be accessible otherwise and tackles the issues arising from the time-consuming traditional methods, shows that infodemiology plays an important role in health informatics research.

## Introduction

Infodemiology (ie, information epidemiology) is a field in health informatics defined as “*the science of distribution and determinants of information in an electronic medium, specifically the Internet, or in a population, with the ultimate aim to inform public health and public policy*” [1]. The first official mention of infodemiology according to a search in PubMed (ie, baring the term on the title) was by Gunther Eysenbach in 2002 [2]. However, infodemiology studies (ie, assessment of health-related topics using web-based data [3]) can be traced all the way back to 1996. Two more studies in the 2000s use the term (PubMed): one in 2004, where the quality of hospitals’ websites was assessed [4], and one in 2006, showing that flu data from Google correlated with influenza cases [5].

The large corpus of publications in infodemiology were present after 2009, with the first complete presentation and assessment of the subject being found in the scoping review of Bernardo et al [6] published with JMIR Publications—the main publisher of infodemiology and infoveillance studies.

Social media and search queries are the most popular sources for retrieving information from web-based sources. The use of social media is constantly expanding [7], with more users and including more features. Search query data is also of significant value, as they take into account the revealed and not the stated users’ preferences [8,9], but methodology should be designed with caution to ensure the validity of the results [10].

Popular social media data sources in infodemiology include Twitter [11-17], Facebook [18-22], and Instagram [23,24]. Queries from search engines are mostly retrieved by Google Trends [25-32], as well as Yandex [33-35], Baidu [36,37], Bing [38], Yahoo [39], and Daum [40,41]. Other popular sources include websites and platforms [42-45]; blogs, forums, and online communities [46-52]; and, what has received attention lately, mobile apps of certain health categories (eg, asthma [53] and heart failure self-care management [54]). Significant focus has been shown in combining two or more sources such as Facebook and Instagram [55], Facebook and Twitter Posts [56], US newspaper media and Facebook [57], and Google and Wikipedia [58].

The use of web-based sources offers an assessment of real-time information, whether it is from Twitter, Google, or other social media and search queries. For health data retrieved through traditional methods such as registries, questionnaires, or surveys, analysis and assessment can take time to perform. Thus, nowcasting using said methods is not trivial. However, web-based (real time) data has been shown to significantly contribute to the analysis and forecasting of certain diseases, outbreaks, and epidemics.

Therefore, this specific part of health informatics has benefitted from infodemiology. In particular, one of the most studied diseases is influenza, and several data sources have been employed to predict and assess flu-related topics [39,40,59-76]. Epidemics and infectious diseases that have been analyzed and assessed using infodemiology and infoveillance approaches include HIV/AIDS [77-79], measles [80-83], and the Zika virus [84-87].

Infodemiology topics have also been the subject of research for several reviews on various topics like curable sexually transmitted diseases (STDs) [88] and mental health disorders [89], and for data sources like search queries, social media [6], mobile phone apps [90], Twitter [91], and Google Trends [92].

Infodemiology has contributed to health assessment with the analysis of a range of topics. In specific, popular categories in the field of infodemiology and infoveillance include drugs [39,93,94], marijuana [95-97], depression and suicide [98-108], smoking and tobacco [109-116], electronic cigarettes (e-cigarettes) [117-126], and hookahs [127-130]. As far as chronic diseases are concerned, the assessment of diabetes [131-136] and multiple sclerosis [137,138] has benefitted from infodemiology and infoveillance studies. Other topics include breast cancer [139-142]; fitness and diet [143-146]; health care performance, evaluation, and dissemination [147,148]; and human papillomavirus (HPV) [149-154].

This review aims to update and expand the 2013 scoping review of Bernardo et al [6]. The authors of said review provided a well-structured outline of how infodemiology was employed in health informatics research up to that point, but as is evident, the large corpus of studies in the field have been published within the last couple of years. This update on the subject is important in identifying how infodemiology has contributed to health informatics over the past decade compared with traditional surveillance methods, the main web sources used, and the individual health categories and topics that have been explored. Apart from identifying the “metrics” of infodemiology studies (ie, numer of publications, thematic topics, journals and publishers, and data sources), this review aims to identify the advantages, disadvantages, and value and validity of infodemiology and infoveillance.

## Methods

**Figure 1:**
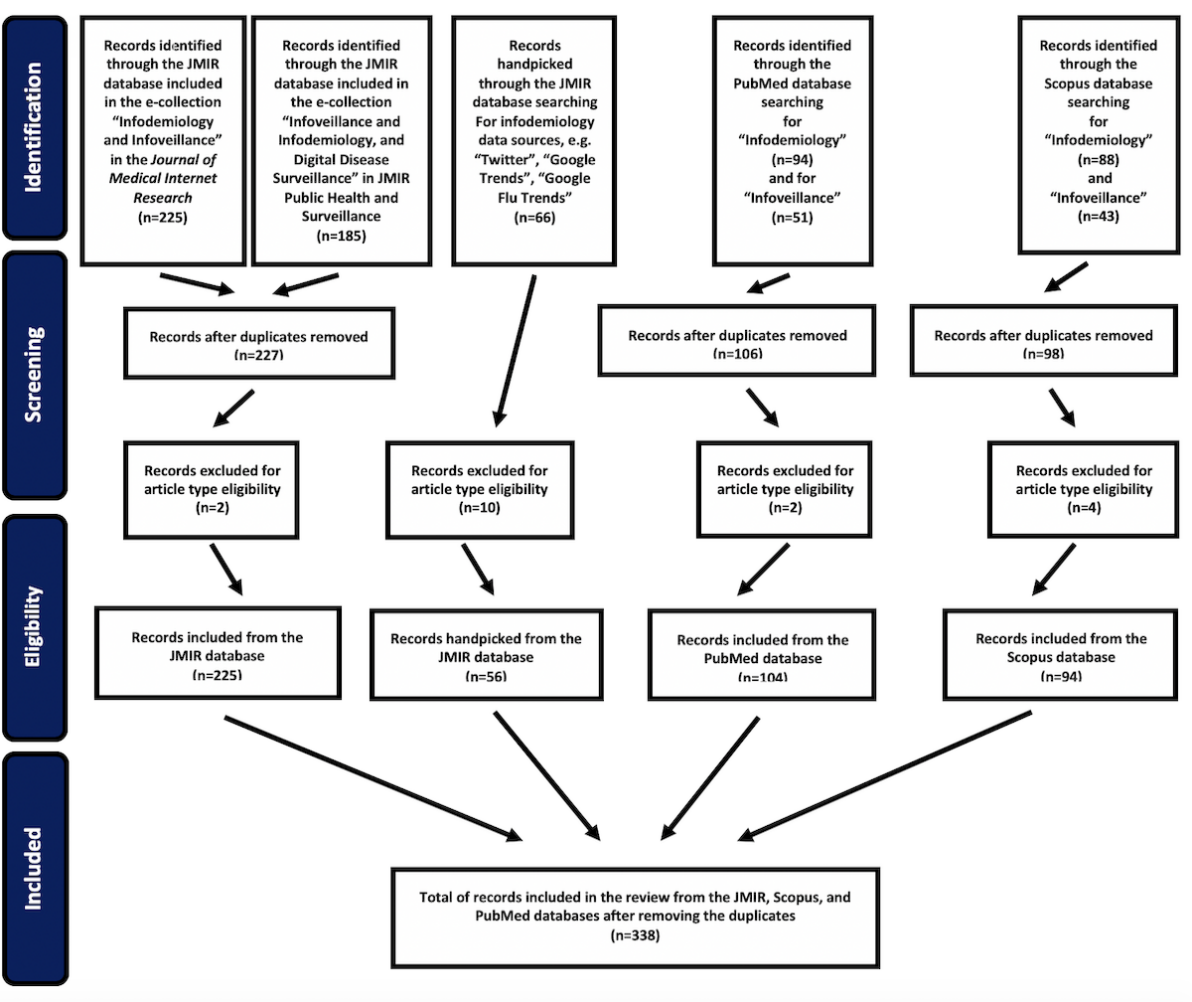
Preferred Reporting Items for Systematic Reviews and Meta-Analyses diagram for selecting the publications from JMIR, PubMed, and Scopus.

To select the publications in the fields of infodemiology and infoveillance, the Preferred Reporting Items for Systematic Reviews and Meta-Analyses (PRISMA) Extension for Scoping Reviews [155,156] were followed. The procedure is depicted in the PRISMA flow diagram in Figure 1.

In the JMIR Publications database, all papers from the two relevant electronic collections (e-collections) were retrieved: 225 documents from the “Infodemiology and Infoveillance” [157] e-collection in the *Journal of Medical Internet Research* and 185 from the “Infodemiology, Infoveillance, and Digital Disease Surveillance” [158] e-collection in *JMIR Public Health and Surveillance*. After removing the duplicates and 2 documents for article type eligibility, 227 documents were extracted in total. Next, 66 documents were handpicked from the JMIR publications database based on searches of data sources (ie, “Twitter,” “Google Trends,” and “Google Flu Trends”). After 10 documents were excluded based on article type, a total of 56 documents were handpicked from the JMIR database.

Next, the term “infodemiology” was searched for in the PubMed database from January 1, 2009, to December 31, 2018, in the search field “Title-Abstract.” The search returned 94 documents. The term “infoveillance” was then entered for the same period and in the same field, and the search returned 51 documents. The duplicates were 39 in total, and 2 documents were excluded based on article type; a total of 104 documents were extracted from the PubMed database. The terms “infodemiology” and “infoveillance” were then independently searched for in the Scopus database in the “Article title, Abstract, Keywords” field for the full years 2009-2018. The search returned 88 and 43 documents, respectively (ie, a total of 131). After removing 33 duplicates and 4 documents for article type criteria, a total of 94 documents were extracted from the Scopus database.

Overall, all studies that included the terms “infodemiology” or “infoveillance” in the “Title/Abstract” field in PubMed up to December 2018 and all studies including the terms “infodemiology” or “infoveillance” in the “Article title, Abstract, Keywords” field up to 2018 in Scopus were selected. For JMIR, all articles in the two relevant e-collections, as well as the articles derived by the individual data source searches, were included in this review. Articles were only excluded based on article type eligibility (eg, correction, erratum). After removing the duplicates from the JMIR, PubMed, and Scopus databases, the total extracted documents from all databases were 338.

## Results

**Figure 2:**
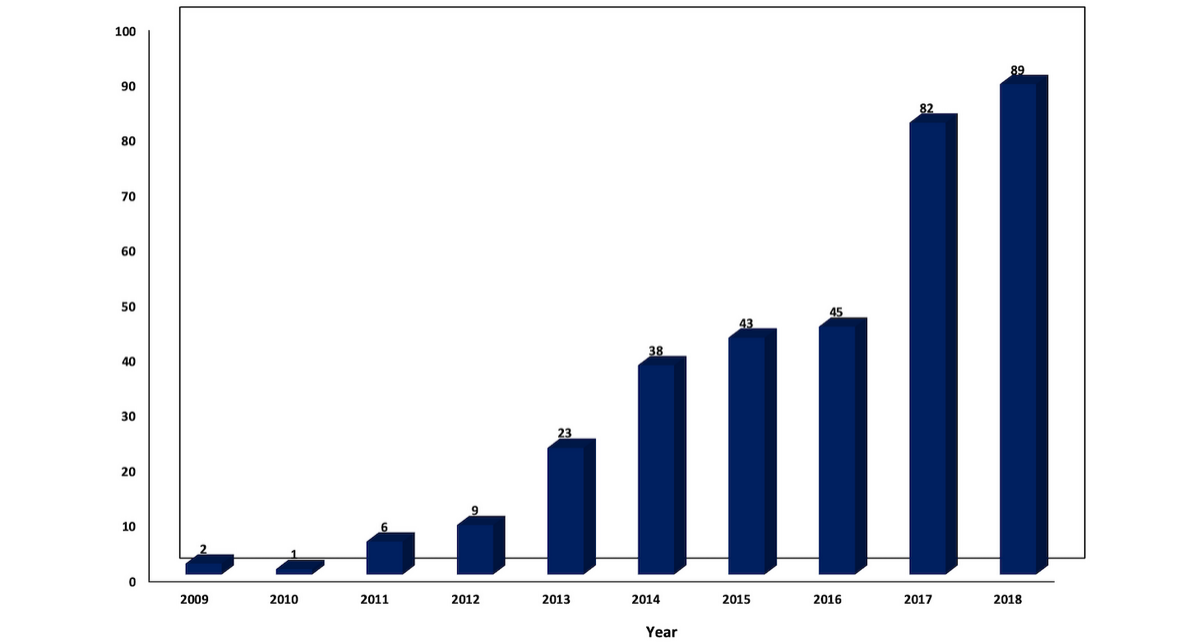
Number of publications in infodemiology and infoveillance by year (2009-2018).

Table A1 in Multimedia Appendix 1 consists of the 338 selected publications included in this review, and shows the authors’ names, publication year, the title, and the journal used for the analysis. Figure 2 depicts the number of publications by year from 2009 to 2018.

The number of publications in the subject is increasing every year, with 2018 featuring more than one-fourth of the 338 total publications (n=89, 26.3%), and the publications from 2017 and 2018 combined accounted for more than half (n=171, 50.6%) of the total number of publications in the past decade.

The 338 extracted studies were published in 57 journals in total. The vast majority of the studies in infodemiology and infoveillance were published with JMIR Publications (n=282, 83.4%). Specifically, the *Journal of Medical Internet Research* features almost half of the publications (n=168, 49.7%), and *JMIR Public Health and Surveillance* features almost one-fourth of the examined studies (n=74, 21.9%).

**Figure 3:**
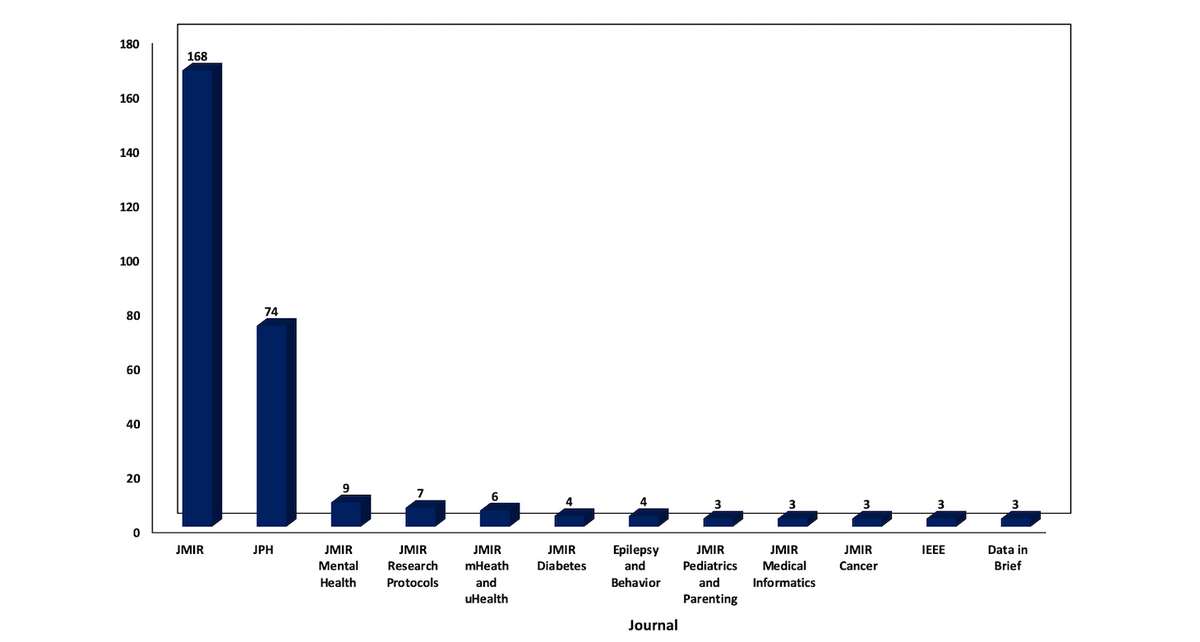
Number of publications in infodemiology and infoveillance by journal (2009-2018). IEEE: Institute of Electrical and Electronics Engineers; JPH: JMIR Public Health and Surveillance.

Figure 3 consists of the numbers of publications per journal with >2 publications on the subject.

Journals that have published more than 1 paper in the subject included JMIR Mental Health (n=9), JMIR Research Protocols (n=7), JMIR Diabetes (n=4), JMIR Cancer (n=3), JMIR Medical Informatics (n=3), JMIR Pediatrics and Parenting (n=3), the Journal of Big Data (n=2), and the Interactive Journal of Medical Research (n=2).

**Figure 4:**
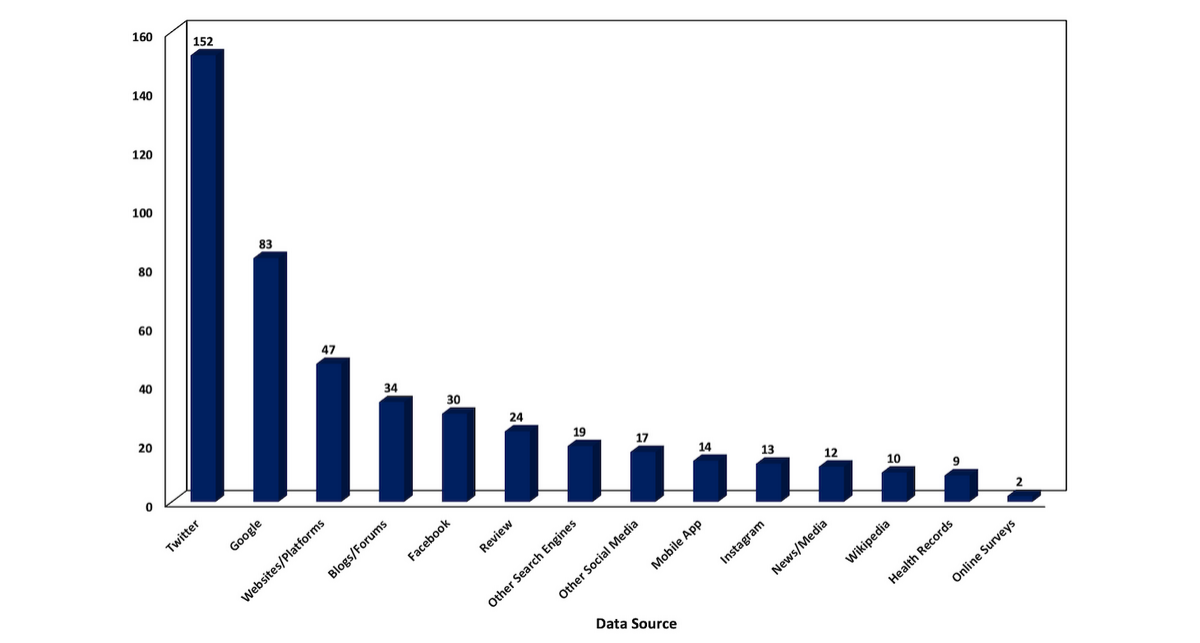
Number of publications by data source (2009-2018).

Table A2 of Multimedia Appendix 1 consists of the 338 publications categorized by data source employed. Figure 4 depicts the popularity of the examined data sources in terms of number of publications (some publications employed more than one data source).

Of the 338 publications, the most popular source was Twitter with 45.0% (n=152) and is continuously gaining popularity. Google sources were in second place with 24.6% (n=83), followed by websites and platforms with 13.9% (n=47), blogs and forums with 10.1% (n=34), Facebook with 8.9% (n=30), and other search engines with 5.6% (n=19). The Google category consisted mainly of publications using Google Trends; although, the following Google tools have also been identified as main data sources in several publications: Google Flu Trends (n=6), Google Analytics (n=2), Google Insights (n=2), Google Correlate (n=1), Google Health (n=1), Google News (n=1), Google AdWords (n=1), Google Video (n=1), and Google Blog Search (n=1).

The “other search engines” category consists of Bing (n=7), Baidu (n=4), Yandex (n=4), Daum (n=2), and Yahoo (n=3), and the “other social media” category consists of YouTube (n=5), Yelp (n=5), Google+ (n=4), Foursquare (n=1), SoundCloud (n=1), Tumblr (n=1), Pinterest (n=1), and MySpace (n=1). Yahoo answers (n=2) was included in the blogs, forums, and communities category.

**Figure 5:**
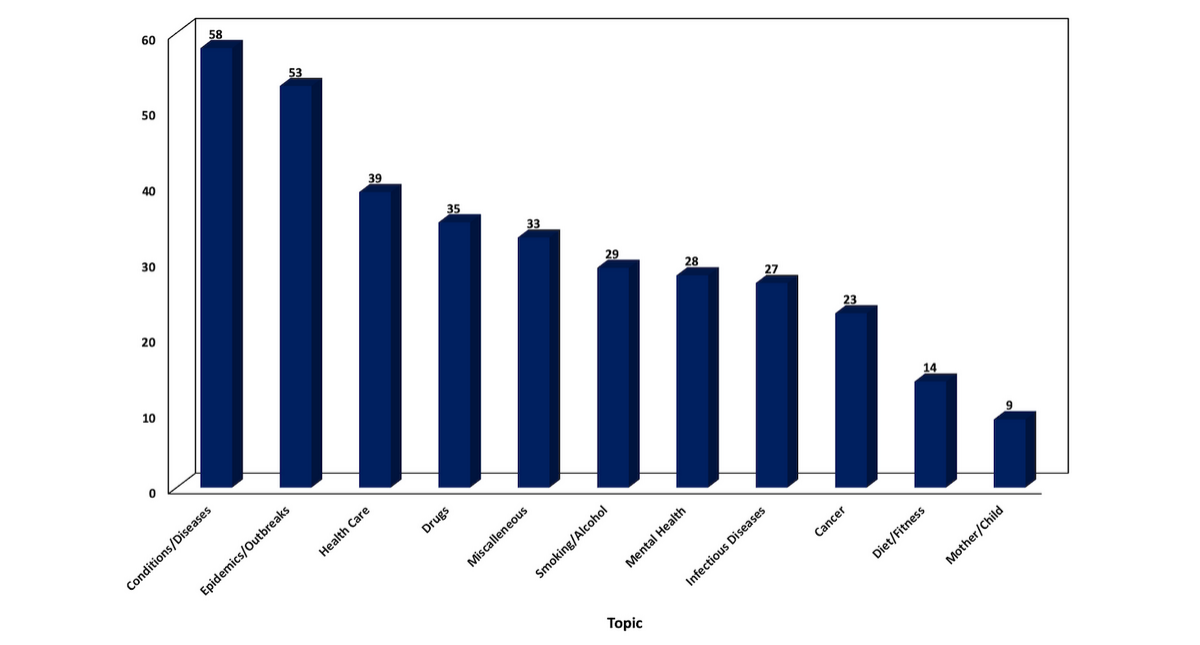
Health categories by number of publications (2009-2018).

Although many health topics have been examined in infodemiology and infoveillance, some are significantly more popular. Figure 5 depicts the general categories in terms of number of publications, while Figure A1 of Multimedia Appendix 2 consists of the pie charts of their subcategories. All individual topics and subtopics identified in this review by number of publications can be found in Table A1 of Multimedia Appendix 2.

In the 338 publications examined in this review, the most popular subjects were conditions and diseases with 17.2% (n=58) and epidemics and outbreaks with 15.7% (n=53), followed by health care with 11.5% (n=39), drugs with 10.4% (n=35), smoking and alcohol with 8.6% (n=29), and mental health with 8.3% (n=28). Infectious diseases with 8.0% (n=27) and cancer with 6.8% (n=23) were also featured in several publications. The categories of diet and fitness with 4.1% (n=14) and mother and child with 2.7% (9) were less popular.

## Discussion

### Principal Findings

In this scoping review, the most popular web-based data source as well as the most studied health categories in infodemiology and infoveillance were identified through a systematic search of the existing literature.

#### Time Line and Journals

Based on the results, the use of web-based data in health informatics is significantly increasing year by year, with half of the publications from 2009 to 2018 being in the last couple of years. The JMIR Publications database is the most significant contributor in terms of the number of publications on the subject (n=282/338, 83.4%), especially given that the most significant infodemiology-oriented journal (ie, JMIR Public Health and Surveillance) is published with JMIR Publications. The concept of infodemiology, introduced by Gunther Eysenbach in 2002, has been gaining significant recognition since its birth, and it is evident that it will play an even more significant role in health informatics in the years to come, especially as internet penetration increases along with the average age of the users.

#### Data Sources and Tools

**Figure 6:**
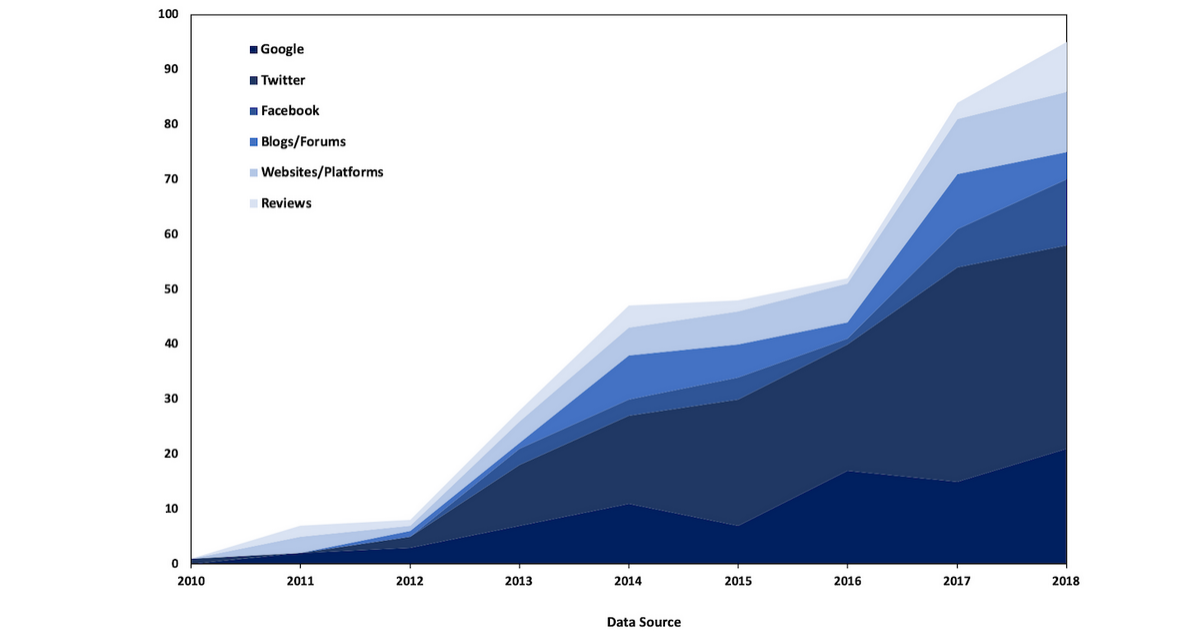
Yearly changes in number of publications for the most popular data sources (2009-2018).

Figure 6 depicts the yearly changes in number of publications for the most popular data sources over the examined period. As evident, there was a significant increase in the use of web-data sources over the last couple of years, with Twitter in the lead in assessing health-related topics by health informatics researchers.

Despite the increasingly large number of users and the fact that Twitter is used significantly more than Google, Twitter has the limitation of not being universal. Its pros include that it is an outlet for official reports and news (eg, governmental, politicians), but a significant con is that it is not used by all; furthermore, not everyone interacts on the site (ie, tweets or retweets). The analysis of internet search traffic data—mainly from Google but from other search engines as well (eg, Bing, Yahoo)—is more universal in the sense that internet penetration has increased to a point where the large majority of people have access to and use the internet and searching for keywords in search engines is the most used internet feature. Apart from this, it also ensures anonymity, deeming it more reliable, as it uses the revealed and not the stated users’ preferences. However, the choosing of the keywords (queries) as well as the methodology for selecting the retrieved data is much more complicated than with Twitter. In addition, more than one search engine exists, and thus, not all queries (data) on the respective selected topic can be retrieved.

On the other hand, there is a significant rise in the percentage of publications in the last couple of years using data from other social media such as Facebook and Instagram. This could be showing that younger internet users’ preferences in the use of social media may be revealing a trend of said platforms over original search queries and websites. Researchers in this field should closely follow any potential shifts in internet use, along with the correspondence and age of users, to ensure—to the point that is possible with web-based data—that the sample is representative and the research aims to change along with what is trending. The latter is crucial for infodemiology research to continue to be valid, and it should be given significant focus.

#### Health Topics

As is evident, there is a wide variety of topics that have been studied up to this point. The most popular topics identified in this review were illegal drugs, breast cancer, smoking, fitness, HIV/AIDS, depression, diabetes, influenza, HPV, multiple sclerosis, Zika virus, suicide, STDs, and e-cigarettes, and significant attention has been given to the evaluation of health care such as hospital ratings and patients’ experiences and health topics’ and medical institutions’ dissemination strategies.

Approaches include nowcasting epidemics and outbreaks, surveillance of infectious diseases, assessment of chronic conditions, and basically everything traditional surveillance methods have aimed to do. Thus, the results of this review show not only the increasing popularity of web-based data but also their significant contribution to the existing literature, as well as the value of infodemiology in health informatics.

#### Advantages and Disadvantages

The difference in using web-based data—infodemiology’s main advantage—is that it tackles the issue of traditional surveillance methods not providing real time assessments. Even in the health sector, where data are generally available compared to other topics, the gathering, assessing, and publishing of health data can sometimes take years to process. This is less of an issue for topics such as chronic diseases that are not infectious, but it makes the assessment and forecasting of epidemics and outbreaks much more complicated.

Another significant advantage of infoveillance compared to traditional surveillance methods is the anonymity that web-based data offers. Online search traffic data are completely anonymous, and in most social media and forums, an individual has the option of anonymity. In this way, data retrieved from said sources are the revealed and not the stated preferences, which can be a plus for sensitive topics such as AIDS or STDs.

Despite the many advantages that web-based data sources have to offer, several limitations have been identified in the use of infodemiology sources. The main disadvantage of using web-based sources is that the data can be affected by sudden incidents or events, which, especially in nowcasting or when the number of observations is low, could provide biased results. Similarly, the sample cannot be shown to be representative, especially in the assessment of online search traffic data; although, this is less of an issue as internet penetration increases.

With real time data that can be retrieved from web sources, disease surveillance has become much faster than with traditional methods, and web sources also have the benefit of assessing large populations, which contrasts with most traditional methods that are based on data retrieved from significantly smaller groups, such as with interviews or questionnaires. Overall, what health informatics should aim toward in the future is to combine web-data sources with traditional data assessment to provide an even more complete assessment.

### Limitations

The main limitation of this scoping review was that not all infodemiology papers could be included. Though the selection of publications for this work was thorough and followed the guidelines for proper selection and included the main outlets for infodemiology papers (ie, JMIR Publications and PubMed), some publications may have been left out; a limitation that all reviews have. Specifically, articles using the two most popular infodemiology sources (ie, Twitter and Google) were only searched for in the JMIR database. Studies using, for example, Google Trends and Twitter constitute a large body of the relevant literature, and a significant number of said studies were not included as they did not use the specific searched for terms (ie, infodemiology and infoveillance); the latter being the main difference from the original Bernardo et al [6] scoping review. However, despite the possible reduced number of included publications that use infodemiology and infoveillance sources but not the infodemiology or infoveillance terms, as JMIR is the main outlet for such themed publications, this scoping review gives a valuable qualitative and quantitative overview of how the concept has progressed over the last decade, as well as identifying the main sources and topics that have been used and assessed. Future work should focus on expanding the present results, as well as recording infodemiology papers based on tools used. As the search by source yields many results, focus should be given to future systematic reviews on the subject by source as has been done, for example, for the use of Google Trends [92].

### Conclusions

Using web-based sources in epidemiology and disease surveillance has shown to be valuable and valid over the past decade, and the results of this scoping review clearly point to this direction. Data sources cover a wide variety of tools, social media, platforms, websites, blogs, and search engines, and the topics that are the most studied vary from chronic disease prevalence to nowcasting epidemics. Infodemiology and infoveillance tackle several of the issues that arise with traditional assessment methods, and, as internet penetration increases, employing web data sources for health assessment could be the future in health informatics.

## Appendix

Multimedia Appendix 1 Infodemiology and infoveillance publications from JMIR, Scopus, and PubMed (2009-2018).

Multimedia Appendix 2 Topics and subtopics (#publications) in infodemiology and infoveillance (2009- 2018).

## Abbreviations

e-cigarettes: electronic cigarettes
e-collections: electronic collections
HPV: human papillomavirus
PRISMA: Preferred Reporting Items for Systematic Reviews and Meta-Analyses
STD: sexually transmitted disease
