# Supplementary material for: Infodemiology and Infoveillance: Scoping Review
Source: J Med Internet Res. 2020 Apr 28;22(4):e16206. doi: 10.2196/16206 (PMC7189791; doi:10.2196/16206)
Supplement: Multimedia Appendix 1 [file jmir_v22i4e16206_app1.pdf]

Multimedia Appendix 1:
Infodemiology and Infoveillance: A Scoping Review

Table A1 consists of the complete list of the 338 extracted publications from the JMIR, PubMed, and Scopus databases in the fields of Infodemiology and Infoveillance from 2009 to 2018.

Table A1. List of Infodemiology and Infoveillance Publications from JMIR, Scopus, and PubMed (2009-2018)

	Authors	Year	Title	Journal
1	Abbate et al	2017	Recruiting Women to a Mobile Health Smoking Cessation Trial: Low- and No-Cost Strategies	JMIR Research Protocols
2	Abbe & Falissard	2017	Stopping Antidepressants and Anxiolytics as Major Concerns Reported in Online Health Communities: A Text Mining Approach	JMIR Mental Health
3	Abdellaoui et al.	2017	Filtering Entities to Optimize Identification of Adverse Drug Reaction From Social Media: How Can the Number of Words Between Entities in the Messages Help?	JMIR Public Health and Surveillance
4	Abdellaoui et al.	2018	Detection of Cases of Noncompliance to Drug Treatment in Patient Forum Posts: Topic Model Approach.	Journal of Medical Internet Research
5	Adams	2013	POST-PANOPTIC SURVEILLANCE THROUGH HEALTHCARE RATING SITES: Who's watching whom?	Information Communication and Society
6	Adawi et al.	2017	Discrepancies Between Classic and Digital Epidemiology in Searching for the Mayaro Virus: Preliminary Qualitative and Quantitative Analysis of Google Trends	JMIR Public Health and Surveillance
7	Adrover et al.	2015	Identifying Adverse Effects of HIV Drug Treatment and Associated Sentiments Using Twitter	JMIR Public Health and Surveillance
8	Adusumalli et al.	2015	Assessment of Web-Based Consumer Reviews as a Resource for Drug Performance	Journal of Medical Internet Research
9	Agarwal et al.	2016	Impact of Predicting Health Care Utilization Via Web Search Behavior: A Data-Driven Analysis	Journal of Medical Internet Research
10	Albalawi & Sixsmith	2015	Agenda Setting for Health Promotion: Exploring an Adapted Model for the Social Media Era	JMIR Public Health and Surveillance
11	Allem et al.	2017	Images of Little Cigars and Cigarillos on Instagram Identified by the Hashtag #swisher: Thematic Analysis	Journal of Medical Internet Research
12	Allem et al.	2018	Hookah-Related Posts to Twitter From 2017 to 2018: Thematic Analysis.	Journal of Medical Internet Research
13	Allem et al.	2017	E-Cigarette Surveillance With Social Media Data: Social Bots, Emerging Topics, and Trends.	JMIR Public Health and Surveillance
14	Allem et al.	2017	Identifying Sentiment of Hookah-Related Posts on Twitter	JMIR Public Health and Surveillance
15	Alnemer et al	2015	Are Health-Related Tweets Evidence Based? Review and Analysis of Health-Related Tweets on Twitter	Journal of Medical Internet Research
16	Alvarez-Mon et al.	2018	Increasing Interest of Mass Communication Media and the General Public in the Distribution of Tweets About Mental Disorders: Observational Study	Journal of Medical Internet Research
17	Alvaro et al.	2017	TwiMed: Twitter and PubMed Comparable Corpus of Drugs, Diseases, Symptoms, and Their Relations	JMIR Public Health and Surveillance
18	Anderson et al.	2017	Using Social Listening Data to Monitor Misuse and Nonmedical Use of Bupropion: A Content Analysis.	JMIR Public Health and Surveillance
19	Aoki et al.	2018	Analysis of the Regionality of the Number of Tweets Related to the 2011 Fukushima Nuclear Power Station Disaster: Content Analysis	JMIR Public Health and Surveillance
20	Arnhold et al	2014	Mobile Applications for Diabetics: A Systematic Review and Expert-Based Usability Evaluation Considering the Special Requirements of Diabetes Patients Age 50 Years or Older	Journal of Medical Internet Research
21	Aslam et al.	2014	The reliability of tweets as a supplementary method of seasonal influenza surveillance.	Journal of Medical Internet Research
22	Athilingam & Jenkins	2018	Mobile Phone Apps to Support Heart Failure Self-Care Management: Integrative Review	JMIR Cardio
23	Ayers et al.	2012	A novel evaluation of World No Tobacco day in Latin America.	Journal of Medical Internet Research
24	Ayers et al.	2016	Leveraging Big Data to Improve Health Awareness Campaigns: A Novel Evaluation of the Great American Smokeout.	JMIR Public Health and Surveillance
25	Balls-Berry et al	2018	Linking Podcasts With Social Media to Promote Community Health and Medical Research: Feasibility Study	JMIR Formative Research
26	Baltrusaitis et al.	2017	Determinants of Participants' Follow-Up and Characterization of Representativeness in Flu Near You, A Participatory Disease Surveillance System	JMIR Public Health and Surveillance
27	Ben-Sasson & Yom-Tov	2016	Online Concerns of Parents Suspecting Autism Spectrum Disorder in Their Child: Content Analysis of Signs and Automated Prediction of Risk	Journal of Medical Internet Research
28	Berlinberg et al.	2018	Monitoring Interest in Herpes Zoster Vaccination: Analysis of Google Search Data.	JMIR Public Health and Surveillance
29	Bernardo et al.	2013	Scoping Review on Search Queries and Social Media for Disease Surveillance: A Chronology of Innovation	Journal of Medical Internet Research
30	Berry et al	2017	#WhyWeTweetMH: Understanding Why People Use Twitter to Discuss Mental Health Problems	Journal of Medical Internet Research
31	Bian et al.	2017	Using Social Media Data to Understand the Impact of Promotional Information on Laypeople's Discussions: A Case Study of Lynch Syndrome	Journal of Medical Internet Research
32	Birnbaum et al.	2017	A Collaborative Approach to Identifying Social Media Markers of Schizophrenia by Employing Machine Learning and Clinical Appraisals	Journal of Medical Internet Research
33	Bollegala et al.	2018	Causality Patterns for Detecting Adverse Drug Reactions From Social Media: Text Mining Approach	JMIR Public Health and Surveillance

	Authors	Year	Title	Journal
34	Bousquet et al.	2017	The Adverse Drug Reactions from Patient Reports in Social Media Project: Five Major Challenges to Overcome to Operationalize Analysis and Efficiently Support Pharmacovigilance Process	JMIR Research Protocols
35	Bragazzi	2013	A Google Trends-based approach for monitoring NSSI.	Psychology Research and Behavior Management
36	Bragazzi	2013	Infodemiology and infoveillance of multiple sclerosis in Italy.	Multiple Sclerosis International
37	Bragazzi et al.	2016	Infodemiology of status epilepticus: A systematic validation of the Google Trends-based search queries.	Epilepsy and Behavior
38	Bragazzi et al.	2016	Infodemiological data of West-Nile virus disease in Italy in the study period 2004-2015.	Data in Brief
39	Bragazzi et al.	2016	Infodemiological data concerning silicosis in the USA in the period 2004-2010 correlating with real-world statistical data.	Data in Brief
40	Braithwaite et al.	2016	Validating Machine Learning Algorithms for Twitter Data Against Established Measures of Suicidality	JMIR Mental Health
41	Brigo & Erro	2016	Why do people google movement disorders? An infodemiological study of information seeking behaviors.	Neurological Sciences
42	Brigo & Trinka	2015	Google search behavior for status epilepticus.	Epilepsy and Behavior
43	Brigo et al.	2015	Terminology of psychogenic nonepileptic seizures.	Epilepsia
44	Brigo et al.	2014	Why do people Google epilepsy? An infodemiological study of online behavior for epilepsy-related search terms.	Epilepsy and Behavior
45	Brigo et al.	2015	Wikipedia and neurological disorders.	Journal of Clinical Neuroscience
46	Brigo et al.	2018	Why do people search Wikipedia for information on multiple sclerosis?	Multiple Sclerosis and Related Disorders
47	Brigo et al.	2018	Italian Wikipedia and epilepsy: An infodemiological study of online information-seeking behavior.	Epilepsy and Behavior
48	Brigo et al.	2014	Web search behavior for multiple sclerosis: An infodemiological study.	Multiple Sclerosis and Related Disorders
49	Brigo et al.	2015	Information-seeking behaviour for epilepsy: an infodemiological study of searches for Wikipedia articles.	Epileptic Disorders
50	Brigo et al.	2016	Cancer information disparities on the internet: An infodemiological study	Journal of Cancer Policy
51	Broniatowski et al.	2015	Using Social Media to Perform Local Influenza Surveillance in an Inner-City Hospital: A Retrospective Observational Study	JMIR Public Health and Surveillance
52	Bubenzer	2009	Infodemiology as shown by influenza: New opportunities of the Internet [Infodemiologie am beispiel influenza: Die neuen chancen des Internets]	Klinikarzt
53	Burton et al.	2012	Right time, right place" health communication on Twitter: value and accuracy of location information.	Journal of Medical Internet Research
54	Callahan et al.	2015	Analyzing Information Seeking and Drug-Safety Alert Response by Health Care Professionals as New Methods for Surveillance	Journal of Medical Internet Research
55	Carrotte et al.	2017	“Fitspiration” on Social Media: A Content Analysis of Gendered Images	Journal of Medical Internet Research
56	Cartwright et al.	2018	Identifying National Availability of Abortion Care and Distance From Major US Cities: Systematic Online Search	Journal of Medical Internet Research
57	Cavazos-Regh et al.	2014	Characterizing the Followers and Tweets of a Marijuana-Focused Twitter Handle	Journal of Medical Internet Research
58	Cawkwell et al.	2015	Tracking Hookah Bars in New York: Utilizing Yelp as a Powerful Public Health Tool	JMIR Public Health and Surveillance
59	Chan et al.	2013	Infodemiology of alcohol use in Hong Kong mentioned on blogs: infoveillance study.	Journal of Medical Internet Research
60	Chen & Dredze	2018	Vaccine Images on Twitter: Analysis of What Images are Shared	Journal of Medical Internet Research
61	Chen et al. []	2018	Nature and Diffusion of Gynecologic Cancer–Related Misinformation on Social Media: Analysis of Tweets	Journal of Medical Internet Research
62	Chen et al.	2018	Does Eating Chicken Feet With Pickled Peppers Cause Avian Influenza? Observational Case Study on Chinese Social Media During the Avian Influenza A (H7N9) Outbreak.	JMIR Public Health and Surveillance
63	Chen et al.	2018	Dynamics of Health Agency Response and Public Engagement in Public Health Emergency: A Case Study of CDC Tweeting Patterns During the 2016 Zika Epidemic.	JMIR Public Health and Surveillance
64	Chen et al.	2015	What Online Communities Can Tell Us About Electronic Cigarettes and Hookah Use: A Study Using Text Mining and Visualization Techniques	Journal of Medical Internet Research
65	Cheng et al.	2017	Assessing Suicide Risk and Emotional Distress in Chinese Social Media: A Text Mining and Machine Learning Study	Journal of Medical Internet Research
66	Cheng et al.	2018	Analyzing Twitter as a Platform for Alzheimer-Related Dementia Awareness: Thematic Analyses of Tweets	JMIR Aging
67	Cherian et al.	2018	Representations of Codeine Misuse on Instagram: Content Analysis	JMIR Public Health and Surveillance
68	Chew & Eysenbach	2010	Pandemics in the age of Twitter: content analysis of Tweets during the 2009 H1N1 outbreak.	PLoS One
69	Chomutare et al.	2011	Features of Mobile Diabetes Applications: Review of the Literature and Analysis of Current Applications Compared Against Evidence-Based Guidelines	Journal of Medical Internet Research
70	Christmann et al.	2017	Stress Management Apps With Regard to Emotion-Focused Coping and Behavior Change Techniques: A Content Analysis	JMIR MHEALTH AND UHEALTH
71	Chu et al.	2015	Electronic Cigarette Marketing Online: a Multi-Site, Multi-Product Comparison	JMIR Public Health and Surveillance
72	Clyne et al.	2018	Using Social Media to Generate and Collect Primary Data: The #ShowsWorkplaceCompassion Twitter Research Campaign	JMIR Public Health and Surveillance
73	Colditz et al.	2018	Toward Real-Time Infoveillance of Twitter Health Messages.	Americal Journal of Public Health
74	Cole-Lewis et al.	2015	Social Listening: A Content Analysis of E-Cigarette Discussions on Twitter	Journal of Medical Internet Research

	Authors	Year	Title	Journal
75	Cole-Lewis et al.	2015	Assessing Electronic Cigarette-Related Tweets for Sentiment and Content Using Supervised Machine Learning	Journal of Medical Internet Research
76	Conway	2014	Ethical Issues in Using Twitter for Public Health Surveillance and Research: Developing a Taxonomy of Ethical Concepts From the Research Literature	Journal of Medical Internet Research
77	Cortés et al.	2017	Twitter for marijuana infodemiology	IEEE
78	Daniulaittyte et al.	2016	"When 'Bad' is 'Good'": Identifying Personal Communication and Sentiment in Drug-Related Tweets	JMIR Public Health and Surveillance
79	Davis et al.	2017	Public Response to Obamacare on Twitter	Journal of Medical Internet Research
80	de Viron et al.	2013	Communicating Genetics and Smoking Through Social Media: Are We There Yet?	Journal of Medical Internet Research
81	Dejohn et al.	2018	Identifying and Understanding Communities Using Twitter to Connect About Depression: Cross-Sectional Study	JMIR Mental Health
82	Delaney et al.	2014	Using a Geolocation Social Networking Application to Calculate the Population Density of Sex-Seeking Gay Men for Research and Prevention Services	Journal of Medical Internet Research
83	Delir Haghighi et al.	2017	Investigating Subjective Experience and the Influence of Weather Among Individuals With Fibromyalgia: A Content Analysis of Twitter.	JMIR Public Health and Surveillance
84	Doan et al.	2017	How Do You #relax When You're #stressed? A Content Analysis and Infodemiology Study of Stress-Related Tweets.	JMIR Public Health and Surveillance
85	Domnich et al.	2014	Demand-based web surveillance of sexually transmitted infections in Russia	International Journal of Public Health
86	Du et al.	2016	Gordie Howe's "Miraculous Treatment": Case Study of Twitter Users' Reactions to a Sport Celebrity's Stem Cell Treatment.	JMIR Public Health and Surveillance
87	Du et al.	2018	Public Perception Analysis of Tweets During the 2015 Measles Outbreak: Comparative Study Using Convolutional Neural Network Models	Journal of Medical Internet Research
88	Duke et al.	2014	The Use of Social Media by State Tobacco Control Programs to Promote Smoking Cessation: A Cross-Sectional Study	Journal of Medical Internet Research
89	Dunn et al.	2015	Associations Between Exposure to and Expression of Negative Opinions About Human Papillomavirus Vaccines on Social Media: An Observational Study	Journal of Medical Internet Research
90	Dyson et al.	2017	Social Media for the Dissemination of Cochrane Child Health Evidence: Evaluation Study	Journal of Medical Internet Research
91	Edney et al.	2018	Creating Engaging Health Promotion Campaigns on Social Media: Observations and Lessons From Fitbit and Garmin	Journal of Medical Internet Research
92	Eklund	2012	Tracking changes in search behaviour at a health web site	Studies in Health Technology and Informatics
93	Espina & Estuar	2017	Infodemiology for Syndromic Surveillance of Dengue and Typhoid Fever in the Philippines	Procedia Computer Science
94	Espina et al.	2016	Towards an Infodemiological Algorithm for Classification of Filipino Health Tweets	Procedia Computer Science
95	Eysenbach	2011	Infodemiology and infoveillance tracking online health information and cyberbehavior for public health.	American Journal of Preventive Medicine
96	Eysenbach	2009	Infodemiology and infoveillance: framework for an emerging set of public health informatics methods to analyze search, communication and publication behavior on the Internet.	Journal of Medical Internet Research
97	Farhadloo et al.	2018	Associations of Topics of Discussion on Twitter With Survey Measures of Attitudes, Knowledge, and Behaviors Related to Zika: Probabilistic Study in the United States	JMIR Public Health and Surveillance
98	Foroughi et al.	2016	Googling" for Cancer: An Infodemiological Assessment of Online Search Interests in Australia, Canada, New Zealand, the United Kingdom, and the United States.	JMIR Cancer
99	Gabarron et al. []	2014	Tweet Content Related to Sexually Transmitted Diseases: No Joking Matter	Journal of Medical Internet Research
100	Gabarron et al.	2015	Is There a Weekly Pattern for Health Searches on Wikipedia and Is the Pattern Unique to Health Topics?	Journal of Medical Internet Research
101	García-Díaz et al.	2018	Opinion mining for measuring the social perception of infectious diseases. an infodemiology approach	Communications in Computer and Information Science
102	Gayle et al.	2017	Public Response to Scientific Misconduct: Assessing Changes in Public Sentiment Toward the Stimulus-Triggered Acquisition of Pluripotency (STAP) Cell Case via Twitter	JMIR Public Health and Surveillance
103	Genes et al.	2017	Analysis of Twitter Users' Sharing of Official New York Storm Response Messages	Medicine 2.0
104	Gianfredi et al.	2018	Monitoring public interest toward pertussis outbreaks: an extensive Google Trends-based analysis.	Public Health
105	Gianfredi et al.	2018	Harnessing Big Data for Communicable Tropical and Sub-Tropical Disorders: Implications From a Systematic Review of the Literature.	Frontiers in Public Health
106	Giat & Yom-Tov	2018	Evidence From Web-Based Dietary Search Patterns to the Role of B12 Deficiency in Non-Specific Chronic Pain: A Large-Scale Observational Study	Journal of Medical Internet Research
107	Gittelman et al.	2015	A New Source of Data for Public Health Surveillance: Facebook Likes	Journal of Medical Internet Research
108	Gohil et al.	2018	Sentiment Analysis of Health Care Tweets: Review of the Methods Used	JMIR Public Health and Surveillance
109	Gough et al.	2017	Tweet for Behavior Change: Using Social Media for the Dissemination of Public Health Messages	JMIR Public Health and Surveillance
110	Grajales et al.	2014	Social Media: A Review and Tutorial of Applications in Medicine and Health Care	Journal of Medical Internet Research
111	Greaves et al.	2013	Use of Sentiment Analysis for Capturing Patient Experience From Free-Text Comments Posted Online	Journal of Medical Internet Research
112	Griffis et al.	2014	Use of Social Media Across US Hospitals: Descriptive Analysis of Adoption and Utilization	Journal of Medical Internet Research
113	Gruzd & Haythornthwaite	2013	Enabling Community Through Social Media	Journal of Medical Internet Research
114	Gu et al.	2014	Importance of Internet Surveillance in Public Health Emergency Control and Prevention: Evidence From a Digital Epidemiologic Study During Avian Influenza A H7N9 Outbreaks	Journal of Medical Internet Research

	Authors	Year	Title	Journal
115	Guy et al.	2012	Social media: A systematic review to understand the evidence and application in infodemiology	Lecture Notes of the Institute for Computer Sciences
116	Hamad et al.	2016	Toward a Mixed-Methods Research Approach to Content Analysis in The Digital Age: The Combined Content-Analysis Model and its Applications to Health Care Twitter Feeds.	Journal of Medical Internet Research
117	Hammer	2017	Ethical Considerations When Using Social Media for Research.	Oncology Nursung Forum
118	Hand et al.	2016	Assessing the Viability of Social Media for Disseminating Evidence-Based Nutrition Practice Guideline Through Content Analysis of Twitter Messages and Health Professional Interviews: An Observational Study	Journal of Medical Internet Research
119	Hanson et al.	2013	Tweaking and Tweeting: Exploring Twitter for Nonmedical Use of a Psychostimulant Drug (Adderall) Among College Students	Journal of Medical Internet Research
120	Hanson et al.	2013	An Exploration of Social Circles and Prescription Drug Abuse Through Twitter	Journal of Medical Internet Research
121	Harris et al. []	2014	Are Public Health Organizations Tweeting to the Choir? Understanding Local Health Department Twitter Followership	Journal of Medical Internet Research
122	Harris et al.	2014	Tweeting for and Against Public Health Policy: Response to the Chicago Department of Public Health's Electronic Cigarette Twitter Campaign	Journal of Medical Internet Research
123	Hébert et al.	2017	Online Dissemination Strategies of a Canada Research Chair: Overview and Lessons Learned	JMIR Research Protocols
124	Hendriks et al.	2018	Social Drinking on Social Media: Content Analysis of the Social Aspects of Alcohol-Related Posts on Facebook and Instagram	Journal of Medical Internet Research
125	Hill et al.	2011	Natural supplements for H1N1 influenza: retrospective observational infodemiology study of information and search activity on the Internet.	Journal of Medical Internet Research
126	Hingle et al.	2013	Collection and Visualization of Dietary Behavior and Reasons for Eating Using Twitter	Journal of Medical Internet Research
127	Hswen et al.	2018	Monitoring Online Discussions About Suicide Among Twitter Users With Schizophrenia: Exploratory Study	JMIR Mental Health
128	Huang et al.	2018	Public Opinions Toward Diseases: Infodemiological Study on News Media Data	Journal of Medical Internet Research
129	Huesch et al.	2017	Frequencies of Private Mentions and Sharing of Mammography and Breast Cancer Terms on Facebook: A Pilot Study	Journal of Medical Internet Research
130	Jankowski & Hofffmann	2016	Can Google Searches Predict the Popularity and Harm of Psychoactive Agents?	Journal of Medical Internet Research
131	Jones et al.	2018	Novel Approach to Cluster Patient-Generated Data Into Actionable Topics: Case Study of a Web-Based Breast Cancer Forum.	JMIR Medical Informatics
132	Jung et al.	2015	Identifying Key Hospital Service Quality Factors in Online Health Communities	Journal of Medical Internet Research
133	Jung et al.	2017	Ontology-Based Approach to Social Data Sentiment Analysis: Detection of Adolescent Depression Signals	Journal of Medical Internet Research
134	Kadry et al.	2011	Analysis of 4999 Online Physician Ratings Indicates That Most Patients Give Physicians a Favorable Rating	Journal of Medical Internet Research
135	Kagashe et al.	2017	Enhancing Seasonal Influenza Surveillance: Topic Analysis of Widely Used Medicinal Drugs Using Twitter Data	Journal of Medical Internet Research
136	Kalf et al.	2018	Use of Social Media in the Assessment of Relative Effectiveness: Explorative Review With Examples From Oncology	JMIR Cancer
137	Kandadai et al.	2016	Measuring Health Information Dissemination and Identifying Target Interest Communities on Twitter: Methods Development and Case Study of the @SafetyMD Network	JMIR Research Protocols
138	Kandula et al.	2017	Subregional Nowcasts of Seasonal Influenza Using Search Trends.	Journal of Medical Internet Research
139	Katsuki et al.	2015	Establishing a Link Between Prescription Drug Abuse and Illicit Online Pharmacies: Analysis of Twitter Data.	Journal of Medical Internet Research
140	Keller et al.	2018	Reproductive Health and Medication Concerns for Patients With Inflammatory Bowel Disease: Thematic and Quantitative Analysis Using Social Listening.	Journal of Medical Internet Research
141	Keller et al.	2017	Public Perceptions Regarding Use of Virtual Reality in Health Care: A Social Media Content Analysis Using Facebook	Journal of Medical Internet Research
142	Kendra et al.	2015	Characterizing the Discussion of Antibiotics in the Twittersphere: What is the Bigger Picture?	Journal of Medical Internet Research
143	Khan et al.	2012	A robust and scalable framework for detecting self-reported illness from twitter	IEEE
144	Khoury et al.	2012	Knowledge integration at the center of genomic medicine.	Genetics in Medicine
145	Kim et al.	2015	Using Twitter Data to Gain Insights into E-cigarette Marketing and Locations of Use: An Infoveillance Study.	Journal of Medical Internet Research
146	Kim et al.	2014	Investigating the congruence of crowdsourced information with official government data: the case of pediatric clinics.	Journal of Medical Internet Research
147	Kim et al.	2017	Scaling Up Research on Drug Abuse and Addiction Through Social Media Big Data.	Journal of Medical Internet Research
148	Kim et al.	2016	Garbage in, Garbage Out: Data Collection, Quality Assessment and Reporting Standards for Social Media Data Use in Health Research, Infodemiology and Digital Disease Detection.	Journal of Medical Internet Research
149	Kim et al.	2017	Classification of Twitter Users Who Tweet About E-Cigarettes	JMIR Public Health and Surveillance
150	Klembczyk et al.	2016	Google Flu Trends Spatial Variability Validated Against Emergency Department Influenza-Related Visits.	Journal of Medical Internet Research
151	Koh et al.	2014	Stroke Experiences in Weblogs: A Feasibility Study of Sex Differences	Journal of Medical Internet Research
152	Konheim-Kalkstein et al.	2018	How Women Evaluate Birth Challenges: Analysis of Web-Based Birth Stories	JMIR Pediatrics and Parenting
153	Koschack et al.	2015	Scientific Versus Experiential Evidence: Discourse Analysis of the Chronic Cerebrospinal Venous Insufficiency Debate in a Multiple Sclerosis Forum	Journal of Medical Internet Research

	Authors	Year	Title	Journal
154	Kostkova et al.	2013	Major Infection Events Over 5 Years: How Is Media Coverage Influencing Online Information Needs of Health Care Professionals and the Public?	Journal of Medical Internet Research
155	Krueger & Young	2015	Twitter: A Novel Tool for Studying the Health and Social Needs of Transgender Communities	JMIR Mental Health
156	Kurzinger et al.	2018	Web-Based Signal Detection Using Medical Forums Data in France: Comparative Analysis	Journal of Medical Internet Research
157	Lachmar et al.	2017	#MyDepressionLooksLike: Examining Public Discourse About Depression on Twitter	JMIR Mental Health
158	Lama et al.	2018	Discordance Between Human Papillomavirus Twitter Images and Disparities in Human Papillomavirus Risk and Disease in the United States: Mixed-Methods Analysis	Journal of Medical Internet Research
159	Lardon et al.	2015	Adverse Drug Reaction Identification and Extraction in Social Media: A Scoping Review	Journal of Medical Internet Research
160	Lau et al.	2011	The role of social media for patients and consumer health. Contribution of the IMIA Consumer Health Informatics Working Group.	Yearbook of Medical Informatics
161	Lavorgna et al.	2018	e-Health and multiple sclerosis: An update.	Multiple Sclerosis
162	Lazard et al.	2016	E-Cigarette Social Media Messages: A Text Mining Analysis of Marketing and Consumer Conversations on Twitter	JMIR Public Health and Surveillance
163	Leal Neto et al.	2017	Saúde na Copa: The World's First Application of Participatory Surveillance for a Mass Gathering at FIFA World Cup 2014, Brazil	JMIR Public Health and Surveillance
164	Lee et al.	2014	What Are Health-Related Users Tweeting? A Qualitative Content Analysis of Health-Related Users and Their Messages on Twitter	Journal of Medical Internet Research
165	Lee et al.	2016	Examining the Relationship Between Past Orientation and US Suicide Rates: An Analysis Using Big Data-Driven Google Search Queries	Journal of Medical Internet Research
166	Lenoir et al.	2017	Raising Awareness About Cervical Cancer Using Twitter: Content Analysis of the 2015 #SmearForSmear Campaign	Journal of Medical Internet Research
167	Leung et al.	2018	Social Media Users' Perception of Telemedicine and mHealth in China: Exploratory Study	JMIR mHealth and uHealth
168	Li et al.	2018	Understanding Users' Vaping Experiences from Social Media: Initial Study Using Sentiment Opinion Summarization Techniques.	Journal of Medical Internet Research
169	Liang & Scammon	2013	Incidence of Online Health Information Search: A Useful Proxy for Public Health Risk Perception	Journal of Medical Internet Research
170	Lienemann et al.	2017	Methods for Coding Tobacco-Related Twitter Data: A Systematic Review	Journal of Medical Internet Research
171	Ling & Lee	2016	Disease Monitoring and Health Campaign Evaluation Using Google Search Activities for HIV and AIDS, Stroke, Colorectal Cancer, and Marijuana Use in Canada: A Retrospective Observational Study.	JMIR Public Health and Surveillance
172	Liu et al.	2011	The quality and characteristics of leading general hospitals' websites in China.	Journal of Medical Systems
173	Liu et al.	2016	Use of Social Media in the Diabetes Community: An Exploratory Analysis of Diabetes-Related Tweets	JMIR Diabetes
174	Liu et al.	2017	Identifying Potential Norovirus Epidemics in China via Internet Surveillance	Journal of Medical Internet Research
175	Liu et al.	2017	Using Real-Time Social Media Technologies to Monitor Levels of Perceived Stress and Emotional State in College Students: A Web-Based Questionnaire Study	JMIR Mental Health
176	Liu et al.	2018	Monitoring Freshman College Experience Through Content Analysis of Tweets: Observational Study	JMIR Public Health and Surveillance
177	Livelo & Cheng	2018	Intelligent dengue infoveillance using gated recurrent neural learning and cross-label frequencies	IEEE International Conference on Agents
178	Lu et al.	2018	Accurate Influenza Monitoring and Forecasting Using Novel Internet Data Streams: A Case Study in the Boston Metropolis	JMIR Public Health and Surveillance
179	Lyles et al.	2016	Applying Sparse Machine Learning Methods to Twitter: Analysis of the 2012 Change in Pap Smear Guidelines. A Sequential Mixed-Methods Study	JMIR Public Health and Surveillance
180	Mackey & Liang	2013	Global Reach of Direct-to-Consumer Advertising Using Social Media for Illicit Online Drug Sales	Journal of Medical Internet Research
181	Mackey et al.	2018	Solution to Detect, Classify, and Report Illicit Online Marketing and Sales of Controlled Substances via Twitter: Using Machine Learning and Web Forensics to Combat Digital Opioid Access	Journal of Medical Internet Research
182	Madden et al.	2017	The Seasonal Periodicity of Healthy Contemplations About Exercise and Weight Loss: Ecological Correlational Study	JMIR Public Health and Surveillance
183	Mahoney et al.	2015	The Digital Distribution of Public Health News Surrounding the Human Papillomavirus Vaccination: A Longitudinal Infodemiology Study.	JMIR Public Health and Surveillance
184	Mahroum et al.	2018	An infodemiological investigation of the so-called "Fluad effect" during the 2014/2015 influenza vaccination campaign in Italy: Ethical and historical implications.	Human Vaccines and Immunotherapeutics
185	Majumder et al.	2016	Utilizing Nontraditional Data Sources for Near Real-Time Estimation of Transmission Dynamics During the 2015-2016 Colombian Zika Virus Disease Outbreak	JMIR Public Health and Surveillance
186	Manchaiah et al.	2018	Representation of Tinnitus in the US Newspaper Media and in Facebook Pages: Cross-Sectional Analysis of Secondary Data	Interactive Journal of Medical Research
187	Mao et al.	2014	An Internet-Based Epidemiological Investigation of the Outbreak of H7N9 Avian Influenza A in China Since Early 2013	Journal of Medical Internet Research
188	Marcon et al.	2016	Chiropractic and Spinal Manipulation Therapy on Twitter: Case Study Examining the Presence of Critiques and Debates.	JMIR Public Health and Surveillance
189	Marcus et al.	2012	What Are Young Adults Saying About Mental Health? An Analysis of Internet Blogs	Journal of Medical Internet Research
190	Martinez et al.	2017	iOS Appstore-Based Phone Apps for Diabetes Management: Potential for Use in Medication Adherence	JMIR Diabetes
191	Martinez-Arroyo et al.	2018	Potential uses of an infodemiology approach for health-care services for rheumatology.	Clinical Rheumatology
192	Martinez-Millana et al	2017	Evaluating the Social Media Performance of Hospitals in Spain: A Longitudinal and Comparative Study	Journal of Medical Internet Research
193	Martins-Filho et al.	2018	Femicide trends in Brazil: relationship between public interest and mortality rates.	Archives of Womens Mental Health

	Authors	Year	Title	Journal
194	Massey et al.	2016	Applying Multiple Data Collection Tools to Quantify Human Papillomavirus Vaccine Communication on Twitter	Journal of Medical Internet Research
195	Matsuda et al.	2017	Analysis of Patient Narratives in Disease Blogs on the Internet: An Exploratory Study of Social Pharmacovigilance	JMIR Public Health and Surveillance
196	Mavragani & Ochoa	2018	Forecasting AIDS prevalence in the United States using online search traffic data	Journal of Big Data
197	Mavragani & Ochoa	2018	Infoveillance of infectious diseases in USA: STDs, tuberculosis, and hepatitis	Journal of Big Data
198	Mavragani et al.	2018	Assessing the Methods, Tools, and Statistical Approaches in Google Trends Research: Systematic Review.	Journal of Medical Internet Research
199	Mavragani et al.	2018	Integrating Smart Health in the US Health Care System: Infodemiology Study of Asthma Monitoring in the Google Era.	JMIR Public Health and Surveillance
200	Mazzocut et al.	2016	Web Conversations About Complementary and Alternative Medicines and Cancer: Content and Sentiment Analysis	Journal of Medical Internet Research
201	McNaughton et al.	2014	Monitoring of Internet Forums to Evaluate Reactions to the Introduction of Reformulated OxyContin to Deter Abuse	Journal of Medical Internet Research
202	Meaney et al.	2016	Reaction on Twitter to a Cluster of Perinatal Deaths: A Mixed Method Study.	JMIR Public Health and Surveillance
203	Mejova et al.	2018	Online Health Monitoring using Facebook Advertisement Audience Estimates in the United States: Evaluation Study.	JMIR Public Health and Surveillance
204	Melver et al.	2015	Characterizing Sleep Issues Using Twitter	Journal of Medical Internet Research
205	Menachemi et al.	2017	Using Web-Based Search Data to Study the Public's Reactions to Societal Events: The Case of the Sandy Hook Shooting.	JMIR Public Health and Surveillance
206	Mendiola et al.	2015	Valuable Features in Mobile Health Apps for Patients and Consumers: Content Analysis of Apps and User Ratings	JMIR mHealth and uHealth
207	Metwally et al.	2017	Using Social Media to Characterize Public Sentiment Toward Medical Interventions Commonly Used for Cancer Screening: An Observational Study	Journal of Medical Internet Research
208	Miller et al.	2017	What Are People Tweeting About Zika? An Exploratory Study Concerning Its Symptoms, Treatment, Transmission, and Prevention	JMIR Public Health and Surveillance
209	Mishori et al.	2014	Mapping physician Twitter networks: describing how they work as a first step in understanding connectivity, information flow, and message diffusion.	Journal of Medical Internet Research
210	Mnadla et al.	2016	Infodemiological data of Ironman Triathlon in the study period 2004-2013.	Data in Brief
211	Moccia et al.	2018	Neurology and the Internet: a review.	Neurological Sciences
212	Mollema et al.	2015	Disease Detection or Public Opinion Reflection? Content Analysis of Tweets, Other Social Media, and Online Newspapers During the Measles Outbreak in the Netherlands in 2013	Journal of Medical Internet Research
213	Mowery et al.	2017	Understanding Depressive Symptoms and Psychosocial Stressors on Twitter: A Corpus-Based Study	Journal of Medical Internet Research
214	Mukhija et al.	2017	Effectivity of Awareness Months in Increasing Internet Search Activity for Top Malignancies Among Women.	JMIR Public Health and Surveillance
215	Muralidhara & Paul	2018	#Healthy Selfies: Exploration of Health Topics on Instagram	JMIR Public Health and Surveillance
216	Myslin et al.	2013	Using Twitter to Examine Smoking Behavior and Perceptions of Emerging Tobacco Products	Journal of Medical Internet Research
217	Nagar et al.	2014	A case study of the New York City 2012-2013 influenza season with daily geocoded Twitter data from temporal and spatiotemporal perspectives.	Journal of Medical Internet Research
218	Nagel et al.	2013	The complex relationship of realspace events and messages in cyberspace: case study of influenza and pertussis using tweets.	Journal of Medical Internet Research
219	Nakada et al.	2014	Development of a national agreement on human papillomavirus vaccination in Japan: an infodemiology study.	Journal of Medical Internet Research
220	Nakhasi et al.	2014	Online Social Networks That Connect Users to Physical Activity Partners: A Review and Descriptive Analysis	Journal of Medical Internet Research
221	Nascimento et al.	2014	Real-time sharing and expression of migraine headache suffering on Twitter: a cross-sectional infodemiology study.	Journal of Medical Internet Research
222	Nguyen et al.	2016	Building a National Neighborhood Dataset From Geotagged Twitter Data for Indicators of Happiness, Diet, and Physical Activity	JMIR Public Health and Surveillance
223	Nishimoto et al.	2016	Estimating the Duration of Public Concern After the Fukushima Dai-ichi Nuclear Power Station Accident From the Occurrence of Radiation Exposure-Related Terms on Twitter: A Retrospective Data Analysis.	JMIR Public Health and Surveillance
224	Noll-Hussong	2017	Whiplash Syndrome Reloaded: Digital Echoes of Whiplash Syndrome in the European Internet Search Engine Context.	JMIR Public Health and Surveillance
225	Nsoesie et al.	2014	Guess Who's Not Coming to Dinner? Evaluating Online Restaurant Reservations for Disease Surveillance	Journal of Medical Internet Research
226	Odlum et al.	2018	How Twitter Can Support the HIV/AIDS Response to Achieve the 2030 Eradication Goal: In-Depth Thematic Analysis of World AIDS Day Tweets.	JMIR Public Health and Surveillance
227	Oldroyd et al.	2018	Identifying Methods for Monitoring Foodborne Illness: Review of Existing Public Health Surveillance Techniques.	JMIR Public Health and Surveillance
228	Oser et al.	2017	A Novel Approach to Identifying Barriers and Facilitators in Raising a Child With Type 1 Diabetes: Qualitative Analysis of Caregiver Blogs	JMIR Diabetes
229	Ozan-Rafferty	2014	In the Words of the Medical Tourist: An Analysis of Internet Narratives by Health Travelers to Turkey	Journal of Medical Internet Research
230	Pan et al.	2018	The Significance of Witness Sensors for Mass Casualty Incidents and Epidemic Outbreaks	Journal of Medical Internet Research
231	Park & Hong	2018	Identification of Primary Medication Concerns Regarding Thyroid Hormone Replacement Therapy From Online Patient Medication Reviews: Text Mining of Social Network Data	Journal of Medical Internet Research
232	Peiper et al.	2017	Patterns of Twitter Behavior Among Networks of Cannabis Dispensaries in California	Journal of Medical Internet Research

	Authors	Year	Title	Journal
233	Pervaiz et al.	2012	FluBreaks: Early Epidemic Detection from Google Flu Trends	Journal of Medical Internet Research
234	Pesala et al.	2017	Health Information-Seeking Patterns of the General Public and Indications for Disease Surveillance: Register-Based Study Using Lyme Disease.	JMIR Public Health and Surveillance
235	Pesala et al.	2017	Health Care Professionals' Evidence-Based Medicine Internet Searches Closely Mimic the Known Seasonal Variation of Lyme Borreliosis: A Register-Based Study	JMIR Public Health and Surveillance
236	Phillips et al.	2018	Relationship Between State-Level Google Online Search Volume and Cancer Incidence in the United States: Retrospective Study.	Journal of Medical Internet Research
237	Poirier et al.	2018	Real Time Influenza Monitoring Using Hospital Big Data in Combination with Machine Learning Methods: Comparison Study.	JMIR Public Health and Surveillance
238	Pretorius et al.	2018	Sudden Infant Death Syndrome and Safe Sleep on Twitter: Analysis of Influences and Themes to Guide Health Promotion Efforts	JMIR Pediatrics and Parenting
239	Priest et al.	2016	Finding the Patient's Voice Using Big Data: Analysis of Users' Health-Related Concerns in the ChaCha Question-and-Answer Service (2009-2012).	Journal of Medical Internet Research
240	Rabarison et al.	2017	Measuring Audience Engagement for Public Health Twitter Chats: Insights From #LiveFitNOLA	JMIR Public Health and Surveillance
241	Radin & Sciascia	2017	Infodemiology of systemic lupus erythematous using Google Trends.	Lupus
242	Radzikowski et al.	2016	The Measles Vaccination Narrative in Twitter: A Quantitative Analysis	JMIR Public Health and Surveillance
243	Ragestar-Mojarad et al.	2016	Using Social Media Data to Identify Potential Candidates for Drug Repurposing: A Feasibility Study	JMIR Research Protocols
244	Rastegar-Mojarad et al.	2015	Collecting and Analyzing Patient Experiences of Health Care From Social Media	JMIR Research Protocols
245	Ricard et al.	2018	Exploring the Utility of Community-Generated Social Media Content for Detecting Depression: An Analytical Study on Instagram	Journal of Medical Internet Research
246	Risson et al.	2016	Patterns of Treatment Switching in Multiple Sclerosis Therapies in US Patients Active on Social Media: Application of Social Media Content Analysis to Health Outcomes Research	Journal of Medical Internet Research
247	Roberts et al.	2015	Globalization of Continuing Professional Development by Journal Clubs via Microblogging: A Systematic Review	Journal of Medical Internet Research
248	Robillard et al.	2013	Utilizing Social Media to Study Information-Seeking and Ethical Issues in Gene Therapy	Journal of Medical Internet Research
249	Roccetti et al.	2017	Attitudes of Crohn's Disease Patients: Infodemiology Case Study and Sentiment Analysis of Facebook and Twitter Posts.	JMIR Public Health and Surveillance
250	Rocheleau et al.	2015	An Observational Study of Social and Emotional Support in Smoking Cessation Twitter Accounts: Content Analysis of Tweets	Journal of Medical Internet Research
251	Rose et al.	2017	Perceptions of Menthol Cigarettes Among Twitter Users: Content and Sentiment Analysis	Journal of Medical Internet Research
252	Rosenblum & Yom-Tov	2017	Seeking Web-Based Information About Attention Deficit Hyperactivity Disorder: Where, What, and When	Journal of Medical Internet Research
253	Sadah et al.	2015	A Study of the Demographics of Web-Based Health-Related Social Media Users	Journal of Medical Internet Research
254	Sadah et al.	2016	Demographic-Based Content Analysis of Web-Based Health-Related Social Media	Journal of Medical Internet Research
255	Saha et al.	2017	Characterizing Awareness of Schizophrenia Among Facebook Users by Leveraging Facebook Advertisement Estimates	Journal of Medical Internet Research
256	Samaras et al.	2017	Syndromic Surveillance Models Using Web Data: The Case of Influenza in Greece and Italy Using Google Trends	JMIR Public Health and Surveillance
257	Santos & Matos	2014	Analysing Twitter and web queries for flu trend prediction.	Theoretical Biology and Medical Modelling
258	Sanz-Lorente et al.	2018	Web 2.0 Tools in the Prevention of Curable Sexually Transmitted Diseases: Scoping Review	Journal of Medical Internet Research
259	Sarker et al.	2017	Discovering Cohorts of Pregnant Women From Social Media for Safety Surveillance and Analysis	Journal of Medical Internet Research
260	Sato et al.	2015	Blog Posting After Lung Cancer Notification: Content Analysis of Blogs Written by Patients or Their Families	JMIR Cancer
261	Schlichthorst et al.	2018	Influencing the Conversation About Masculinity and Suicide: Evaluation of the Man Up Multimedia Campaign Using Twitter Data	JMIR Mental Health
262	Sciascia & Radin	2017	What can Google and Wikipedia can tell us about a disease? Big Data trends analysis in Systemic Lupus Erythematosus.	International Journal of Medical Informatics
263	Sciascia et al.	2018	Infodemiology of antiphospholipid syndrome: Merging informatics and epidemiology.	European Journal of Rheumatology
264	Seabrook et al.	2018	Predicting Depression From Language-Based Emotion Dynamics: Longitudinal Analysis of Facebook and Twitter Status Updates	Journal of Medical Internet Research
265	Seidl et al.	2018	What Do Germans Want to Know About Skin Cancer? A Nationwide Google Search Analysis From 2013 to 2017	Journal of Medical Internet Research
266	Sentana-Lledo et al.	2016	Seasons, Searches, and Intentions: What The Internet Can Tell Us About The Bed Bug (Hemiptera: Cimicidae) Epidemic.	Journal of Medical Entomology
267	Seo et al.	2014	Cumulative Query Method for Influenza Surveillance Using Search Engine Data	Journal of Medical Internet Research
268	Sewalk et al.	2018	Using Twitter to Examine Web-Based Patient Experience Sentiments in the United States: Longitudinal Study	Journal of Medical Internet Research
269	SeyyedHosseini et al.	2018	An infodemiology study on breast cancer in Iran: Health information supply versus health information demand in PubMed and Google Trends	Electronic Library
270	SeyyedHosseini et al.	2017	Infodemiology: A new presence concept in human information interaction based on eysenbach's view	Iranian Journal of Information Processing Management
271	SeyyedHosseini et al.	2017	Scientific publication behavior versus information seeking behavior: An infodemiological study on stomach cancer	Webology
272	Sharpe et al.	2016	Evaluating Google, Twitter, and Wikipedia as Tools for Influenza Surveillance Using Bayesian Change Point Analysis: A Comparative Analysis	Journal of Medical Internet Research

	Authors	Year	Title	Journal
273	Shi & Salmon	2018	Identifying Opinion Leaders to Promote Organ Donation on Social Media: Network Study	Journal of Medical Internet Research
274	Simpson et al.	2018	Detecting Novel and Emerging Drug Terms Using Natural Language Processing: A Social Media Corpus Study	Journal of Medical Internet Research
275	Sinha et al.	2018	Social Media Impact of the Food and Drug Administration's Drug Safety Communication Messaging About Zolpidem: Mixed-Methods Analysis	Journal of Medical Internet Research
276	Sinnenberg et al.	2018	Content Analysis of Metaphors About Hypertension and Diabetes on Twitter: Exploratory Mixed-Methods Study	JMIR Diabetes
277	Smith et al.	2017	Variations in Facebook Posting Patterns Across Validated Patient Health Conditions: A Prospective Cohort Study	Journal of Medical Internet Research
278	Spyropoulos et al.	2018	Uptake and Utilization of the Management of Anticoagulation in the Periprocedural Period App: Longitudinal Analysis	JMIR mHeath and uHealth
279	Staal et al.	2018	New Tobacco and Tobacco-Related Products: Early Detection of Product Development, Marketing Strategies, and Consumer Interest.	JMIR Public Health and Surveillance
280	Stefanidis et al.	2017	Zika in Twitter: Temporal Variations of Locations, Actors, and Concepts	JMIR Public Health and Surveillance
281	Sudau et al.	2014	Sources of Information and Behavioral Patterns in Online Health Forums: Observational Study	Journal of Medical Internet Research
282	Sueki	2015	The association of suicide-related Twitter use with suicidal behaviour: a cross-sectional study of young internet users in Japan.	Journal of Affective Disorders
283	Sugawara et al.	2016	Medical Institutions and Twitter: A Novel Tool for Public Communication in Japan	JMIR Public Health and Surveillance
284	Sugawara et al.	2017	Scientific Misconduct and Social Media: Role of Twitter in the Stimulus Triggered Acquisition of Pluripotency Cells Scandal	Journal of Medical Internet Research
285	Surian et al.	2016	Characterizing Twitter Discussions About HPV Vaccines Using Topic Modeling and Community Detection	Journal of Medical Internet Research
286	Tafti et al.	2017	Adverse Drug Event Discovery Using Biomedical Literature: A Big Data Neural Network Adventure	JMIR Medical Informatics
287	Tana et al.	2018	Diurnal Variations of Depression-Related Health Information Seeking: Case Study in Finland Using Google Trends Data.	JMIR Mental Health
288	Tangherlini et al.	2016	"Mommy Blogs" and the Vaccination Exemption Narrative: Results From A Machine-Learning Approach for Story Aggregation on Parenting Social Media Sites	JMIR Public Health and Surveillance
289	Tapi Nzali et al.	2017	What Patients Can Tell Us: Topic Analysis for Social Media on Breast Cancer	JMIR Medical Informatics
290	Thackeray et al.	2013	Analysis of the Purpose of State Health Departments' Tweets: Information Sharing, Engagement, and Action	Journal of Medical Internet Research
291	Tighe et al.	2015	The Painful Tweet: Text, Sentiment, and Community Structure Analyses of Tweets Pertaining to Pain	Journal of Medical Internet Research
292	Timpka et al.	2014	Performance of eHealth Data Sources in Local Influenza Surveillance: A 5-Year Open Cohort Study	Journal of Medical Internet Research
293	Tinschert et al.	2017	The Potential of Mobile Apps for Improving Asthma Self-Management: A Review of Publicly Available and Well-Adopted Asthma Apps	JMIR mhealth and uHealth
294	Tougas et al.	2018	Social Media Content About Children's Pain and Sleep: Content and Network Analysis	JMIR Pediatrics and Parenting
295	Triemstra et al.	2018	Correlations Between Hospitals' Social Media Presence and Reputation Score and Ranking: Cross-Sectional Analysis	Journal of Medical Internet Research
296	Troullos et al.	2014	Common Cold Symptoms in Children: Results of an Internet-Based Surveillance Program	Journal of Medical Internet Research
297	Tsuya et al.	2014	Do Cancer Patients Tweet? Examining the Twitter Use of Cancer Patients in Japan	Journal of Medical Internet Research
298	Tufts et al.	2018	Characterizing Tweet Volume and Content About Common Health Conditions Across Pennsylvania: Retrospective Analysis	JMIR Public Health and Surveillance
299	Tyrawski & DeAndrea	2015	Pharmaceutical Companies and Their Drugs on Social Media: A Content Analysis of Drug Information on Popular Social Media Sites	Journal of Medical Internet Research
300	Utengen et al.	2017	Patient Participation at Health Care Conferences: Engaged Patients Increase Information Flow, Expand Propagation, and Deepen Engagement in the Conversation of Tweets Compared to Physicians or Researchers	Journal of Medical Internet Research
301	van Lent et al.	2017	Too Far to Care? Measuring Public Attention and Fear for Ebola Using Twitter	Journal of Medical Internet Research
302	Vasconcellos-Silva et al.	2017	Using Google Trends Data to Study Public Interest in Breast Cancer Screening in Brazil: Why Not a Pink February?	JMIR Public Health and Surveillance
303	Vickey & Breslin	2017	Online Influence and Sentiment of Fitness Tweets: Analysis of Two Million Fitness Tweets	JMIR Public Health and Surveillance
304	Wagner et al.	2017	Estimating the Population Impact of a New Pediatric Influenza Vaccination Program in England Using Social Media Content	Journal of Medical Internet Research
305	Wakamiya	2018	Twitter-Based Influenza Detection After Flu Peak via Tweets With Indirect Information: Text Mining Study.	JMIR Public Health and Surveillance
306	Wang & Chen	2018	Economic Recession and Obesity-Related Internet Search Behavior in Taiwan: Analysis of Google Trends Data.	JMIR Public Health and Surveillance
307	Wang et al.	2015	Forecasting the Incidence of Dementia and Dementia-Related Outpatient Visits With Google Trends: Evidence From Taiwan	Journal of Medical Internet Research
308	Weeg et al.	2015	Using Twitter to Measure Public Discussion of Diseases: A Case Study	JMIR Public Health and Surveillance
309	Williams et al.	2013	How Twitter Is Studied in the Medical Professions: A Classification of Twitter Papers Indexed in PubMed	MECIDINE 2.0
310	Winchester et al.	2017	Quality of Social Media and Web-Based Information Regarding Inappropriate Nuclear Cardiac Stress Testing and the Choosing Wisely Campaign: A Cross-Sectional Study	Interactive Journal of Medical Research
311	Wittmeier et al.	2014	Analysis of a Parent-Initiated Social Media Campaign for Hirschsprung's Disease	Journal of Medical Internet Research
312	Wong et al.	2013	Accessing Suicide-Related Information on the Internet: A Retrospective Observational Study of Search Behavior	Journal of Medical Internet Research

	Authors	Year	Title	Journal
313	Wong et al.	2015	Twitter Sentiment Predicts Affordable Care Act Marketplace Enrollment	Journal of Medical Internet Research
314	Wongkoblap et al.	2017	Researching Mental Health Disorders in the Era of Social Media: Systematic Review.	Journal of Medical Internet Research
315	Woo et al.	2016	Estimating Influenza Outbreaks Using Both Search Engine Query Data and Social Media Data in South Korea.	Journal of Medical Internet Research
316	Wood et al.	2018	Public Awareness of Uterine Power Morcellation Through US Food and Drug Administration Communications: Analysis of Google Trends Search Term Patterns	JMIR Public Health and Surveillance
317	Xu & Liu	2015	mHealthApps: A Repository and Database of Mobile Health Apps	JMIR mhealth and uHealth
318	Xu et al.	2018	Predicting Prediabetes Through Facebook Postings: Protocol for a Mixed-Methods Study.	JMIR Research Protocols
319	Xu et al.	2016	Leveraging Social Media to Promote Public Health Knowledge: Example of Cancer Awareness via Twitter	JMIR Public Health and Surveillance
320	Yagahara et al.	2018	Relationships Among Tweets Related to Radiation: Visualization Using Co-Occurring Networks	JMIR Public Health and Surveillance
321	Yang et al.	2017	Effects of the Ambient Fine Particulate Matter on Public Awareness of Lung Cancer Risk in China: Evidence from the Internet-Based Big Data Platform	JMIR Public Health and Surveillance
322	Yin et al.	2015	A Scalable Framework to Detect Personal Health Mentions on Twitter.	Journal of Medical Internet Research
323	Yom-Tov & Gabrilovich	2013	Postmarket drug surveillance without trial costs: discovery of adverse drug reactions through large-scale analysis of web search queries.	Journal of Medical Internet Research
324	Yom-Tov et al.	2014	Seeking Insights About Cycling Mood Disorders via Anonymized Search Logs	Journal of Medical Internet Research
325	Yom-Tov et al.	2015	Automatic Identification of Web-Based Risk Markers for Health Events	Journal of Medical Internet Research
326	Yom-Tov et al.	2014	Detecting disease outbreaks in mass gatherings using Internet data.	Journal of Medical Internet Research
327	Yom-Tov Lev-Ran	2017	Adverse Reactions Associated With Cannabis Consumption as Evident From Search Engine Queries	JMIR Public Health and Surveillance
328	Young	2018	Social Media as a New Vital Sign: Commentary	Journal of Medical Internet Research
329	Zeraatkar & Ahmadi	2018	Trends of infodemiology studies: a scoping review.	Health Information and Libraries Journal
330	Zhan et al.	2017	Identifying Topics for E-Cigarette User-Generated Contents: A Case Study From Multiple Social Media Platforms.	Journal of Medical Internet Research
331	Zhang et al.	2016	Tracking Dabbing Using Search Query Surveillance: A Case Study in the United States	Journal of Medical Internet Research
332	Zhang et al.	2018	Automated Identification of Hookahs (Waterpipes) on Instagram: An Application in Feature Extraction Using Convolutional Neural Network and Support Vector Machine Classification	Journal of Medical Internet Research
333	Zhang et al.	2014	Methodology of developing a smartphone application for crisis research and its clinical application.	Technology and Health Care
334	Zhang et al.	2013	Electronic word of mouth on twitter about physical activity in the United States: exploratory infodemiology study.	Journal of Medical Internet Research
335	Zhao & Yang	2018	Drug Repositioning to Accelerate Drug Development Using Social Media Data: Computational Study on Parkinson Disease	Journal of Medical Internet Research
336	Zheluk et al.	2012	Searching for Truth: Internet Search Patterns as a Method of Investigating Online Responses to a Russian Illicit Drug Policy Debate	Journal of Medical Internet Research
337	Zheluk et al.	2013	Internet search patterns of human immunodeficiency virus and the digital divide in the Russian Federation: infoveillance study.	Journal of Medical Internet Research
338	Zheluk et al.	2014	Internet search and krokodil in the Russian Federation: an infoveillance study.	Journal of Medical Internet Research

Table A2 consists of the complete list of the 338 examined publications categorized by data source employed, i.e. Google, Twitter, Facebook, Instagram, Wikipedia, Other Social Media, Blogs/Forums/Communities, Websites/Platforms, News Outlets/Media, Electronic Health Records/Databases/Call Records/Online Surveys, Other Search Engines, Mobile Apps, and Reviews.

Table A2. List of publications by data source employed

	**Authors**	**Google**	**Twitter**	**Facebook**	**Instagram**	**Wikipedia**	**Other Social Media**	**Blogs/Forums/Communities**	**Websites/Platforms**	**News Outlets/Media**	**Electronic Health Records Databases/Call records/ Online Surveys**	**Other Search Engines**	**Online Survey**	**Mobile App**	**N/A**	**Review**
1	Abbate et al., 2017													✓		
2	Abbe & Falissard, 2017							✓								
3	Abdellaoui et al., 2017							✓								
4	Abdellaoui et al., 2018							✓								
5	Adams, 2013								✓							
6	Adawi et al., 2017	✓														
7	Adrover et al., 2015		✓													
8	Adusumalli et al., 2015								✓							
9	Agarwal et al., 2016											✓				
10	Albalawi & Sixsmith, 2015		✓							✓						
11	Allem et al., 2017				✓											
12	Allem et al., 2018		✓													
13	Allem et al., 2017		✓													
14	Allem et al., 2017		✓													
15	Alnemer et al., 2015		✓													
16	Alvarez-Mon et al., 2018		✓													
17	Alvaro et al., 2017		✓						✓							
18	Anderson et al., 2017							✓								
19	Aoki et al., 2018		✓													
20	Arnhold et al													✓		
21	Aslam et al., 2014		✓													
22	Athilingam & Jenkins													✓		✓
23	Ayers et al., 2012	✓								✓						
24	Ayers et al., 2016	✓	✓			✓				✓	✓					
25	Balls-Berry et al., 2018		✓	✓			✓									
26	Baltrusaitis et al., 2017								✓							
27	Ben-Sasson & Yom-Tov, 2016							✓								
28	Berlinberg et al., 2018	✓														
29	Bernardo et al., 2013															✓
30	Berry et al., 2017		✓													

	Authors	Google	Twitter	Facebook	Instagram	Wikipedia	Other Social Media	Blogs/Forums/Communities	Websites/Platforms	News Outlets/Media	Electronic Health Records Databases/Call records/ Online Surveys	Other Search Engines	Online Survey	Mobile App	N/A	Review
31	Bian et al., 2017		✓													
32	Birnbaum et al., 2017		✓													
33	Bollegala et al., 2018		✓													
34	Bousquet et al., 2017							✓	✓							
35	Bragazzi, 2013	✓														
36	Bragazzi, 2013	✓														
37	Bragazzi et al., 2016	✓														
38	Bragazzi et al., 2016	✓														
39	Bragazzi et al., 2016	✓														
40	Braithwaite et al., 2016		✓													
41	Brigo & Erro, 2016	✓				✓										
42	Brigo & Trinka, 2015	✓														
43	Brigo et al., 2015	✓							✓							
44	Brigo et al., 2014	✓														
45	Brigo et al., 2015					✓										
46	Brigo et al., 2018					✓										
47	Brigo et al., 2018					✓										
48	Brigo et al., 2014	✓														
49	Brigo et al., 2015					✓										
50	Brigo et al., 2016	✓														
51	Broniatowski et al., 2015	✓	✓													
52	Bubenzer, 2009														✓	
53	Burton et al., 2012	✓														
54	Callahan et al., 2015								✓							
55	Carrotte et al., 2017	✓	✓		✓		✓									
56	Cartwright et al., 2018	✓										✓				
57	Cavazos-Regh et al., 2014		✓													
58	Cawkwell et al., 2015							✓								
59	Chan et al., 2013							✓								
60	Chen & Dredze, 2018		✓													
61	Chen et al., 2018						✓									
62	Chen et al., 2018								✓							
63	Chen et al., 2018		✓													
64	Chen et al., 2015								✓							
65	Cheng et al., 2017								✓							

	Authors	Google	Twitter	Facebook	Instagram	Wikipedia	Other Social Media	Blogs/Forums/Communities	Websites/Platforms	News Outlets/Media	Electronic Health Records Databases/Call records/ Online Surveys	Other Search Engines	Online Survey	Mobile App	N/A	Review
66	Cheng et al., 2018		✓													
67	Cherian et al., 2018				✓											
68	Chew & Eysenbach, 2010		✓													
69	Chomutare et al., 2011													✓		
70	Christmann et al., 2017													✓		
71	Chu et al., 2015	✓	✓	✓	✓											
72	Clyne et al., 2018		✓													
73	Colditz et al., 2018		✓													
74	Cole-Lewis et al., 2015		✓													
75	Cole-Lewis et al., 2015		✓													
76	Conway, 2014								✓							
77	Cortés et al., 2017		✓													
78	Daniulaittyte et al., 2016		✓													
79	Davis et al., 2017		✓													
80	de Viron et al., 2013		✓	✓			✓									
81	Dejohn et al., 2018		✓													
82	Delaney et al., 2014													✓		
83	Delir Haghighi et al., 2017		✓													
84	Doan et al., 2017		✓													
85	Domnich et al., 2014											✓				
86	Du et al., 2016		✓													
87	Du et al., 2018		✓													
88	Duke et al., 2014	✓	✓	✓	✓		✓	✓								
89	Dunn et al., 2015		✓													
90	Dyson et al., 2017		✓					✓								
91	Edney et al., 2018		✓	✓	✓											
92	Eklund, 2012								✓							
93	Espina & Estuar, 2017		✓													
94	Espina et al., 2016		✓													
95	Eysenbach, 2011														✓	
96	Eysenbach, 2009														✓	
97	Farhadloo et al., 2018		✓													
98	Foroughi et al., 2016	✓														
99	Gabarron et al., 2014		✓													
100	Gabarron et al., 2015					✓										

	Authors	Google	Twitter	Facebook	Instagram	Wikipedia	Other Social Media	Blogs/Forums/Communities	Websites/Platforms	News Outlets/Media	Electronic Health Records Databases/Call records/ Online Surveys	Other Search Engines	Online Survey	Mobile App	N/A	Review
101	García-Díaz et al., 2018		✓													
102	Gayle et al., 2017		✓													
103	Genes et al., 2017		✓													
104	Gianfredi et al., 2018	✓														
105	Gianfredi et al., 2018															✓
106	Giat & Yom-Tov, 2018											✓				
107	Gittelman et al., 2015			✓												
108	Gohil et al., 2018															✓
109	Gough et al., 2017		✓													
110	Grajales et al., 2014	✓	✓				✓	✓	✓			✓				
111	Greaves et al., 2013								✓							
112	Griffis et al., 2014		✓	✓			✓									
113	Gruzd & Haythornthwaite 2013		✓													
114	Gu et al., 2014							✓				✓				
115	Guy et al., 2012															✓
116	Hamad et al., 2016														✓	
117	Hammer, 2017														✓	
118	Hand et al., 2016		✓													
119	Hanson et al., 2013		✓													
120	Hanson et al., 2013		✓													
121	Harris et al., 2014		✓													
122	Harris et al., 2014		✓													
123	Hébert et al., 2017	✓	✓						✓							
124	Hendriks et al., 2018			✓	✓											
125	Hill et al., 2011	✓							✓							
126	Hingle et al., 2013		✓													
127	Hswen et al., 2018		✓													
128	Huang et al., 2018									✓						
129	Huesch et al., 2017			✓												
130	Jankowski & Hofffmann, 2016	✓														
131	Jones et al., 2018							✓								
132	Jung et al., 2015							✓								
133	Jung et al., 2017								✓							
134	Kadry et al., 2011	✓							✓							
135	Kagashe et al., 2017		✓													

	Authors	Google	Twitter	Facebook	Instagram	Wikipedia	Other Social Media	Blogs/Forums/Communities	Websites/Platforms	News Outlets/Media	Electronic Health Records Databases/Call records/ Online Surveys	Other Search Engines	Online Survey	Mobile App	N/A	Review
136	Kalf et al., 2018															✓
137	Kandadai et al., 2016		✓													
138	Kandula et al., 2017	✓														
139	Katsuki et al., 2015		✓													
140	Keller et al., 2018			✓					✓							
141	Keller et al., 2017			✓												
142	Kendra et al., 2015		✓													
143	Khan et al., 2012		✓													
144	Khoury et al., 2012														✓	
145	Kim et al., 2015		✓													
146	Kim et al., 2014							✓								
147	Kim et al., 2017															✓
148	Kim et al., 2016		✓													
149	Kim et al., 2017		✓													
150	Klembczyk et al., 2016	✓														
151	Koh et al., 2014							✓								
152	Konheim-Kalkstein et al., 2018							✓								
153	Koschack et al., 2015							✓								
154	Kostkova et al., 2013	✓							✓	✓						
155	Krueger & Young, 2015		✓													
156	Kurzinger et al., 2018							✓								
157	Lachmar et al., 2017		✓													
158	Lama et al., 2018		✓													
159	Lardon et al., 2015															✓
160	Lau et al., 2011															✓
161	Lavorgna et al., 2018															✓
162	Lazard et al., 2016		✓													
163	Leal Neto et al., 2017													✓		
164	Lee et al., 2014		✓													
165	Lee et al., 2016	✓														
166	Lenoir et al., 2017		✓													
167	Leung et al., 2018								✓							
168	Li et al., 2018								✓							
169	Liang & Scammon, 2013	✓														
170	Lienemann et al., 2017															✓

	Authors	Google	Twitter	Facebook	Instagram	Wikipedia	Other Social Media	Blogs/Forums/Communities	Websites/Platforms	News Outlets/Media	Electronic Health Records Databases/Call records/ Online Surveys	Other Search Engines	Online Survey	Mobile App	N/A	Review
171	Ling & Lee, 2016	✓														
172	Liu et al., 2010								✓							
173	Liu et al., 2016		✓													
174	Liu et al., 2017											✓				
175	Liu et al., 2017		✓													
176	Liu et al., 2018		✓													
177	Livelo & Cheng, 2018		✓													
178	Lu et al., 2018	✓	✓						✓		✓					
179	Lyles et al., 2016		✓													
180	Mackey & Liang, 2013	✓	✓	✓			✓									
181	Mackey et al., 2018		✓													
182	Madden et al., 2017	✓														
183	Mahoney et al., 2015		✓							✓						
184	Mahroum et al. ,2018	✓														
185	Majumder et al., 2016	✓							✓							
186	Manchaiah et al., 2018		✓							✓						
187	Mao et al., 2014								✓							
188	Marcon et al., 2016		✓													
189	Marcus et al., 2012							✓								
190	Martinez et al., 2017													✓		
191	Martinez-Arroyo et al., 2018	✓														
192	Martinez-Millana et al., 2017	✓	✓	✓			✓									
193	Martins-Filho et al., 2018	✓														
194	Massey et al., 2016		✓													
195	Matsuda et al., 2017							✓								
196	Mavragani & Ochoa, 2018	✓														
197	Mavragani & Ochoa, 2018	✓														
198	Mavragani et al., 2018	✓														
199	Mavragani et al., 2018	✓														
200	Mazzocut et al., 2016								✓							
201	McNaughton et al., 2014							✓								
202	Meaney et al., 2016		✓													
203	Mejova et al., 2018			✓												
204	Melver et al., 2015		✓													
205	Menachemi et al., 2017											✓				

	Authors	Google	Twitter	Facebook	Instagram	Wikipedia	Other Social Media	Blogs/Forums/Communities	Websites/Platforms	News Outlets/Media	Electronic Health Records Databases/Call records/ Online Surveys	Other Search Engines	Online Survey	Mobile App	N/A	Review
206	Mendiola, 2015														✓	
207	Metwally et al., 2017		✓													
208	Miller et al., 2017		✓													
209	Mishori et al., 2014		✓													
210	Mnadla et al., 2016	✓														
211	Moccia et al., 2018															✓
212	Mollema et al., 2015		✓	✓				✓	✓	✓						
213	Mowery et al., 2017		✓													
214	Mukhija et al., 2017	✓														
215	Muralidhara & Paul, 2018				✓											
216	Myslin et al., 2013		✓													
217	Nagar et al., 2014	✓	✓													
218	Nagel et al., 2013		✓													
219	Nakada et al., 2014								✓	✓						
220	Nakhasi et al., 2014	✓							✓							
221	Nascimento et al., 2014		✓													
222	Nguyen et al., 2016		✓													
223	Nishimoto et al., 2016		✓													
224	Noll-Hussong, 2017	✓														
225	Nsoesie et al., 2014	✓							✓							
226	Odlum et al., 2018		✓													
227	Oldroyd et al., 2018															✓
228	Oser et al., 2017							✓								
229	Ozan-Rafferty, 2014	✓						✓								
230	Pan et al., 2018														✓	
231	Park & Hong, 2018								✓							
232	Peiper et al., 2017		✓													
233	Pervaiz et al., 2012	✓														
234	Pesala et al., 2017										✓					
235	Pesala et al., 2017										✓					
236	Phillips et al., 2018	✓														
237	Poirier et al., 2018	✓									✓					
238	Pretorius et al., 2018		✓													
239	Priest et al., 2016								✓							
240	Rabarison et al., 2017		✓													

	Authors	Google	Twitter	Facebook	Instagram	Wikipedia	Other Social Media	Blogs/Forums/Communities	Websites/Platforms	News Outlets/Media	Electronic Health Records Databases/Call records/ Online Surveys	Other Search Engines	Online Survey	Mobile App	N/A	Review
241	Radin & Sciascia, 2017	✓														
242	Radzikowski et al., 2016		✓													
243	Ragestar-Mojarad et al., 2016								✓							
244	Rastegar-Mojarad et al., 2015							✓								
245	Ricard et al., 2018				✓				✓							
246	Risson et al., 2016		✓	✓				✓								
247	Robillard et al., 2013							✓								
248	Roccetti et al., 2017			✓												
249	Rocheleau et al., 2015		✓													
250	Roberts et al., 2015		✓													
251	Rose et al., 2017		✓													
252	Rosenblum & Yom-Tov, 2017							✓				✓				
253	Sadah et al., 2015	✓	✓					✓	✓							
254	Sadah et al., 2016	✓	✓					✓	✓							
255	Saha et al., 2017	✓		✓												
256	Samaras et al., 2017	✓														
257	Santos & Matos, 2014		✓													
258	Sanz-Lorente et al., 2018		✓	✓	✓		✓									
259	Sarker et al., 2017		✓													
260	Sato et al., 2015							✓								
261	Schlichthorst et al., 2018		✓													
262	Sciascia & Radin, 2017	✓							✓							
263	Sciascia et al., 2018	✓							✓							
264	Seabrook et al., 2018		✓	✓										✓		
265	Seidl et al., 2018	✓														
266	Sentana-Lledo et al., 2016	✓														
267	Seo et al., 2014											✓				
268	Sewalk et al., 2018		✓													
269	SeyyedHosseini et al., 2018	✓							✓							
270	SeyyedHosseini et al., 2017														✓	
271	SeyyedHosseini et al., 2017	✓							✓							
272	Sharpe et al., 2016	✓	✓			✓										
273	Shi & Salmon, 2018						✓									
274	Simpson et al., 2018		✓													
275	Sinha et al., 2018		✓	✓												

	Authors	Google	Twitter	Facebook	Instagram	Wikipedia	Other Social Media	Blogs/Forums/Communities	Websites/Platforms	News Outlets/Media	Electronic Health Records Databases/Call records/ Online Surveys	Other Search Engines	Online Survey	Mobile App	N/A	Review
276	Sinnenberg et al., 2018		✓													
277	Smith et al., 2017			✓							✓					
278	Spyropoulos et al., 2018	✓													✓	
279	Staal et al., 2018								✓							
280	Stefanidis et al., 2017		✓													
281	Sudau et al., 2014							✓								
282	Sueki, 2015		✓										✓			
283	Sugawara et al., 2016		✓													
284	Sugawara et al., 2017		✓						✓	✓						
285	Surian et al., 2016		✓													
286	Tafti et al., 2017							✓	✓							
287	Tana et al., 2018	✓														
288	Tangherlini et al., 2016								✓							
289	Tapi Nzali et al., 2017			✓				✓								
290	Thackeray et al., 2013		✓													
291	Tighe et al., 2015		✓													
292	Timpka et al., 2014	✓							✓		✓					
293	Tinschert et al., 2017													✓		
294	Tougas et al., 2018		✓	✓	✓											
295	Triemstra et al., 2018		✓	✓	✓				✓							
296	Troullos et al., 2014												✓			
297	Tsuya et al., 2014		✓													
298	Tufts et al., 2018		✓													
299	Tyrawski & DeAndrea, 2015		✓				✓									
300	Utengen et al., 2017		✓													
301	van Lent et al., 2017		✓							✓						
302	Vasconcellos-Silva et al., 2017	✓														
303	Vickey & Breslin, 2017		✓													
304	Wagner et al., 2017		✓													
305	Wakamiya, 2018		✓													
306	Wang & Chen, 2018	✓														
307	Wang et al., 2015	✓														
308	Weeg et al., 2015		✓													
309	Williams et al., 2013															✓
310	Winchester et al., 2017	✓	✓				✓									

	Authors	Google	Twitter	Facebook	Instagram	Wikipedia	Other Social Media	Blogs/Forums/Communities	Websites/Platforms	News Outlets/Media	Electronic Health Records Databases/Call records/ Online Surveys	Other Search Engines	Online Survey	Mobile App	N/A	Review
311	Wittmeier et al., 2014	✓	✓	✓				✓								
312	Wong et al., 2013								✓							
313	Wong et al., 2015		✓													
314	Wongkoblap et al., 2017															✓
315	Woo et al., 2016											✓				
316	Wood et al., 2018	✓														
317	Xu & Liu, 2015													✓		
318	Xu et al., 2018			✓												
319	Xu et al., 2016		✓													
320	Yagahara et al., 2018		✓													
321	Yang et al., 2017											✓				
322	Yin et al., 2015		✓													
323	Yom-Tov & Gabrilovich, 2013											✓				
324	Yom-Tov et al., 2014											✓				
325	Yom-Tov et al., 2015					✓						✓				
326	Yom-Tov et al., 2014		✓									✓				
327	Yom-Tov & Lev-Ran, 2017											✓				
328	Young, 2018														✓	
329	Zeraatkar & Ahmadi, 2018															✓
330	Zhan et al., 2017		✓						✓							
331	Zhang et al., 2016	✓														
332	Zhang et al., 2018				✓											
333	Zhang et al., 2014													✓		
334	Zhang et al., 2013		✓													
335	Zhao & Yang, 2018							✓								
336	Zheluk et al., 2012	✓										✓				
337	Zheluk et al., 2013	✓										✓				
338	Zheluk et al., 2014	✓										✓				

References

1. Abbate KJ, Hingle MD, Armin J, Giacobbi P Jr, Gordon J. Recruiting Women to a Mobile Health Smoking Cessation Trial: Low- and No-Cost Strategies. JMIR Res Protoc 2017;6(11):e219. PMID: 29101091
2. Abbe A, Falissard B. Stopping Antidepressants and Anxiolytics as Major Concerns Reported in Online Health Communities: A Text Mining Approach. JMIR Ment Health 2017;4(4):e48. PMID: 29061554
3. Abdellaoui R, Schück S, Texier N, Burgun A. Filtering Entities to Optimize Identification of Adverse Drug Reaction From Social Media: How Can the Number of Words Between Entities in the Messages Help? JMIR Public Health Surveill 2017;3(2):e36. PMID: 28642212
4. Abdellaoui R, Foulquia P, Texier N, Faviez C, Burgun A, Schack S. Detection of Cases of Noncompliance to Drug Treatment in Patient Forum Posts: Topic Model Approach. J Med Internet Res. 2018 Mar 14;20(3):e85. PMID:29540337
5. Adams S. Post-Panoptic Surveillance Through Healthcare Rating Sites: Who's watching whom? Information Communication and Society. 2013;16(2):215-235 DOI: 10.1080/1369118X.2012.701657
6. Adawi M, Bragazzi NL, Watad A, Sharif K, Amital H, Mahroum N. Discrepancies Between Classic and Digital Epidemiology in Searching for the Mayaro Virus: Preliminary Qualitative and Quantitative Analysis of Google Trends. JMIR Public Health Surveill 2017;3(4):e93. PMID: 29196278
7. Adrover C, Bodnar T, Huang Z, Telenti A, Salathé M. Identifying Adverse Effects of HIV Drug Treatment and Associated Sentiments Using Twitter. JMIR Public Health Surveill 2015;1(2):e7. PMID: 27227141
8. Adusumalli S, Lee H, Hoi Q, Koo SL, Tan IB, Ng PC. Assessment of Web-Based Consumer Reviews as a Resource for Drug Performance. J Med Internet Res 2015;17(8):e211. PMID: 26319108
9. Agarwal V, Zhang L, Zhu J, Fang S, Cheng T, Hong C, Shah NH. Impact of Predicting Health Care Utilization Via Web Search Behavior: A Data-Driven Analysis. J Med Internet Res 2016;18(9):e251. PMID: 27655225
10. Albalawi Y, Sixsmith J. Agenda Setting for Health Promotion: Exploring an Adapted Model for the Social Media Era. JMIR Public Health Surveill 2015;1(2):e21. PMID: 27227139
11. Allem JP, Escobedo P, Chu KH, Boley Cruz T, Unger JB. Images of Little Cigars and Cigarillos on Instagram Identified by the Hashtag #swisher: Thematic AnalysisJ Med Internet Res 2017;19(7):e255. PMID: 28710057
12. Allem JP, Dharmapuri L, Leventhal AM, Unger JB, Boley Cruz T. Hookah-Related Posts to Twitter From 2017 to 2018: Thematic Analysis. J Med Internet Res. 2018 Nov 19;20(11):e11669. PMID:30455162
13. Allem JP, Ferrara E, Uppu SP, Cruz TB, Unger JB. E-Cigarette Surveillance With Social Media Data: Social Bots, Emerging Topics, and Trends. JMIR Public Health Surveill. 2017 Dec 20;3(4):e98. doi: 10.2196/publichealth.8641. PMID:29263018
14. Allem JP, Ramanujam J, Lerman K, Chu KH, Boley Cruz T, Unger JB. Identifying Sentiment of Hookah-Related Posts on Twitter. JMIR Public Health Surveill 2017;3(4):e74. PMID: 29046267
15. Alnemer KA, Alhuzaim WM, Alnemer AA, Alharbi BB, Bawazir AS, Barayyan OR, Balaraj FK. Are Health-Related Tweets Evidence Based? Review and Analysis of Health-Related Tweets on Twitter. J Med Internet Res 2015;17(10):e246. PMID: 26515535.
16. Alvarez-Mon MA, Asunsolo del Barco A, Lahera G, Quintero J, Ferre F, Pereira-Sanchez V, Ortuño F, Alvarez-Mon M. Increasing Interest of Mass Communication Media and the General Public in the Distribution of Tweets About Mental Disorders: Observational Study. J Med Internet Res 2018;20(5):e205. PMID: 29807880
17. Alvaro N, Miyao Y, Collier N. TwiMed: Twitter and PubMed Comparable Corpus of Drugs, Diseases, Symptoms, and Their Relations. JMIR Public Health Surveill 2017;3(2):e24. PMID: 28468748
18. Anderson LS, Bell HG, Gilbert M, Davidson JE, Winter C, Barratt MJ, Win B, Painter JL, Menone C, Sayegh J, Dasgupta N. Using Social Listening Data to Monitor Misuse and Nonmedical Use of Bupropion: A Content Analysis. JMIR Public Health Surveill. 2017 Feb 1;3(1):e6. PMID:28148472

19. Aoki T, Suzuki T, Yagahara A, Hasegawa S, Tsuji S, Ogasawara K. Analysis of the Regionality of the Number of Tweets Related to the 2011 Fukushima Nuclear Power Station Disaster: Content Analysis. JMIR Public Health Surveill 2018;4(4):e70. PMID: 30563815
20. Arnhold M, Quade M, Kirch W. Mobile Applications for Diabetics: A Systematic Review and Expert-Based Usability Evaluation Considering the Special Requirements of Diabetes Patients Age 50 Years or Older. J Med Internet Res 2014;16(4):e104. PMID: 24718852
21. Aslam AA, Tsou MH, Spitzberg BH, An L, Gawron JM, Gupta DK, Peddecord KM, Nagel AC, Allen C, Yang JA, Lindsay S. The reliability of tweets as a supplementary method of seasonal influenza surveillance. J Med Internet Res. 2014 Nov 14;16(11):e250. doi: 10.2196/jmir.3532. PMID:25406040
22. Athilingam P, Jenkins B. Mobile Phone Apps to Support Heart Failure Self-Care Management: Integrative Review. JMIR Cardio 2018;2(1):e10057. DOI: 10.2196/10057
23. Ayers JW, Althouse BM, Allem JP, Ford DE, Ribisl KM, Cohen JE. A novel evaluation of World No Tobacco day in Latin America. J Med Internet Res. 2012 May 28;14(3):e77. doi: 10.2196/jmir.2148. PMID:22634568
24. Ayers JW, Westmaas JL, Leas EC, Benton A, Chen Y, Dredze M, Althouse BM. Leveraging Big Data to Improve Health Awareness Campaigns: A Novel Evaluation of the Great American Smokeout. JMIR Public Health Surveill. 2016 Mar 31;2(1):e16. PMID:27227151
25. Balls-Berry J, Sinicrope P, Valdez Soto M, Brockman T, Bock M, Patten C. Linking Podcasts With Social Media to Promote Community Health and Medical Research: Feasibility Study. JMIR Form Res 2018;2(2):e10025. PMID: 30684430
26. Baltrusaitis K, Santillana M, Crawley AW, Chunara R, Smolinski M, Brownstein JS. Determinants of Participants' Follow-Up and Characterization of Representativeness in Flu Near You, A Participatory Disease Surveillance System. JMIR Public Health Surveill 2017;3(2):e18. PMID: 28389417
27. Ben-Sasson A, Yom-Tov E. Online Concerns of Parents Suspecting Autism Spectrum Disorder in Their Child: Content Analysis of Signs and Automated Prediction of Risk. J Med Internet Res 2016;18(11):e300. PMID: 27876688
28. Berlinberg EJ, Deiner MS, Porco TC, Acharya NR. Monitoring Interest in Herpes Zoster Vaccination: Analysis of Google Search Data. JMIR Public Health Surveill. 2018 May 2;4(2):e10180. PMID:29720364
29. Bernardo TM, Rajic A, Young I, Robiadek K, Pham MT, Funk JA. Scoping Review on Search Queries and Social Media for Disease Surveillance: A Chronology of Innovation. J Med Internet Res 2013;15(7):e147. PMID: 23896182
30. Berry N, Lobban F, Belousov M, Emsley R, Nenadic G, Bucci S. #WhyWeTweetMH: Understanding Why People Use Twitter to Discuss Mental Health Problems. J Med Internet Res 2017;19(4):e107. PMID: 28381392
31. Bian J, Zhao Y, Salloum RG, Guo Y, Wang M, Prosperi M, Zhang H, Du X, Ramirez-Diaz LJ, He Z, Sun Y. Using Social Media Data to Understand the Impact of Promotional Information on Laypeople's Discussions: A Case Study of Lynch Syndrome. J Med Internet Res 2017;19(12):e414. PMID: 29237586
32. Birnbaum ML, Ernala SK, Rizvi AF, De Choudhury M, Kane JM. A Collaborative Approach to Identifying Social Media Markers of Schizophrenia by Employing Machine Learning and Clinical Appraisals. J Med Internet Res 2017;19(8):e289. PMID: 28807891
33. Bollegala D, Maskell S, Sloane R, Hajne J, Pirmohamed M. Causality Patterns for Detecting Adverse Drug Reactions From Social Media: Text Mining Approach. JMIR Public Health Surveill 2018;4(2):e51. PMID: 29743155
34. Bousquet C, Dahamna B, Guillemin-Lanne S, Darmoni SJ, Faviez C, Huot C, Katsahian S, Leroux V, Pereira S, Richard C, Schück S, Souvignet J, Lillo-Le Louët A, Texier N. The Adverse Drug Reactions from Patient Reports in Social Media Project: Five Major Challenges to Overcome to Operationalize Analysis and Efficiently Support Pharmacovigilance Process. JMIR Res Protoc 2017;6(9):e179. PMID: 28935617
35. Bragazzi NL. A Google Trends-based approach for monitoring NSSI. Psychol Res Behav Manag. 2013; 13;7:1-8. PMID:24376364

36. Bragazzi NL. Infodemiology and infoveillance of multiple sclerosis in Italy. Mult Scler Int. 2013;2013:924029. PMID:24027636
37. Bragazzi NL, Bacigaluppi S, Robba C, Nardone R, Trinka E, Brigo F. Infodemiology of status epilepticus: A systematic validation of the Google Trends-based search queries. Epilepsy Behav. 2016 Feb;55:120-3. doi: 10.1016/j.yebeh.2015.12.017. Epub 2016 Jan 13. PMID:26773681
38. Bragazzi NL, Bacigaluppi S, Robba C, Siri A, Canepa G, Brigo F. Infodemiological data of West-Nile virus disease in Italy in the study period 2004-2015. Data Brief. 2016 Nov 2;9:839-845. eCollection 2016 Dec. PMID:27872881
39. Bragazzi NL, Dini G, Toletone A, Brigo F, Durando P. Infodemiological data concerning silicosis in the USA in the period 2004-2010 correlating with real-world statistical data. Data Brief. 2016 Nov 13;10:457-464. PMID:28054008
40. Braithwaite SR, Giraud-Carrier C, West J, Barnes MD, Hanson CL. Validating Machine Learning Algorithms for Twitter Data Against Established Measures of Suicidality. JMIR Ment Health 2016;3(2):e21. PMID: 27185366
41. Brigo F, Erro R. Why do people google movement disorders? An infodemiological study of information seeking behaviors. Neurol Sci. 2016 May;37(5):781-7. PMID:26846327
42. Brigo F, Trinka E. Google search behavior for status epilepticus. Epilepsy Behav. 2015 Aug;49:146-9. doi: 10.1016/j.yebeh.2015.02.029. Epub 2015 Apr 11. PMID:25873438
43. Brigo F, Igwe SC, Ausserer H, Nardone R, Tezzon F, Bongiovanni LG, Tinazzi M, Trinka E. Terminology of psychogenic nonepileptic seizures. Epilepsia. 2015 Mar;56(3):e21-5. PMID:25631657
44. Brigo F, Igwe SC, Ausserer H, Nardone R, Tezzon F, Bongiovanni LG, Trinka E. Why do people Google epilepsy? An infodemiological study of online behavior for epilepsy related search terms. Epilepsy Behav. 2014 Feb;31:67-70. PMID:24361764
45. Brigo F, Igwe SC, Nardone R, Lochner P, Tezzon F, Otte WM. Wikipedia and neurological disorders. J Clin Neurosci. 2015 Jul;22(7):1170-2. PMID:25890773
46. Brigo F, Lattanzi S, Bragazzi N, Nardone R, Moccia M, Lavorgna L. Why do people search Wikipedia for information on multiple sclerosis? Mult Scler Relat Disord. 2018 Feb;20:210-214. PMID:29428464
47. Brigo F, Lattanzi S, Giussani G, Tassi L, Pietrafusa N, Galimberti CA, Nardone R, Bragazzi NL, Mecarelli O. Italian Wikipedia and epilepsy: An infodemiological study of online information-seeking behavior. Epilepsy Behav. 2018 Apr;81:119-122. PMID:29454607
48. Brigo F, Lochner P, Tezzon F, Nardone R. Web search behavior for multiple sclerosis: An infodemiological study. Mult Scler Relat Disord. 2014 Jul;3(4):440-3. PMID:25877054
49. Brigo F, Otte WM, Igwe SC, Ausserer H, Nardone R, Tezzon F, Trinka E. Information-seeking behaviour for epilepsy: an infodemiological study of searches for Wikipedia articles. Epileptic Disord. 2015 Dec;17(4):460-6. PMID:26575365
50. Brigo F., Igwe S.C., Nardone R., Orioli A., Otte W.M. Cancer information disparities on the internet: An infodemiological study. Journal of Cancer Policy. 2016;8:33-37 DOI: 10.1016/j.jcpo.2016.04.002
51. Broniatowski DA, Dredze M, Paul MJ, Dugas A. Using Social Media to Perform Local Influenza Surveillance in an Inner-City Hospital: A Retrospective Observational Study. JMIR Public Health Surveill 2015;1(1):e5. PMID: 27014744
52. Bubenzer R.H. Infodemiology as shown by influenza: New opportunities of the Internet [Infodemiologie am beispiel influenza: Die neuen chancen des Internets] Klinikarzt. 2009:38(2):62 DOI: 10.1055/s-0029-1214174
53. Burton SH, Tanner KW, Giraud-Carrier CG, West JH, Barnes MD. Right time, right place" health communication on Twitter: value and accuracy of location information. J Med Internet Res. 2012 Nov 15;14(6):e156. PMID:23154246
54. Callahan A, Pernek I, Stiglic G, Leskovec J, Strasberg HR, Shah NH. Analyzing Information Seeking and Drug-Safety Alert Response by Health Care Professionals as New Methods for Surveillance. J Med Internet Res 2015;17(8):e204. PMID: 26293444

55. Carrotte ER, Prichard I, Lim MSC. “Fitspiration” on Social Media: A Content Analysis of Gendered Images. J Med Internet Res 2017;19(3):e95. PMID: 28356239
56. Cartwright AF, Karunaratne M, Barr-Walker J, Johns NE, Upadhyay UD. Identifying National Availability of Abortion Care and Distance From Major US Cities: Systematic Online Search. J Med Internet Res 2018;20(5):e186. PMID: 29759954
57. Cavazos-Rehg P, Krauss M, Grucza R, Bierut L. Characterizing the Followers and Tweets of a Marijuana-Focused Twitter Handle. J Med Internet Res 2014;16(6):e157. PMID: 24974893
58. Cawkwell PB, Lee L, Weitzman M, Sherman SE. Tracking Hookah Bars in New York: Utilizing Yelp as a Powerful Public Health Tool. JMIR Public Health Surveill 2015;1(2):e19. PMID: 27227137
59. Chan K, Ho S, Lam T. Infodemiology of alcohol use in Hong Kong mentioned on blogs: infoveillance study. J Med Internet Res. 2013 Sep 2;15(9):e192. PMID:23999327
60. Chen T, Dredze M. Vaccine Images on Twitter: Analysis of What Images are Shared. J Med Internet Res 2018;20(4):e130. PMID: 29615386
61. Chen L, Wang X, Peng T. Nature and Diffusion of Gynecologic Cancer-Related Misinformation on Social Media: Analysis of Tweets. J Med Internet Res. ***2018;20(10):e11515.*** PMID: 30327289
62. Chen B, Shao J, Liu K, Cai G, Jiang Z, Huang Y, Gu H, Jiang J. Does Eating Chicken Feet With Pickled Peppers Cause Avian Influenza? Observational Case Study on Chinese Social Media During the Avian Influenza A (H7N9) Outbreak. JMIR Public Health Surveill. 2018 Mar 29;4(1):e32. PMID:29599109
63. Chen S, Xu Q, Buchenberger J, Bagavathi A, Fair G, Shaikh S, Krishnan S. Dynamics of Health Agency Response and Public Engagement in Public Health Emergency: A Case Study of CDC Tweeting Patterns During the 2016 Zika Epidemic. JMIR Public Health Surveill. 2018 Nov 22;4(4):e10827. doi: 10.2196/10827. PMID:30467106
64. Chen AT, Zhu SH, Conway M. What Online Communities Can Tell Us About Electronic Cigarettes and Hookah Use: A Study Using Text Mining and Visualization Techniques. J Med Internet Res 2015;17(9):e220. PMID: 26420469
65. Cheng Q, Li TM, Kwok CL, Zhu T, Yip PS. Assessing Suicide Risk and Emotional Distress in Chinese Social Media: A Text Mining and Machine Learning Study. J Med Internet Res 2017;19(7):e243. PMID: 28694239
66. Cheng TYM, Liu L, Woo BK. Analyzing Twitter as a Platform for Alzheimer-Related Dementia Awareness: Thematic Analyses of Tweets. JMIR Aging 2018;1(2):e11542. DOI: 10.2196/11542
67. Cherian R, Westbrook M, Ramo D, Sarkar U. Representations of Codeine Misuse on Instagram: Content Analysis. JMIR Public Health Surveill 2018;4(1):e22. PMID: 29559422
68. Chew C, Eysenbach G. Pandemics in the age of Twitter: content analysis of Tweets during the 2009 H1N1 outbreak. PLoS One. 2010 Nov 29;5(11):e14118. PMID:21124761
69. Chomutare T, Fernandez-Luque L, Årsand E, Hartvigsen G. Features of Mobile Diabetes Applications: Review of the Literature and Analysis of Current Applications Compared Against Evidence-Based Guidelines. J Med Internet Res 2011;13(3):e65. PMID: 21979293
70. Christmann CA, Hoffmann A, Bleser G. Stress Management Apps With Regard to Emotion-Focused Coping and Behavior Change Techniques: A Content Analysis. JMIR Mhealth Uhealth 2017;5(2):e22. PMID: 28232299
71. Chu KH, Sidhu AK, Valente TW. Electronic Cigarette Marketing Online: a Multi-Site, Multi-Product Comparison. JMIR Public Health Surveill 2015;1(2):e11. PMID: 27227129
72. Clyne W, Pezaro S, Deeny K, Kneafsey R. Using Social Media to Generate and Collect Primary Data: The #ShowsWorkplaceCompassion Twitter Research Campaign. JMIR Public Health Surveill 2018;4(2):e41. PMID: 29685866
73. Colditz JB, Chu KH, Emery SL, Larkin CR, James AE, Welling J, Primack BA. Toward Real-Time Infoveillance of Twitter Health Messages. Am J Public Health. 2018 Aug;108(8):1009-1014. PMID:29927648
74. Cole-Lewis H, Pugatch J, Sanders A, Varghese A, Posada S, Yun C, Schwarz M, Augustson E. Social Listening: A Content Analysis of E-Cigarette Discussions on Twitter. J Med Internet Res 2015;17(10):e243. PMID: 26508089

75. Cole-Lewis H, Varghese A, Sanders A, Schwarz M, Pugatch J, Augustson E. Assessing Electronic Cigarette-Related Tweets for Sentiment and Content Using Supervised Machine Learning. J Med Internet Res 2015;17(8):e208. PMID: 26307512
76. Conway M. Ethical Issues in Using Twitter for Public Health Surveillance and Research: Developing a Taxonomy of Ethical Concepts From the Research Literature. J Med Internet Res 2014;16(12):e290. PMID: 25533619
77. Cortés V.D., Velásquez J.D., Ibáñez C.F. Twitter for marijuana infodemiology. Proceedings - 2017 IEEE/WIC/ACM International Conference on Web Intelligence, WI 2017: 730-736 DOI: 10.1145/3106426.3106541
78. Daniulaityte R, Chen L, Lamy FR, Carlson RG, Thirunarayan K, Sheth A. "When 'Bad' is 'Good'": Identifying Personal Communication and Sentiment in Drug-Related Tweets. JMIR Public Health Surveill 2016;2(2):e162. PMID: 27777215
79. Davis MA, Zheng K, Liu Y, Levy H. Public Response to Obamacare on Twitter. J Med Internet Res 2017;19(5):e167. PMID: 28550002
80. de Viron S, Suggs LS, Brand A, Van Oyen H. Communicating Genetics and Smoking Through Social Media: Are We There Yet?. J Med Internet Res 2013;15(9):e198. PMID: 24018012
81. DeJohn AD, Schulz EE, Pearson AL, Lachmar EM, Wittenborn AK. Identifying and Understanding Communities Using Twitter to Connect About Depression: Cross-Sectional Study. JMIR Ment Health 2018;5(4):e61. PMID: 30401662
82. Delaney KP, Kramer MR, Waller LA, Flanders WD, Sullivan PS. Using a Geolocation Social Networking Application to Calculate the Population Density of Sex-Seeking Gay Men for Research and Prevention Services. J Med Internet Res 2014;16(11):e249. PMID: 25406722
83. Delir Haghighi P, Kang YB, Buchbinder R, Burstein F, Whittle S. Investigating Subjective Experience and the Influence of Weather Among Individuals With Fibromyalgia: A Content Analysis of Twitter. JMIR Public Health Surveill. 2017 Jan 19;3(1):e4. PMID:28104577
84. Doan S, Ritchart A, Perry N, Chaparro JD, Conway M. How Do You #relax When You're #stressed? A Content Analysis and Infodemiology Study of Stress-Related Tweets. JMIR Public Health Surveill. 2017 Jun 13;3(2):e35. PMID:28611016
85. Domnich A., Arbuzova E.K., Signori A., Amicizia D., Panatto D., Gasparini R. Demand-based web surveillance of sexually transmitted infections in Russia. International Journal of Public Health. 2014;59(5):841-849 DOI: 10.1007/s00038-014-0581-7
86. Du L, Rachul C, Guo Z, Caulfield T. Gordie Howe's "Miraculous Treatment": Case Study of Twitter Users' Reactions to a Sport Celebrity's Stem Cell Treatment. JMIR Public Health Surveill. 2016 Mar 9;2(1):e8. PMID:27227162
87. Du J, Tang L, Xiang Y, Zhi D, Xu J, Song HY, Tao C. Public Perception Analysis of Tweets During the 2015 Measles Outbreak: Comparative Study Using Convolutional Neural Network Models. J Med Internet Res 2018;20(7):e236. PMID: 29986843
88. Duke JC, Hansen H, Kim AE, Curry L, Allen J. The Use of Social Media by State Tobacco Control Programs to Promote Smoking Cessation: A Cross-Sectional Study. J Med Internet Res 2014;16(7):e169. PMID: 25014311
89. Dunn AG, Leask J, Zhou X, Mandl KD, Coiera E. Associations Between Exposure to and Expression of Negative Opinions About Human Papillomavirus Vaccines on Social Media: An Observational Study. J Med Internet Res 2015;17(6):e144. PMID: 26063290
90. Dyson MP, Newton AS, Shave K, Featherstone RM, Thomson D, Wingert A, Fernandes RM, Hartling L. Social Media for the Dissemination of Cochrane Child Health Evidence: Evaluation Study. J Med Internet Res 2017;19(9):e308. PMID: 28864427
91. Edney S, Bogomolova S, Ryan J, Olds T, Sanders I, Maher C. Creating Engaging Health Promotion Campaigns on Social Media: Observations and Lessons From Fitbit and Garmin. J Med Internet Res 2018;20(12):e10911. PMID: 30530449

92. Eklund A.-M. Tracking changes in search behaviour at a health web site. Studies in Health Technology and Informatics. 2012; 180:858-862. DOI: 10.3233/978-1-61499-101-4-858
93. Espina K., Estuar Ma.R.J.E. Infodemiology for Syndromic Surveillance of Dengue and Typhoid Fever in the Philippines. Procedia Computer Science. 2017;121:554-561. DOI:10.1016/j.procs.2017.11.073
94. Espina K., Justina Estuar M.R., Ix D.J.S., Lara R.J.E., De Los Reyes V.C. Towards an Infodemiological Algorithm for Classification of Filipino Health Tweets. Procedia Computer Science 2016;100:686-692. DOI: 10.1016/j.procs.2016.09.212
95. Eysenbach G. Infodemiology and infoveillance tracking online health information and cyberbehavior for public health. Am J Prev Med. 2011 May;40(5 Suppl 2):S154-8. PMID:21521589
96. Eysenbach G. Infodemiology and infoveillance: framework for an emerging set of public health informatics methods to analyze search, communication and publication behavior on the Internet. J Med Internet Res. 2009 Mar 27;11(1):e11. PMID:19329408
97. Farhadloo M, Winneg K, Chan MPS, Hall Jamieson K, Albarracin D. Associations of Topics of Discussion on Twitter With Survey Measures of Attitudes, Knowledge, and Behaviors Related to Zika: Probabilistic Study in the United States. JMIR Public Health Surveill 2018;4(1):e16. PMID: 29426815
98. Foroughi F, Lam AK, Lim MSC, Saremi N, Ahmadvand A. Googling" for Cancer: An Infodemiological Assessment of Online Search Interests in Australia, Canada, New Zealand, the United Kingdom, and the United States. JMIR Cancer. 2016 May 4;2(1):e5. PMID:28410185
99. Gabarron E, Serrano JA, Wynn R, Lau AY. Tweet Content Related to Sexually Transmitted Diseases: No Joking Matter. J Med Internet Res 2014;16(10):e228. PMID: 25289463
100. Gabarron E, Lau AY, Wynn R. Is There a Weekly Pattern for Health Searches on Wikipedia and Is the Pattern Unique to Health Topics?. J Med Internet Res 2015;17(12):e286. PMID: 26693859
101. García-Díaz J.A., Apolinario-Arzube O., Medina-Moreira J., Salavarria-Melo J.O., Lagos-Ortiz K., Luna-Aveiga H., Valencia-García R. Opinion mining for measuring the social perception of infectious diseases. An infodemiology approach Communications in Computer and Information Science 2018;883:229-239. DOI: 10.1007/978-3-030-00940-3_17
102. Gayle A, Shimaoka M. Public Response to Scientific Misconduct: Assessing Changes in Public Sentiment Toward the Stimulus-Triggered Acquisition of Pluripotency (STAP) Cell Case via Twitter. JMIR Public Health Surveill 2017;3(2):e21. PMID: 28428163
103. Genes N, Chary M, Chason K. Analysis of Twitter Users' Sharing of Official New York Storm Response Messages. Med 2 0 2014;3(1):e1. PMID: 25075245
104. Gianfredi V, Bragazzi NL, Mahamid M, Bisharat B, Mahroum N, Amital H, Adawi M. Monitoring public interest toward pertussis outbreaks: an extensive Google Trends-based analysis. Public Health. 2018 Dec;165:9-15. PMID:30342281
105. Gianfredi V, Bragazzi NL, Nucci D, Martini M, Rosselli R, Minelli L, Moretti M. Harnessing Big Data for Communicable Tropical and Sub-Tropical Disorders: Implications From a Systematic Review of the Literature. Front Public Health. 2018 Mar 21;6:90. eCollection 2018. PMID:29619364
106. Giat E, Yom-Tov E. Evidence From Web-Based Dietary Search Patterns to the Role of B12 Deficiency in Non-Specific Chronic Pain: A Large-Scale Observational Study. J Med Internet Res 2018;20(1):e4. PMID: 29305340
107. Gittelman S, Lange V, Gotway Crawford CA, Okoro CA, Lieb E, Dhingra SS, Trimarchi E. A New Source of Data for Public Health Surveillance: Facebook Likes. J Med Internet Res 2015;17(4):e98. PMID: 25895907
108. Gohil S, Vuik S, Darzi A. Sentiment Analysis of Health Care Tweets: Review of the Methods Used. JMIR Public Health Surveill 2018;4(2):e43. PMID: 29685871
109. Gough A, Hunter RF, Ajao O, Jurek A, McKeown G, Hong J, Barrett E, Ferguson M, McElwee G, McCarthy M, Kee F. Tweet for Behavior Change: Using Social Media for the Dissemination of Public Health Messages. JMIR Public Health Surveill 2017;3(1):e14. PMID: 28336503
110. Grajales FJ III, Sheps S, Ho K, Novak-Lauscher H, Eysenbach G. Social Media: A Review and Tutorial of Applications in Medicine and Health Care. J Med Internet Res 2014;16(2):e13. PMID: 24518354

111. Greaves F, Ramirez-Cano D, Millett C, Darzi A, Donaldson L. Use of Sentiment Analysis for Capturing Patient Experience From Free-Text Comments Posted Online. J Med Internet Res 2013;15(11):e239. PMID: 24184993
112. Griffis HM, Kilaru AS, Werner RM, Asch DA, Hershey JC, Hill S, Ha YP, Sellers A, Mahoney K, Merchant RM. Use of Social Media Across US Hospitals: Descriptive Analysis of Adoption and Utilization. J Med Internet Res 2014;16(11):e264. PMID: 25431831
113. Gruzd A, Haythornthwaite C. Enabling Community Through Social Media. J Med Internet Res 2013;15(10):e248. PMID: 24176835
114. Gu H, Chen B, Zhu H, Jiang T, Wang X, Chen L, Jiang Z, Zheng D, Jiang J. Importance of Internet Surveillance in Public Health Emergency Control and Prevention: Evidence From a Digital Epidemiologic Study During Avian Influenza A H7N9 Outbreaks. J Med Internet Res 2014;16(1):e20. PMID: 24440770
115. Guy S., Ratzki-Leewing A., Bahati R., Gwadry-Sridhar F. Social media: A systematic review to understand the evidence and application in infodemiology. Lecture Notes of the Institute for Computer Sciences, Social-Informatics and Telecommunications Engineering 2012;1-8. DOI: 10.1007/978-3-642-29262-0_1
116. Hamad EO, Savundranayagam MY, Holmes JD, Kinsella EA, Johnson AM. Toward a Mixed-Methods Research Approach to Content Analysis in The Digital Age: The Combined Content-Analysis Model and its Applications to Health Care Twitter Feeds. J Med Internet Res. 2016 Mar 8;18(3):e60. PMID:26957477
117. Hammer MJ. Ethical Considerations When Using Social Media for Research. Oncol Nurs Forum. 2017;44(4):410-412. PMID:28632249
118. Hand RK, Kenne D, Wolfram TM, Abram JK, Fleming M. Assessing the Viability of Social Media for Disseminating Evidence-Based Nutrition Practice Guideline Through Content Analysis of Twitter Messages and Health Professional Interviews: An Observational Study. J Med Internet Res 2016;18(11):e295. PMID: 27847349
119. Hanson CL, Burton SH, Giraud-Carrier C, West JH, Barnes MD, Hansen B. Tweaking and Tweeting: Exploring Twitter for Nonmedical Use of a Psychostimulant Drug (Adderall) Among College Students. J Med Internet Res 2013;15(4):e62. PMID: 23594933
120. Hanson CL, Cannon B, Burton S, Giraud-Carrier C. An Exploration of Social Circles and Prescription Drug Abuse Through Twitter. J Med Internet Res 2013;15(9):e189. PMID: 24014109
121. Harris JK, Choucair B, Maier RC, Jolani N, Bernhardt JM. Are Public Health Organizations Tweeting to the Choir? Understanding Local Health Department Twitter Followership. J Med Internet Res 2014;16(2):e31. PMID: 24571914
122. Harris JK, Moreland-Russell S, Choucair B, Mansour R, Staub M, Simmons K. Tweeting for and Against Public Health Policy: Response to the Chicago Department of Public Health's Electronic Cigarette Twitter Campaign. J Med Internet Res 2014;16(10):e238. PMID: 25320863
123. Hébert J, Robitaille H, Turcotte S, Légaré F. Online Dissemination Strategies of a Canada Research Chair: Overview and Lessons Learned. JMIR Res Protoc 2017;6(2):e27. PMID: 28235751
124. Hendriks H, Van den Putte B, Gebhardt WA, Moreno MA. Social Drinking on Social Media: Content Analysis of the Social Aspects of Alcohol-Related Posts on Facebook and Instagram. J Med Internet Res 2018;20(6):e226. PMID: 29934290
125. Hill S, Mao J, Ungar L, Hennessy S, Leonard CE, Holmes J. Natural supplements for H1N1 influenza: retrospective observational infodemiology study of information and search activity on the Internet. J Med Internet Res. 2011 May 10;13(2):e36. PMID:21558062
126. Hingle M, Yoon D, Fowler J, Kobourov S, Schneider ML, Falk D, Burd R. Collection and Visualization of Dietary Behavior and Reasons for Eating Using Twitter. J Med Internet Res 2013;15(6):e125. PMID: 23796439
127. Hswen Y, Naslund JA, Brownstein JS, Hawkins JB. Monitoring Online Discussions About Suicide Among Twitter Users With Schizophrenia: Exploratory Study. JMIR Ment Health 2018;5(4):e11483. PMID: 30545811

128. Huang M, ElTayeby O, Zolnoori M, Yao L. Public Opinions Toward Diseases: Infodemiological Study on News Media Data. J Med Internet Res 2018;20(5):e10047. PMID: 29739741
129. Huesch M, Chetlen A, Segel J, Schetter S. Frequencies of Private Mentions and Sharing of Mammography and Breast Cancer Terms on Facebook: A Pilot Study. J Med Internet Res 2017;19(6):e201. PMID: 28600279
130. Jankowski W, Hoffmann M. Can Google Searches Predict the Popularity and Harm of Psychoactive Agents?. J Med Internet Res 2016;18(2):e38. PMID: 26916984
131. Jones J, Pradhan M, Hosseini M, Kulanthaivel A, Hosseini M. Novel Approach to Cluster Patient-Generated Data Into Actionable Topics: Case Study of a Web-Based Breast Cancer Forum. JMIR Med Inform. 2018 Nov 29;6(4):e45. PMID:30497991
132. Jung Y, Hur C, Jung D, Kim M. Identifying Key Hospital Service Quality Factors in Online Health Communities. J Med Internet Res 2015;17(4):e90. PMID: 25855612
133. Jung H, Park HA, Song TM. Ontology-Based Approach to Social Data Sentiment Analysis: Detection of Adolescent Depression Signals. J Med Internet Res 2017;19(7):e259. PMID: 28739560
134. Kadry B, Chu LF, Kadry B, Gammas D, Macario A. Analysis of 4999 Online Physician Ratings Indicates That Most Patients Give Physicians a Favorable Rating. J Med Internet Res 2011;13(4):e95. PMID: 22088924
135. Kagashe I, Yan Z, Suheryani I. Enhancing Seasonal Influenza Surveillance: Topic Analysis of Widely Used Medicinal Drugs Using Twitter Data. J Med Internet Res 2017;19(9):e315. PMID: 28899847.
136. Kalf RR, Makady A, ten Ham RM, Meijboom K, Goettsch WG, On Behalf Of IMI-GetReal Workpackage 1. Use of Social Media in the Assessment of Relative Effectiveness: Explorative Review With Examples From Oncology. JMIR Cancer 2018;4(1):e11. PMID: 29884607
137. Kandadai V, Yang H, Jiang L, Yang CC, Fleisher L, Winston FK. Measuring Health Information Dissemination and Identifying Target Interest Communities on Twitter: Methods Development and Case Study of the @SafetyMD Network. JMIR Res Protoc 2016;5(2):e50. PMID: 27151100
138. Kandula S, Hsu D, Shaman J. Subregional Nowcasts of Seasonal Influenza Using Search Trends. J Med Internet Res. 2017 Nov 6;19(11):e370. PMID:29109069
139. Katsuki T, Mackey TK, Cuomo R. Establishing a Link Between Prescription Drug Abuse and Illicit Online Pharmacies: Analysis of Twitter Data. J Med Internet Res. 2015 Dec 16;17(12):e280. PMID:26677966
140. Keller MS, Mosadeghi S, Cohen ER, Kwan J, Spiegel BMR. Reproductive Health and Medication Concerns for Patients With Inflammatory Bowel Disease: Thematic and Quantitative Analysis Using Social Listening. J Med Internet Res. 2018 Jun 11;20(6):e206. PMID:29891471
141. Keller MS, Park HJ, Cunningham ME, Fouladian JE, Chen M, Spiegel BMR. Public Perceptions Regarding Use of Virtual Reality in Health Care: A Social Media Content Analysis Using Facebook. J Med Internet Res 2017;19(12):e419. PMID: 29258975
142. Kendra RL, Karki S, Eickholt JL, Gandy L. Characterizing the Discussion of Antibiotics in the Twittersphere: What is the Bigger Picture?. J Med Internet Res 2015;17(6):e154. PMID: 26091775
143. Khan M.A.H., Iwai M., Sezaki K. A robust and scalable framework for detecting self-reported illness from twitter 2012 IEEE 14th International Conference on e-Health Networking, Applications and Services, Healthcom 2012, 6379425:303-308 DOI: 10.1109/HealthCom.2012.6379425
144. Khoury MJ, Gwinn M, Dotson WD, Schully SD. Knowledge integration at the center of genomic medicine. Genet Med. 2012 Jul;14(7):643-7. PMID:22555656
145. Kim AE, Hopper T, Simpson S, Nonnemaker J, Lieberman AJ, Hansen H, Guillory J, Porter L. Using Twitter Data to Gain Insights into E-cigarette Marketing and Locations of Use: An Infoveillance Study. J Med Internet Res. 2015 Nov 6;17(11):e251. PMID:26545927
146. Kim M, Jung Y, Jung D, Hur C. Investigating the congruence of crowdsourced information with official government data: the case of pediatric clinics. J Med Internet Res. 2014 Feb 3;16(2):e29. PMID:24496094
147. Kim SJ, Marsch LA, Hancock JT, Das AK. Scaling Up Research on Drug Abuse and Addiction Through Social Media Big Data. J Med Internet Res. 2017 Oct 31;19(10):e353. PMID:29089287

148. Kim Y, Huang J, Emery S. Garbage in, Garbage Out: Data Collection, Quality Assessment and Reporting Standards for Social Media Data Use in Health Research, Infodemiology and Digital Disease Detection. J Med Internet Res. 2016 Feb 26;18(2):e41. PMID:26920122
149. Kim A, Miano T, Chew R, Eggers M, Nonnemaker J. Classification of Twitter Users Who Tweet About E-Cigarettes. JMIR Public Health Surveill 2017;3(3):e63. PMID: 28951381
150. Klembczyk JJ, Jalalpour M, Levin S, Washington RE, Pines JM, Rothman RE, Dugas AF. Google Flu Trends Spatial Variability Validated Against Emergency Department Influenza-Related Visits. J Med Internet Res. 2016 Jun 28;18(6):e175. PMID:27354313
151. Koh S, Gordon AS, Wienberg C, Sood SO, Morley S, Burke DM. Stroke Experiences in Weblogs: A Feasibility Study of Sex Differences. J Med Internet Res 2014;16(3):e84. PMID: 24647327
152. Konheim-Kalkstein YL, Miron-Shatz T, Israel LJ. How Women Evaluate Birth Challenges: Analysis of Web-Based Birth Stories. JMIR Pediatr Parent 2018;1(2):e12206. DOI: 10.2196/12206
153. Koschack J, Weibezahl L, Friede T, Himmel W, Makedonski P, Grabowski J. Scientific Versus Experiential Evidence: Discourse Analysis of the Chronic Cerebrospinal Venous Insufficiency Debate in a Multiple Sclerosis Forum. J Med Internet Res 2015;17(7):e159. PMID: 26133525
154. Kostkova P, Fowler D, Wiseman S, Weinberg JR. Major Infection Events Over 5 Years: How Is Media Coverage Influencing Online Information Needs of Health Care Professionals and the Public?. J Med Internet Res 2013;15(7):e107. PMID: 23856364
155. Krueger EA, Young SD. Twitter: A Novel Tool for Studying the Health and Social Needs of Transgender Communities. JMIR Mental Health. 2015;2(2).e16
156. Kürzinger ML, Schück S, Texier N, Abdellaoui R, Faviez C, Pouget J, Zhang L, Tcherny-Lessenot S, Lin S, Juhaeri J. Web-Based Signal Detection Using Medical Forums Data in France: Comparative Analysis. J Med Internet Res 2018;20(11):e10466. PMID: 30459145
157. Lachmar EM, Wittenborn AK, Bogen KW, McCauley HL. #MyDepressionLooksLike: Examining Public Discourse About Depression on Twitter. JMIR Ment Health 2017;4(4):e43. PMID: 29046270
158. Lama Y, Chen T, Dredze M, Jamison A, Quinn SC, Broniatowski DA. Discordance Between Human Papillomavirus Twitter Images and Disparities in Human Papillomavirus Risk and Disease in the United States: Mixed-Methods Analysis. J Med Internet Res 2018;20(9):e10244. PMID: 30217792
159. Lardon J, Abdellaoui R, Bellet F, Asfari H, Souvignet J, Texier N, Jaulent MC, Beyens MN, Burgun A, Bousquet C. Adverse Drug Reaction Identification and Extraction in Social Media: A Scoping Review. J Med Internet Res 2015;17(7):e171. PMID: 26163365
160. Lau AY, Siek KA, Fernandez-Luque L, Tange H, Chhanabhai P, Li SY, Elkin PL, Arjabi A, Walczowski L, Ang CS, Eysenbach G. The role of social media for patients and consumer health. Contribution of the IMIA Consumer Health Informatics Working Group. Yearb Med Inform. 2011;6:131-8. Review. PMID:21938338
161. Lavorgna L, Brigo F, Moccia M, Leocani L, Lanzillo R, Clerico M, Abbadessa G, Schmierer K, Solaro C, Prosperini L, Tedeschi G, Giovannoni G, Bonavita S. e-Health and multiple sclerosis: An update. Mult Scler. 2018 Nov;24(13):1657-1664. PMID:30231004
162. Lazard AJ, Saffer AJ, Wilcox GB, Chung AD, Mackert MS, Bernhardt JM. E-Cigarette Social Media Messages: A Text Mining Analysis of Marketing and Consumer Conversations on Twitter. JMIR Public Health Surveill 2016;2(2):e171. PMID: 27956376
163. Leal Neto O, Dimech GS, Libel M, de Souza WV, Cesse E, Smolinski M, Oliveira W, Albuquerque J. Saúde na Copa: The World's First Application of Participatory Surveillance for a Mass Gathering at FIFA World Cup 2014, Brazil. JMIR Public Health Surveill 2017;3(2):e26. PMID: 28473308
164. Lee JL, DeCamp M, Dredze M, Chisolm MS, Berger ZD. What Are Health-Related Users Tweeting? A Qualitative Content Analysis of Health-Related Users and Their Messages on Twitter. J Med Internet Res 2014;16(10):e237. PMID: 25591063
165. Lee D, Lee H, Choi M. Examining the Relationship Between Past Orientation and US Suicide Rates: An Analysis Using Big Data-Driven Google Search Queries. J Med Internet Res 2016;18(2):e35. PMID: 26868917

166. Lenoir P, Moulahi B, Azé J, Bringay S, Mercier G, Carbonnel F. Raising Awareness About Cervical Cancer Using Twitter: Content Analysis of the 2015 #SmearForSmear Campaign. J Med Internet Res 2017;19(10):e344. PMID: 29038096
167. Leung R, Guo H, Pan X. Social Media Users' Perception of Telemedicine and mHealth in China: Exploratory Study. JMIR Mhealth Uhealth 2018;6(9):e181. PMID: 30274969
168. Li Q, Wang C, Liu R, Wang L, Zeng DD, Leischow SJ. Understanding Users' Vaping Experiences from Social Media: Initial Study Using Sentiment Opinion Summarization Techniques. J Med Internet Res. 2018 Aug 15;20(8):e252. PMID:30111530
169. Liang B, Scammon DL. Incidence of Online Health Information Search: A Useful Proxy for Public Health Risk Perception. J Med Internet Res 2013;15(6):e114. PMID: 23773974
170. Lienemann BA, Unger JB, Cruz TB, Chu KH. Methods for Coding Tobacco-Related Twitter Data: A Systematic Review. J Med Internet Res 2017;19(3):e91. PMID: 28363883
171. Ling R, Lee J. Disease Monitoring and Health Campaign Evaluation Using Google Search Activities for HIV and AIDS, Stroke, Colorectal Cancer, and Marijuana Use in Canada: A Retrospective Observational Study. JMIR Public Health Surveill. 2016 Oct 12;2(2):e156. PMID:27733330
172. Liu X, Bao Z, Liu H, Wang Z. The quality and characteristics of leading general hospitals' websites in China. J Med Syst. 2011 Dec;35(6):1553-62. PMID:20703762
173. Liu Y, Mei Q, Hanauer DA, Zheng K, Lee JM. Use of Social Media in the Diabetes Community: An Exploratory Analysis of Diabetes-Related Tweets. JMIR Diabetes 2016;1(2):e4. PMID: 30291053
174. Liu K, Huang S, Miao ZP, Chen B, Jiang T, Cai G, Jiang Z, Chen Y, Wang Z, Gu H, Chai C, Jiang J. Identifying Potential Norovirus Epidemics in China via Internet Surveillance. J Med Internet Res 2017;19(8):e282. PMID: 28790023
175. Liu S, Zhu M, Yu DJ, Rasin A, Young SD. Using Real-Time Social Media Technologies to Monitor Levels of Perceived Stress and Emotional State in College Students: A Web-Based Questionnaire Study. JMIR Ment Health 2017;4(1):e2. PMID: 28073737
176. Liu S, Zhu M, Young SD. Monitoring Freshman College Experience Through Content Analysis of Tweets: Observational Study. JMIR Public Health Surveill 2018;4(1):e5. PMID: 29326096
177. Livelo E.D., Cheng C. Intelligent dengue infoveillance using gated recurrent neural learning and cross-label frequencies Proceedings - 2018 IEEE International Conference on Agents, ICA 2018, 8459963:2-7 DOI: 10.1109/AGENTS.2018.8459963
178. Lu FS, Hou S, Baltrusaitis K, Shah M, Leskovec J, Sosic R, Hawkins J, Brownstein J, Conidi G, Gunn J, Gray J, Zink A, Santillana M. Accurate Influenza Monitoring and Forecasting Using Novel Internet Data Streams: A Case Study in the Boston Metropolis. JMIR Public Health Surveill 2018;4(1):e4.PMID: 29317382
179. Lyles CR, Godbehere A, Le G, El Ghaoui L, Sarkar U. Applying Sparse Machine Learning Methods to Twitter: Analysis of the 2012 Change in Pap Smear Guidelines. A Sequential Mixed-Methods Study. JMIR Public Health Surveill 2016;2(1):e21. PMID: 27288093
180. Mackey TK, Liang BA. Global Reach of Direct-to-Consumer Advertising Using Social Media for Illicit Online Drug Sales. J Med Internet Res 2013;15(5):e105. DOI: 10.2196/jmir.2610. PMID: 23718965
181. Mackey T, Kalyanam J, Klugman J, Kuzmenko E, Gupta R. Solution to Detect, Classify, and Report Illicit Online Marketing and Sales of Controlled Substances via Twitter: Using Machine Learning and Web Forensics to Combat Digital Opioid Access. J Med Internet Res 2018;20(4):e10029. PMID: 29613851
182. Madden KM. The Seasonal Periodicity of Healthy Contemplations About Exercise and Weight Loss: Ecological Correlational Study. JMIR Public Health Surveill 2017;3(4):e92.PMID: 29237582
183. Mahoney LM, Tang T, Ji K, Ulrich-Schad J. The Digital Distribution of Public Health News Surrounding the Human Papillomavirus Vaccination: A Longitudinal Infodemiology Study. JMIR Public Health Surveill. 2015 Mar 18;1(1):e2. PMID:27227125
184. Mahroum N, Watad A, Rosselli R, Brigo F, Chiesa V, Siri A, Ben-Ami Shor D, Martini M, Bragazzi NL, Adawi M. An infodemiological investigation of the so-called "Fluad effect" during the 2014/2015 influenza

vaccination campaign in Italy: Ethical and historical implications. Hum Vaccin Immunother. 2018 Mar 4;14(3):712-718. PMID:29293392

185. Majumder MS, Santillana M, Mekaru SR, McGinnis DP, Khan K, Brownstein JS. Utilizing Nontraditional Data Sources for Near Real-Time Estimation of Transmission Dynamics During the 2015-2016 Colombian Zika Virus Disease Outbreak. JMIR Public Health Surveill 2016;2(1):e30. PMID: 27251981

186. Manchaiah V, Ratinaud P, Andersson G. Representation of Tinnitus in the US Newspaper Media and in Facebook Pages: Cross-Sectional Analysis of Secondary Data. Interact J Med Res 2018;7(1):e9. PMID: 29739734

187. Mao C, Wu XY, Fu XH, Di MY, Yu YY, Yuan JQ, Yang ZY, Tang JL. An Internet-Based Epidemiological Investigation of the Outbreak of H7N9 Avian Influenza A in China Since Early 2013. J Med Internet Res 2014;16(9):e221. PMID: 25257217

188. Marcon AR, Klostermann P, Caulfield T. Chiropractic and Spinal Manipulation Therapy on Twitter: Case Study Examining the Presence of Critiques and Debates. JMIR Public Health Surveill. 2016 Sep 16;2(2):e153. PMID:27637456

189. Marcus MA, Westra HA, Eastwood JD, Barnes KL, Mobilizing Minds Research Group . What Are Young Adults Saying About Mental Health? An Analysis of Internet Blogs. J Med Internet Res 2012;14(1):e17. PMID: 22569642

190. Martinez M, Park SB, Maison I, Mody V, Soh LS, Parihar HS. iOS Appstore-Based Phone Apps for Diabetes Management: Potential for Use in Medication Adherence. JMIR Diabetes 2017;2(2):e12. PMID: 30291096

191. Martinez-Arroyo G, Ramos-Gomez S, Rojero-Gil EK, Rojas-Gongora JA, Barajas-Ochoa A, Bustamante-Montes LP, YaÃ±ez J, Ramos-Remus C. Potential uses of an infodemiology approach for health-care services for rheumatology. Clin Rheumatol. 2019 Mar;38(3):869-876. PMID:30448932

192. Martinez-Millana A, Fernandez-Llatas C, Basagoiti Bilbao I, Traver Salcedo M, Traver Salcedo V. Evaluating the Social Media Performance of Hospitals in Spain: A Longitudinal and Comparative Study. J Med Internet Res 2017;19(5):e181. PMID: 28536091

193. Martins-Filho PRS, Mendes MLT, Reinheimer DM, do Nascimento-JÃºnior EM, Vaez AC, Santos VS, Santos HP Jr. Femicide trends in Brazil: relationship between public interest and mortality rates. Arch Womens Ment Health. 2018 Oct;21(5):579-582. PMID:29594384

194. Massey PM, Leader A, Yom-Tov E, Budenz A, Fisher K, Klassen AC. Applying Multiple Data Collection Tools to Quantify Human Papillomavirus Vaccine Communication on Twitter. J Med Internet Res 2016;18(12):e318. PMID: 27919863

195. Matsuda S, Aoki K, Tomizawa S, Sone M, Tanaka R, Kuriki H, Takahashi Y. Analysis of Patient Narratives in Disease Blogs on the Internet: An Exploratory Study of Social Pharmacovigilance. JMIR Public Health Surveill 2017;3(1):e10. PMID: 28235749

196. Mavragani A., Ochoa G. Forecasting AIDS prevalence in the United States using online search traffic data. J Big Data. 2018;5(1):17. DOI: 10.1186/s40537-018-0126-7

197. Mavragani A., Ochoa G. Infoveillance of infectious diseases in USA: STDs, tuberculosis, and hepatitis. J Big Data. 2018;5(1):30. DOI: 10.1186/s40537-018-0140-9

198. Mavragani A, Ochoa G, Tsagarakis KP. Assessing the Methods, Tools, and Statistical Approaches in Google Trends Research: Systematic Review. J Med Internet Res. 2018 Nov 6;20(11):e270. PMID:30401664

199. Mavragani A, Sampri A, Sypsa K, Tsagarakis KP. Integrating Smart Health in the US Health Care System: Infodemiology Study of Asthma Monitoring in the Google Era. JMIR Public Health Surveill. 2018 Mar 12;4(1):e24. PMID:29530839

200. Mazzocut M, Truccolo I, Antonini M, Rinaldi F, Omero P, Ferrarin E, De Paoli P, Tasso C. Web Conversations About Complementary and Alternative Medicines and Cancer: Content and Sentiment Analysis. J Med Internet Res 2016;18(6):e120. PMID: 27311444

201. McNaughton EC, Coplan PM, Black RA, Weber SE, Chilcoat HD, Butler SF. Monitoring of Internet Forums to Evaluate Reactions to the Introduction of Reformulated OxyContin to Deter Abuse. J Med Internet Res 2014;16(5):e119. PMID: 24800858

202. Meaney S, Cussen L, Greene RA, O'Donoghue K. Reaction on Twitter to a Cluster of Perinatal Deaths: A Mixed Method Study. JMIR Public Health Surveill. 2016 Jul 27;2(2):e36. PMID:27466002
203. Mejova Y, Weber I, Fernandez-Luque L. Online Health Monitoring using Facebook Advertisement Audience Estimates in the United States: Evaluation Study. JMIR Public Health Surveill. 2018 Mar 28;4(1):e30. PMID:29592849
204. McIver DJ, Hawkins JB, Chunara R, Chatterjee AK, Bhandari A, Fitzgerald TP, Jain SH, Brownstein JS. Characterizing Sleep Issues Using Twitter. J Med Internet Res 2015;17(6):e140. PMID: 26054530
205. Menachemi N, Rahurkar S, Rahurkar M. Using Web-Based Search Data to Study the Public's Reactions to Societal Events: The Case of the Sandy Hook Shooting. JMIR Public Health Surveill. 2017 Mar 23;3(1):e12. PMID:28336508 .
206. Mendiola MF, Kalnicki M, Lindenauer S. Valuable Features in Mobile Health Apps for Patients and Consumers: Content Analysis of Apps and User Ratings. JMIR Mhealth Uhealth 2015;3(2):e40. PMID: 25972309
207. Metwally O, Blumberg S, Ladabaum U, Sinha SR. Using Social Media to Characterize Public Sentiment Toward Medical Interventions Commonly Used for Cancer Screening: An Observational Study. J Med Internet Res 2017;19(6):e200. PMID: 28592395.
208. Miller M, Banerjee T, Muppalla R, Romine W, Sheth A. What Are People Tweeting About Zika? An Exploratory Study Concerning Its Symptoms, Treatment, Transmission, and Prevention. JMIR Public Health Surveill 2017;3(2):e38. PMID: 28630032
209. Mishori R, Singh LO, Levy B, Newport C. Mapping physician Twitter networks: describing how they work as a first step in understanding connectivity, information flow, and message diffusion. J Med Internet Res. 2014 Apr 14;16(4):e107. PMID:24733146
210. Mnadla S, Bragazzi NL, Rouissi M, Chaalali A, Siri A, Padulo J, ArdigÃ² LP, Brigo F, Chamari K, Knechtle B. Infodemiological data of Ironman Triathlon in the study period 2004-2013. Data Brief. 2016 Aug 27;9:123-7. PMID:27642618
211. Moccia M, Brigo F, Tedeschi G, Bonavita S, Lavorgna L. Neurology and the Internet: a review. Neurol Sci. 2018 Jun;39(6):981-987. PMID:29594831
212. Mollema L, Harmsen IA, Broekhuizen E, Clijnk R, De Melker H, Paulussen T, Kok G, Ruiter R, Das E. Disease Detection or Public Opinion Reflection? Content Analysis of Tweets, Other Social Media, and Online Newspapers During the Measles Outbreak in the Netherlands in 2013. J Med Internet Res 2015;17(5):e128.PMID: 26013683
213. Mowery D, Smith H, Cheney T, Stoddard G, Coppersmith G, Bryan C, Conway M. Understanding Depressive Symptoms and Psychosocial Stressors on Twitter: A Corpus-Based Study. J Med Internet Res 2017;19(2):e48. PMID: 28246066
214. Mukhija D, Venkatraman A, Nagpal SJS. Effectivity of Awareness Months in Increasing Internet Search Activity for Top Malignancies Among Women. JMIR Public Health Surveill. 2017 Aug 21;3(3):e55. PMID:28827213
215. Muralidhara S, Paul MJ. #Healthy Selfies: Exploration of Health Topics on Instagram. JMIR Public Health Surveill 2018;4(2):e10150. PMID: 29959106
216. Myslín M, Zhu SH, Chapman W, Conway M. Using Twitter to Examine Smoking Behavior and Perceptions of Emerging Tobacco Products. J Med Internet Res 2013;15(8):e174. PMID: 23989137
217. Nagar R, Yuan Q, Freifeld CC, Santillana M, Nojima A, Chunara R, Brownstein JS. A case study of the New York City 2012-2013 influenza season with daily geocoded Twitter data from temporal and spatiotemporal perspectives. J Med Internet Res. 2014 Oct 20;16(10):e236. PMID:25331122
218. Nagel AC, Tsou MH, Spitzberg BH, An L, Gawron JM, Gupta DK, Yang JA, Han S, Peddecord KM, Lindsay S, Sawyer MH. The complex relationship of realspace events and messages in cyberspace: case study of influenza and pertussis using tweets. J Med Internet Res. 2013 Oct 24;15(10):e237. PMID:24158773
219. Nakada H, Yuji K, Tsubokura M, Ohsawa Y, Kami M. Development of a national agreement on human papillomavirus vaccination in Japan: an infodemiology study. J Med Internet Res. 2014 May 15;16(5):e129. PMID:24834471

220. Nakhasi A, Shen AX, Passarella RJ, Appel LJ, Anderson CA. Online Social Networks That Connect Users to Physical Activity Partners: A Review and Descriptive Analysis. J Med Internet Res 2014;16(6):e153. PMID: 24936569

221. Nascimento TD, DosSantos MF, Danciu T, DeBoer M, van Holsbeeck H, Lucas SR, Aiello C, Khatib L, Bender MA; UMSoD (Under)Graduate Class Of 2014., Zubieta JK, DaSilva AF. Real-time sharing and expression of migraine headache suffering on Twitter: a cross-sectional infodemiology study. J Med Internet Res. 2014 Apr 3;16(4):e96. PMID:24698747

222. Nguyen QC, Li D, Meng HW, Kath S, Nsoesie E, Li F, Wen M. Building a National Neighborhood Dataset From Geotagged Twitter Data for Indicators of Happiness, Diet, and Physical Activity. JMIR Public Health Surveill 2016;2(2):e158. PMID: 27751984

223. Nishimoto N, Ota M, Yagahara A, Ogasawara K. Estimating the Duration of Public Concern After the Fukushima Dai-ichi Nuclear Power Station Accident From the Occurrence of Radiation Exposure-Related Terms on Twitter: A Retrospective Data Analysis. JMIR Public Health Surveill. 2016 Nov 25;2(2):e168. PMID:27888168

224. Noll-Hussong M. Whiplash Syndrome Reloaded: Digital Echoes of Whiplash Syndrome in the European Internet Search Engine Context. JMIR Public Health Surveill. 2017 Mar 27;3(1):e15. PMID:28347974

225. Nsoesie EO, Buckeridge DL, Brownstein JS. Guess Who's Not Coming to Dinner? Evaluating Online Restaurant Reservations for Disease Surveillance. J Med Internet Res 2014;16(1):e22. PMID: 24451921

226. Odlum M, Yoon S, Broadwell P, Brewer R, Kuang D. How Twitter Can Support the HIV/AIDS Response to Achieve the 2030 Eradication Goal: In-Depth Thematic Analysis of World AIDS Day Tweets. JMIR Public Health Surveill. 2018 Nov 22;4(4):e10262. PMID:30467102

227. Oldroyd RA, Morris MA, Birkin M. Identifying Methods for Monitoring Foodborne Illness: Review of Existing Public Health Surveillance Techniques. JMIR Public Health Surveill. 2018 Jun 6;4(2):e57. PMID:29875090

228. Oser TK, Oser SM, McGinley EL, Stuckey HL. A Novel Approach to Identifying Barriers and Facilitators in Raising a Child With Type 1 Diabetes: Qualitative Analysis of Caregiver Blogs. JMIR Diabetes 2017;2(2):e27. PMID: 30291073

229. Ozan-Rafferty ME, Johnson JA, Shah GH, Kursun A. In the Words of the Medical Tourist: An Analysis of Internet Narratives by Health Travelers to Turkey. J Med Internet Res 2014;16(2):e43. PMID: 24513565

230. Pan CL, Lin CH, Lin YR, Wen HY, Wen JC. The Significance of Witness Sensors for Mass Casualty Incidents and Epidemic Outbreaks. J Med Internet Res 2018;20(2):e39. PMID: 29396388

231. Park SH, Hong SH. Identification of Primary Medication Concerns Regarding Thyroid Hormone Replacement Therapy From Online Patient Medication Reviews: Text Mining of Social Network Data. J Med Internet Res 2018;20(10):e11085. PMID: 30355555

232. Peiper NC, Baumgartner PM, Chew RF, Hsieh YP, Bieler GS, Bobashev GV, Siege C, Zarkin GA. Patterns of Twitter Behavior Among Networks of Cannabis Dispensaries in California. J Med Internet Res 2017;19(7):e236. PMID: 28676471

233. Pervaiz F, Pervaiz M, Abdur Rehman N, Saif U. FluBreaks: Early Epidemic Detection from Google Flu Trends. J Med Internet Res 2012;14(5):e125. PMID: 23037553

234. Pesala S, Virtanen MJ, Sane J, Mustonen P, Kaila M, Helve O. Health Information-Seeking Patterns of the General Public and Indications for Disease Surveillance: Register-Based Study Using Lyme Disease. JMIR Public Health Surveill. 2017 Nov 6;3(4):e86. PMID:29109071

235. Pesala S, Virtanen MJ, Sane J, Jousimaa J, Lyytikäinen O, Murtopuro S, Mustonen P, Kaila M, Helve O. Health Care Professionals' Evidence-Based Medicine Internet Searches Closely Mimic the Known Seasonal Variation of Lyme Borreliosis: A Register-Based Study. JMIR Public Health Surveill 2017;3(2):e19. PMID: 28400357

236. Phillips CA, Barz Leahy A, Li Y, Schapira MM, Bailey LC, Merchant RM. Relationship Between State-Level Google Online Search Volume and Cancer Incidence in the United States: Retrospective Study. J Med Internet Res. 2018 Jan 8;20(1):e6. PMID:29311051

237. Poirier C, Lavenu A, Bertaud V, Campillo-Gimenez B, Chazard E, Cuggia M, BouzillÃ G. Real Time Influenza Monitoring Using Hospital Big Data in Combination with Machine Learning Methods: Comparison Study. JMIR Public Health Surveill. 2018 Dec 21;4(4):e11361. PMID:30578212
238. Pretorius KA, Mackert M, Wilcox GB. Sudden Infant Death Syndrome and Safe Sleep on Twitter: Analysis of Influences and Themes to Guide Health Promotion Efforts. JMIR Pediatr Parent 2018;1(2):e10435. DOI: 10.2196/10435
239. Priest C, Knopf A, Groves D, Carpenter JS, Furrey C, Krishnan A, Miller WR, Otte JL, Palakal M, Wiehe S, Wilson J. Finding the Patient's Voice Using Big Data: Analysis of Users' Health-Related Concerns in the ChaCha Question-and-Answer Service (2009-2012). J Med Internet Res. 2016 Mar 9;18(3):e44. PMID:26960745
240. Rabarison KM, Croston MA, Englar NK, Bish CL, Flynn SM, Johnson CC. Measuring Audience Engagement for Public Health Twitter Chats: Insights From #LiveFitNOLA. JMIR Public Health Surveill 2017;3(2):e34. PMID: 28596149
241. Radin M, Sciascia S. Infodemiology of systemic lupus erythematous using Google Trends. Lupus. 2017 Jul;26(8):886-889. PMID:28162030
242. Radzikowski J, Stefanidis A, Jacobsen KH, Croitoru A, Crooks A, Delamater PL. The Measles Vaccination Narrative in Twitter: A Quantitative Analysis. JMIR Public Health Surveill 2016;2(1):e1. PMID: 27227144
243. Rastegar-Mojarad M, Liu H, Nambisan P. Using Social Media Data to Identify Potential Candidates for Drug Repurposing: A Feasibility Study. JMIR Res Protoc 2016;5(2):e121. PMID: 27311964
244. Rastegar-Mojarad M, Ye Z, Wall D, Murali N, Lin S. Collecting and Analyzing Patient Experiences of Health Care From Social Media. JMIR Res Protoc 2015;4(3):e78. PMID: 26137885
245. Ricard BJ, Marsch LA, Crosier B, Hassanpour S. Exploring the Utility of Community-Generated Social Media Content for Detecting Depression: An Analytical Study on Instagram. J Med Internet Res 2018;20(12):e11817. PMID: 30522991
246. Risson V, Saini D, Bonzani I, Huisman A, Olson M. Patterns of Treatment Switching in Multiple Sclerosis Therapies in US Patients Active on Social Media: Application of Social Media Content Analysis to Health Outcomes Research. J Med Internet Res 2016;18(3):e62. PMID: 26987964
247. Roberts MJ, Perera M, Lawrentschuk N, Romanic D, Papa N, Bolton D. Globalization of Continuing Professional Development by Journal Clubs via Microblogging: A Systematic Review. J Med Internet Res 2015;17(4):e103. PMID: 25908092
248. Robillard JM, Whiteley L, Johnson TW, Lim J, Wasserman WW, Illes J. Utilizing Social Media to Study Information-Seeking and Ethical Issues in Gene Therapy. J Med Internet Res 2013;15(3):e44. PMID: 23470490
249. Roccetti M, Marfia G, Salomoni P, Prandi C, Zagari RM, Gningaye Kengni FL, Bazzoli F, Montagnani M. Attitudes of Crohn's Disease Patients: Infodemiology Case Study and Sentiment Analysis of Facebook and Twitter Posts. JMIR Public Health Surveill. 2017 Aug 9;3(3):e51. PMID:28793981
250. Rocheleau M, Sadasivam RS, Baquis K, Stahl H, Kinney RL, Pagoto SL, Houston TK. An Observational Study of Social and Emotional Support in Smoking Cessation Twitter Accounts: Content Analysis of Tweets. J Med Internet Res 2015;17(1):e18.PMID: 25589009
251. Rose SW, Jo CL, Binns S, Buenger M, Emery S, Ribisl KM. Perceptions of Menthol Cigarettes Among Twitter Users: Content and Sentiment Analysis. J Med Internet Res 2017;19(2):e56. PMID: 28242592
252. Rosenblum S, Yom-Tov E. Seeking Web-Based Information About Attention Deficit Hyperactivity Disorder: Where, What, and When. J Med Internet Res 2017;19(4):e126. PMID: 28432038
253. Sadah SA, Shahbazi M, Wiley MT, Hristidis V. A Study of the Demographics of Web-Based Health-Related Social Media Users. J Med Internet Res 2015;17(8):e194. PMID: 26250986
254. Sadah SA, Shahbazi M, Wiley MT, Hristidis V. Demographic-Based Content Analysis of Web-Based Health-Related Social Media. J Med Internet Res 2016;18(6):e148. PMID: 27296242
255. Saha K, Weber I, Birnbaum ML, De Choudhury M. Characterizing Awareness of Schizophrenia Among Facebook Users by Leveraging Facebook Advertisement Estimates. J Med Internet Res 2017;19(5):e156. PMID: 28483739

256. Samaras L, García-Barriocanal E, Sicilia MA. Syndromic Surveillance Models Using Web Data: The Case of Influenza in Greece and Italy Using Google Trends. JMIR Public Health Surveill 2017;3(4):e90. PMID: 29158208
257. Santos JC, Matos S. Analysing Twitter and web queries for flu trend prediction. Theor Biol Med Model. 2014 May 7;11 Suppl 1:S6. PMID:25077431.
258. Sanz-Lorente M, Wanden-Berghe C, Castejón-Bolea R, Sanz-Valero J. Web 2.0 Tools in the Prevention of Curable Sexually Transmitted Diseases: Scoping Review. J Med Internet Res 2018;20(3):e113. PMID: 29567633
259. Sarker A, Chandrashekar P, Magge A, Cai H, Klein A, Gonzalez G. Discovering Cohorts of Pregnant Women From Social Media for Safety Surveillance and Analysis. J Med Internet Res 2017;19(10):e361. PMID: 29084707
260. Sato A, Aramaki E, Shimamoto Y, Tanaka S, Kawakami K. Blog Posting After Lung Cancer Notification: Content Analysis of Blogs Written by Patients or Their Families. JMIR Cancer 2015;1(1):e5. PMID: 28410169
261. Schlichthorst M, King K, Turnure J, Sukunesan S, Phelps A, Pirkis J. Influencing the Conversation About Masculinity and Suicide: Evaluation of the Man Up Multimedia Campaign Using Twitter Data. JMIR Ment Health 2018;5(1):e14. PMID: 29449203.
262. Sciascia S, Radin M. What can Google and Wikipedia can tell us about a disease? Big Data trends analysis in Systemic Lupus Erythematosus. Int J Med Inform. 2017 Nov;107:65-69. PMID:29029693
263. Sciascia S, Radin M, Unlu O, Erkan D, Roccatello D. Infodemiology of antiphospholipid syndrome: Merging informatics and epidemiology. Eur J Rheumatol. 2018 Jul;5(2):92-95. PMID:30185355
264. Seabrook EM, Kern ML, Fulcher BD, Rickard NS. Predicting Depression From Language-Based Emotion Dynamics: Longitudinal Analysis of Facebook and Twitter Status Updates. J Med Internet Res 2018;20(5):e168. PMID: 29739736
265. Seidl S, Schuster B, Rüth M, Biedermann T, Zink A. What Do Germans Want to Know About Skin Cancer? A Nationwide Google Search Analysis From 2013 to 2017. J Med Internet Res 2018;20(5):e10327. PMID: 29698213
266. Sentana-Lledo D, Barbu CM, Ngo MN, Wu Y, Sethuraman K, Levy MZ. Seasons, Searches, and Intentions: What The Internet Can Tell Us About The Bed Bug (Hemiptera: Cimicidae) Epidemic. J Med Entomol. 2016 Jan;53(1):116-21. PMID:26474879
267. Seo DW, Jo MW, Sohn CH, Shin SY, Lee J, Yu M, Kim WY, Lim KS, Lee S. Cumulative Query Method for Influenza Surveillance Using Search Engine Data. J Med Internet Res 2014;16(12):e289. PMID: 25517353
268. Sewalk KC, Tuli G, Hswen Y, Brownstein JS, Hawkins JB. Using Twitter to Examine Web-Based Patient Experience Sentiments in the United States: Longitudinal Study. J Med Internet Res 2018;20(10):e10043. PMID: 30314959
269. SeyyedHosseini S., Asemi A., Shabani A., CheshmehSohrabi M. An infodemiology study on breast cancer in Iran: Health information supply versus health information demand in PubMed and Google Trends Electronic Library 2018;36(2):258-269 DOI: 10.1108/EL-03-2017-0062
270. SeyyedHosseini S., Asemi A., Shabani A., CheshmehSohrabi M. Infodemiology: A new presence concept in human information interaction based on eysenbach's view Iranian Journal of Information Processing Management 2017;32(3):605-629
271. SeyyedHosseini S., Asemi A., Shabani A., CheshmehSohrabi M. Scientific publication behavior versus information seeking behavior: An infodemiological study on stomach cancer. Webology 2017;14(1):21-31
272. Sharpe JD, Hopkins RS, Cook RL, Striley CW. Evaluating Google, Twitter, and Wikipedia as Tools for Influenza Surveillance Using Bayesian Change Point Analysis: A Comparative Analysis. JMIR Public Health Surveill 2016;2(2):e161. PMID: 27765731
273. Shi J, Salmon CT. Identifying Opinion Leaders to Promote Organ Donation on Social Media: Network Study. J Med Internet Res 2018;20(1):e7. PMID: 29317384

274. Simpson SS, Adams N, Brugman CM, Conners TJ. Detecting Novel and Emerging Drug Terms Using Natural Language Processing: A Social Media Corpus Study. JMIR Public Health Surveill 2018;4(1):e2. PMID: 29311050
275. Sinha MS, Freifeld CC, Brownstein JS, Donneyong MM, Rausch P, Lappin BM, Zhou EH, Dal Pan GJ, Pawar AM, Hwang TJ, Avorn J, Kesselheim AS. Social Media Impact of the Food and Drug Administration's Drug Safety Communication Messaging About Zolpidem: Mixed-Methods Analysis. JMIR Public Health Surveill 2018;4(1):e1. PMID: 29305342
276. Sinnenberg L, Mancheno C, Barg FK, Asch DA, Rivard CL, Horst-Martz E, Buttenheim A, Ungar L, Merchant R. Content Analysis of Metaphors About Hypertension and Diabetes on Twitter: Exploratory Mixed-Methods Study. JMIR Diabetes 2018;3(4):e11177. PMID: 30578222
277. Smith RJ, Crutchley P, Schwartz HA, Ungar L, Shofer F, Padrez KA, Merchant RM. Variations in Facebook Posting Patterns Across Validated Patient Health Conditions: A Prospective Cohort Study. J Med Internet Res 2017;19(1):e7. PMID: 28062392
278. Spyropoulos AC, Myrka A, Triller DM, Ragan S, York C, King JM, Lee TK. Uptake and Utilization of the Management of Anticoagulation in the Periprocedural Period App: Longitudinal Analysis. JMIR Mhealth Uhealth 2018;6(12):e11090. PMID: 30578235
279. Staal YC, van de Nobelen S, Havermans A, Talhout R. New Tobacco and Tobacco-Related Products: Early Detection of Product Development, Marketing Strategies, and Consumer Interest. JMIR Public Health Surveill. 2018 May 28;4(2):e55. PMID:29807884 .
280. Stefanidis A, Vraga E, Lamprianidis G, Radzikowski J, Delamater PL, Jacobsen KH, Pfoser D, Croitoru A, Crooks A. Zika in Twitter: Temporal Variations of Locations, Actors, and Concepts. JMIR Public Health Surveill 2017;3(2):e22. PMID: 28428164
281. Sudau F, Friede T, Grabowski J, Koschack J, Makedonski P, Himmel W. Sources of Information and Behavioral Patterns in Online Health Forums: Observational Study. J Med Internet Res 2014;16(1):e10. PMID: 24425598
282. Sueki H. The association of suicide-related Twitter use with suicidal behaviour: a cross-sectional study of young internet users in Japan. J Affect Disord. 2015 Jan 1;170:155-60. PMID:25240843
283. Sugawara Y, Narimatsu H, Tsuya A, Tanaka A, Fukao A. Medical Institutions and Twitter: A Novel Tool for Public Communication in Japan. JMIR Public Health Surveill 2016;2(1):e19. PMID: 27227154
284. Sugawara Y, Tanimoto T, Miyagawa S, Murakami M, Tsuya A, Tanaka A, Kami M, Narimatsu H. Scientific Misconduct and Social Media: Role of Twitter in the Stimulus Triggered Acquisition of Pluripotency Cells Scandal. J Med Internet Res 2017;19(2):e57. PMID: 28246071
285. Surian D, Nguyen DQ, Kennedy G, Johnson M, Coiera E, Dunn AG. Characterizing Twitter Discussions About HPV Vaccines Using Topic Modeling and Community Detection. J Med Internet Res 2016;18(8):e232. PMID: 27573910
286. Tafti A, Badger J, LaRose E, Shirzadi E, Mahnke A, Mayer J, Ye Z, Page D, Peissig P. Adverse Drug Event Discovery Using Biomedical Literature: A Big Data Neural Network Adventure. JMIR Med Inform 2017;5(4):e51. PMID: 29222076
287. Tana JC, Kettunen J, Eirola E, Paakkonen H. Diurnal Variations of Depression-Related Health Information Seeking: Case Study in Finland Using Google Trends Data. JMIR Ment Health. 2018 May 23;5(2):e43. doi: 10.2196/mental.9152. PMID:29792291
288. Tangherlini TR, Roychowdhury V, Glenn B, Crespi CM, Bandari R, Wadia A, Falahi M, Ebrahimzadeh E, Bastani R. “Mommy Blogs” and the Vaccination Exemption Narrative: Results From A Machine-Learning Approach for Story Aggregation on Parenting Social Media Sites. JMIR Public Health Surveill 2016;2(2):e166. PMID: 27876690
289. Tapi Nzali MD, Bringay S, Lavergne C, Mollevi C, Opitz T. What Patients Can Tell Us: Topic Analysis for Social Media on Breast Cancer. JMIR Med Inform 2017;5(3):e23. PMID: 28760725

290. Thackeray R, Neiger BL, Burton SH, Thackeray CR. Analysis of the Purpose of State Health Departments' Tweets: Information Sharing, Engagement, and Action. J Med Internet Res 2013;15(11):e255. PMID: 24217361
291. Tighe PJ, Goldsmith RC, Gravenstein M, Bernard HR, Fillingim RB. The Painful Tweet: Text, Sentiment, and Community Structure Analyses of Tweets Pertaining to Pain. J Med Internet Res 2015;17(4):e84. PMID: 25843553
292. Timpka T, Spreco A, Dahlström Ö, Eriksson O, Gursky E, Ekberg J, Blomqvist E, Strömgren M, Karlsson D, Eriksson H, Nyce J, Hinkula J, Holm E. Performance of eHealth Data Sources in Local Influenza Surveillance: A 5-Year Open Cohort Study. J Med Internet Res 2014;16(4):e116. PMID: 24776527
293. Tinschert P, Jakob R, Barata F, Kramer JN, Kowatsch T. The Potential of Mobile Apps for Improving Asthma Self-Management: A Review of Publicly Available and Well-Adopted Asthma Apps. JMIR Mhealth Uhealth 2017;5(8):e113. PMID: 28768606
294. Tougas ME, Chambers CT, Corkum P, Robillard JM, Gruzd A, Howard V, Kampen A, Boerner KE, Hundert AS. Social Media Content About Children's Pain and Sleep: Content and Network Analysis. JMIR Pediatr Parent 2018;1(2):e11193. DOI: 10.2196/11193
295. Triemstra JD, Poeppelman RS, Arora VM. Correlations Between Hospitals' Social Media Presence and Reputation Score and Ranking: Cross-Sectional Analysis. J Med Internet Res 2018;20(11):e289. PMID: 30409768
296. Troullos E, Baird L, Jayawardena S. Common Cold Symptoms in Children: Results of an Internet-Based Surveillance Program. J Med Internet Res 2014;16(6):e144. PMID: 24945090
297. Tsuya A, Sugawara Y, Tanaka A, Narimatsu H. Do Cancer Patients Tweet? Examining the Twitter Use of Cancer Patients in Japan. J Med Internet Res 2014;16(5):e137. PMID: 24867458
298. Tufts C, Polsky D, Volpp KG, Groeneveld PW, Ungar L, Merchant RM, Pelullo AP. Characterizing Tweet Volume and Content About Common Health Conditions Across Pennsylvania: Retrospective Analysis. JMIR Public Health Surveill 2018;4(4):e10834. PMID: 30522989
299. Tyrawski J, DeAndrea DC. Pharmaceutical Companies and Their Drugs on Social Media: A Content Analysis of Drug Information on Popular Social Media Sites. J Med Internet Res 2015;17(6):e130. PMID: 26032738
300. Utengen A, Rouholiman D, Gamble JG, Grajales FJ III, Pradhan N, Staley AC, Bernstein L, Young SD, Clauson KA, Chu LF. Patient Participation at Health Care Conferences: Engaged Patients Increase Information Flow, Expand Propagation, and Deepen Engagement in the Conversation of Tweets Compared to Physicians or Researchers. J Med Internet Res 2017;19(8):e280. PMID: 28818821
301. van Lent LG, Sungur H, Kunneman FA, van de Velde B, Das E. Too Far to Care? Measuring Public Attention and Fear for Ebola Using Twitter. J Med Internet Res 2017;19(6):e193. PMID: 28611015
302. Vasconcellos-Silva PR, Carvalho DBF, Trajano V, de La Rocque LR, Sawada ACMB, Juvanhol LL. Using Google Trends Data to Study Public Interest in Breast Cancer Screening in Brazil: Why Not a Pink February? JMIR Public Health Surveill. 2017 Apr 6;3(2):e17. PMID:28385679
303. Vickey T, Breslin JG. Online Influence and Sentiment of Fitness Tweets: Analysis of Two Million Fitness Tweets. JMIR Public Health Surveill 2017;3(4):e82. PMID: 29089294
304. Wagner M, Lampos V, Yom-Tov E, Pebody R, Cox IJ. Estimating the Population Impact of a New Pediatric Influenza Vaccination Program in England Using Social Media Content. J Med Internet Res 2017;19(12):e416. PMID: 29269339
305. Wakamiya S, Kawai Y, Aramaki E. Twitter-Based Influenza Detection After Flu Peak via Tweets With Indirect Information: Text Mining Study. JMIR Public Health Surveill. 2018 Sep 25;4(3):e65. PMID:30274968
306. Wang HW, Chen DR. Economic Recession and Obesity-Related Internet Search Behavior in Taiwan: Analysis of Google Trends Data. JMIR Public Health Surveill. 2018 Apr 6;4(2):e37. PMID:29625958
307. Wang HW, Chen DR, Yu HW, Chen YM. Forecasting the Incidence of Dementia and Dementia-Related Outpatient Visits With Google Trends: Evidence From Taiwan. J Med Internet Res 2015;17(11):e264. PMID: 26586281

308. Weeg C, Schwartz HA, Hill S, Merchant RM, Arango C, Ungar L. Using Twitter to Measure Public Discussion of Diseases: A Case Study. JMIR Public Health Surveill 2015;1(1):e6. PMID: 26925459
309. Williams SA, Terras M, Warwick C. How Twitter Is Studied in the Medical Professions: A Classification of Twitter Papers Indexed in PubMed. Med 2 0 2013;2(2):e2. DOI: 10.2196/med20.2269
310. Winchester DE, Baxter D, Markham MJ, Beyth RJ. Quality of Social Media and Web-Based Information Regarding Inappropriate Nuclear Cardiac Stress Testing and the Choosing Wisely Campaign: A Cross-Sectional Study. Interact J Med Res 2017;6(1):e6. PMID: 28473305
311. Wittmeier K, Holland C, Hobbs-Murison K, Crawford E, Beauchamp C, Milne B, Morris M, Keijzer R. Analysis of a Parent-Initiated Social Media Campaign for Hirschsprung's Disease. J Med Internet Res 2014;16(12):e288. PMID: 25499427
312. Wong PWC, Fu KW, Yau RSP, Ma HHM, Law YW, Chang SS, Yip PSF. Accessing Suicide-Related Information on the Internet: A Retrospective Observational Study of Search Behavior. J Med Internet Res 2013;15(1):e3. PMID: 23305632
313. Wong CA, Sap M, Schwartz A, Town R, Baker T, Ungar L, Merchant RM. Twitter Sentiment Predicts Affordable Care Act Marketplace Enrollment. J Med Internet Res 2015;17(2):e51. PMID: 25707038
314. Wongkoblap A, Vadillo MA, Curcin V. Researching Mental Health Disorders in the Era of Social Media: Systematic Review. J Med Internet Res. 2017 Jun 29;19(6):e228. PMID:28663166
315. Woo H, Cho Y, Shim E, Lee JK, Lee CG, Kim SH. Estimating Influenza Outbreaks Using Both Search Engine Query Data and Social Media Data in South Korea. J Med Internet Res. 2016 Jul 4;18(7):e177. PMID:27377323
316. Wood LN, Jamnagerwalla J, Markowitz MA, Thum DJ, McCarty P, Medendorp AR, Raz S, Kim JH. Public Awareness of Uterine Power Morcellation Through US Food and Drug Administration Communications: Analysis of Google Trends Search Term Patterns. JMIR Public Health Surveill 2018;4(2):e47. PMID: 29699965
317. Xu W, Liu Y. mHealthApps: A Repository and Database of Mobile Health Apps. JMIR Mhealth Uhealth 2015;3(1):e28. PMID: 25786060
318. Xu X, Litchman ML, Gee PM, Whatcott W, Chacon L, Holmes J, Srinivasan SS. Predicting Prediabetes Through Facebook Postings: Protocol for a Mixed-Methods Study. JMIR Res Protoc. 2018 Dec 14;7(12):e10720. PMID:30552084
319. Xu S, Markson C, Costello KL, Xing CY, Demissie K, Llanos AA. Leveraging Social Media to Promote Public Health Knowledge: Example of Cancer Awareness via Twitter. JMIR Public Health Surveill 2016;2(1):e17. PMID: 27227152
320. Yagahara A, Hanai K, Hasegawa S, Ogasawara K. Relationships Among Tweets Related to Radiation: Visualization Using Co-Occurring Networks. JMIR Public Health Surveill 2018;4(1):e26. PMID: 29549069
321. Yang H, Li S, Sun L, Zhang X, Hou J, Wang Y. Effects of the Ambient Fine Particulate Matter on Public Awareness of Lung Cancer Risk in China: Evidence from the Internet-Based Big Data Platform. JMIR Public Health Surveill 2017;3(4):e64. PMID: 28974484
322. Yin Z, Fabbri D, Rosenbloom ST, Malin B. A Scalable Framework to Detect Personal Health Mentions on Twitter. J Med Internet Res. 2015 Jun 5;17(6):e138. PMID:26048075
323. Yom-Tov E, Gabrilovich E. Postmarket drug surveillance without trial costs: discovery of adverse drug reactions through large-scale analysis of web search queries. J Med Internet Res. 2013 Jun 18;15(6):e124. PMID:23778053
324. Yom-Tov E, White RW, Horvitz E. Seeking Insights About Cycling Mood Disorders via Anonymized Search Logs. J Med Internet Res 2014;16(2):e65. PMID: 24568936
325. Yom-Tov E, Borsa D, Hayward AC, McKendry RA, Cox IJ. Automatic Identification of Web-Based Risk Markers for Health Events. J Med Internet Res 2015;17(1):e29. PMID: 25626480
326. Yom-Tov E, Borsa D, Cox IJ, McKendry RA. Detecting disease outbreaks in mass gatherings using Internet data. J Med Internet Res. 2014 Jun 18;16(6):e154. PMID:24943128

327. Yom-Tov E, Lev-Ran S. Adverse Reactions Associated With Cannabis Consumption as Evident From Search Engine Queries. JMIR Public Health Surveill 2017;3(4):e77. PMID: 29074469
328. Young SD. Social Media as a New Vital Sign: Commentary. J Med Internet Res 2018;20(4):e161. PMID: 29712631
329. Zeraatkar K, Ahmadi M. Trends of infodemiology studies: a scoping review. Health Info Libr J. 2018 Jun;35(2):91-120. PMID:29729073
330. Zhan Y, Liu R, Li Q, Leischow SJ, Zeng DD. Identifying Topics for E-Cigarette User-Generated Contents: A Case Study From Multiple Social Media Platforms. J Med Internet Res. 2017 Jan 20;19(1):e24. PMID:28108428
331. Zhang Z, Zheng X, Zeng DD, Leischow SJ. Tracking Dabbing Using Search Query Surveillance: A Case Study in the United States. J Med Internet Res 2016;18(9):e252. PMID: 27637361
332. Zhang Y, Allem JP, Unger JB, Boley Cruz T. Automated Identification of Hookahs (Waterpipes) on Instagram: An Application in Feature Extraction Using Convolutional Neural Network and Support Vector Machine Classification. J Med Internet Res 2018;20(11):e10513. PMID: 30452385
333. Zhang MW, Ho CS, Fang P, Lu Y, Ho RC. Methodology of developing a smartphone application for crisis research and its clinical application. Technol Health Care. 2014;22(4):547-59. PMID:24898865
334. Zhang N, Campo S, Janz KF, Eckler P, Yang J, Snetselaar LG, Signorini A. Electronic word of mouth on twitter about physical activity in the United States: exploratory infodemiology study. J Med Internet Res. 2013 Nov 20;15(11):e261. PMID:24257325
335. Zhao M, Yang CC. Drug Repositioning to Accelerate Drug Development Using Social Media Data: Computational Study on Parkinson Disease. J Med Internet Res 2018;20(10):e271. PMID: 30309833
336. Zheluk A, Gillespie JA, Quinn C. Searching for Truth: Internet Search Patterns as a Method of Investigating Online Responses to a Russian Illicit Drug Policy Debate. J Med Internet Res 2012;14(6):e165. PMID: 23238600
337. Zheluk A, Quinn C, Hercz D, Gillespie JA. Internet search patterns of human immunodeficiency virus and the digital divide in the Russian Federation: infoveillance study. J Med Internet Res. 2013 Nov 12;15(11):e256. doi: 10.2196/jmir.2936.
338. Zheluk A, Quinn C, Meylakhs P. Internet search and krokodil in the Russian Federation: an infoveillance study. J Med Internet Res. 2014 Sep 18;16(9):e212. PMID:25236385
