# Supplementary material for: Infodemiology and Infoveillance: Scoping Review
Source: J Med Internet Res. 2020 Apr 28;22(4):e16206. doi: 10.2196/16206 (PMC7189791; doi:10.2196/16206)

Multimedia Appendix 2:
Infodemiology and Infoveillance: A Scoping Review

Table A1 consists of the topics and subtopics (#publications) in infodemiology and infoveillance (2009-2018), while Figure 1 consists of the respective subtopics' pie charts.

Table A1. Topics and subtopics in infodemiology and infoveillance (2009-2018).

Category	(%)	Topics	#
**Drugs**	10.36%	Drugs (general)	(7)
		Drug Abuse/Misuse	(6)
		Cannabis/Marijuana	(5)
		Adverse Drug Reactions	(5)
		Illicit Drugs	(5)
		Pharmaceutical Drugs	(3)
		Dabbing	(1)
		Antibiotics	(1)
		Psychoactive Agents	(1)
		Reformulated OxyContin	(1)
**Conditions Diseases**	17.16%	Diabetes	(8)
		Multiple Sclerosis	(6)
		Conditions/Diseases/Health general	(6)
		Epilepsy	(5)
		Lupus	(2)
		Asthma	(2)
		Lyme Disease	(2)
		Neurology	(2)
		Dementia	(2)
		Stroke	(2)
		Pertussis	(2)
		Various[1]	(19)
**Mental Health**	8.28%	Depression	(8)
		Suicide	(7)
		Stress	(3)
		Mental Health general	(3)
		Schizophrenia	(2)
		Sleep issues	(1)
		Non-suicidal self-injury (NSSI)	(1)
		Mental disorders	(1)
		psychogenic nonepileptic seizures	(1)
		Cycling Mood Disorders	(1)
**Infectious Diseases**	7.99%	HIV/AIDS	(8)
		HPV	(6)
		STDs	(5)
		Syphilis	(2)
		Tuberculosis,	(2)
		Staphylococcus aureus (MRSA),	(1)
		Herpes Zoster Vaccination	(1)
		Infectious diseases	(1)
		Meningitis	(1)
**Smoking Alcohol**	8.58%	Smoking/Tobacco	(11)
		electronic cigarette/vaping	(10)
		Hookah	(4)
		Cigarettes/Cigars	(2)
		alcohol	(2)

Category	(%)	Topics	#
**Cancer**	6.80%	Cancer (general)	(6)
		Breast Cancer	(5)
		Skin Cancer	(2)
		Lung Cancer	(2)
		Malignancies	(2)
		Oncology	(1)
		Cervical Cancer	(1)
		Colorectal Cancer	(1)
		Stomach cancer	(1)
		Gynecologic cancer	(1)
		Alternative Medicines and Cancer	(1)
**Epidemics Outbreaks**	15.68%	Influenza	(27)
		Zika	(5)
		Measles	(4)
		dengue	(3)
		H1N1	(2)
		H7N9	(2)
		Norovirus	(2)
		Mayaro virus	(1)
		Ebola	(1)
		West-Nile Virus	(1)
		Rubella	(1)
		Cholera	(1)
		Acute Diarrhea	(1)
		Mass Casualty Incidents and Epidemic	(1)
		Disease Outbreaks in Mass Gatherings	(1)
**Mother Child**	2.66%	Birth Stories	(1)
		Abortion	(1)
		"Mommy Blogs" and the Vaccination	(1)
		Perinatal Deaths	(1)
		Pregnant Women	(1)
		Sudden Infant Death Syndrome	(1)
		Children's Pain and Sleep	(1)
		Cochrane Child Health	(1)
		Common Cold Symptoms in Children	(1)
**Diet Fitness Obesity Lifestyle**	4.14%	Fitness/Exercise	(5)
		Physical Activity	(3)
		Diet/Nutrition	(3)
		Obesity/Weight Loss	(2)
		Lifestyle	(1)
**Health Care Hospitals/Patients**	11.54%	Performance/Evaluation/Rating	(21)
		Dissemination/Integration	(18)
**Miscellaneous**	9.76%	Radiation	(3)
		Obamacare	(2)
		Ethics	(2)
		(STAP) Cell Case	(2)
		Various[2]	(24)

[1]Autism spectrum disorders; antiphospholipid syndrome; Attention Deficit Hyperactivity Disorder; B12-related symptoms; Whiplash; Crohn's Disease; rheumatology; silicosis; Fibromyalgia; Heart Failure Self-Care Management; Hirschsprung's Disease; Hypertension; Inflammatory Bowel Disease; Lynch Syndrome; Migraine-Headache Suffering; movement disorders; Thyroid Hormone; Tinnitus; Parkinson

[2]Anticoagulation in the Periprocedural Period; workplace compassion; Foodborne Illness; Ironman Triathlon; New York Storm; Sandy Hook Shooting; Bed bug; Nuclear Cardiac Stress Testing; Chiropractic and Spinal Manipulation Therapy ; Freshman College Experience; Organ Donation; Stem Cell Treatment; Transgender; sex-seeking MSM; Uterine Power Morcellation; Change in Pap Smear Guidelines; Telemedicine; mHealth; Virtual Reality in Health Care; health search behavior; Health Travelers; menopause; femicide/mortality rates

Figure A1: Pie charts of the individual subtopics of the identified health categories.

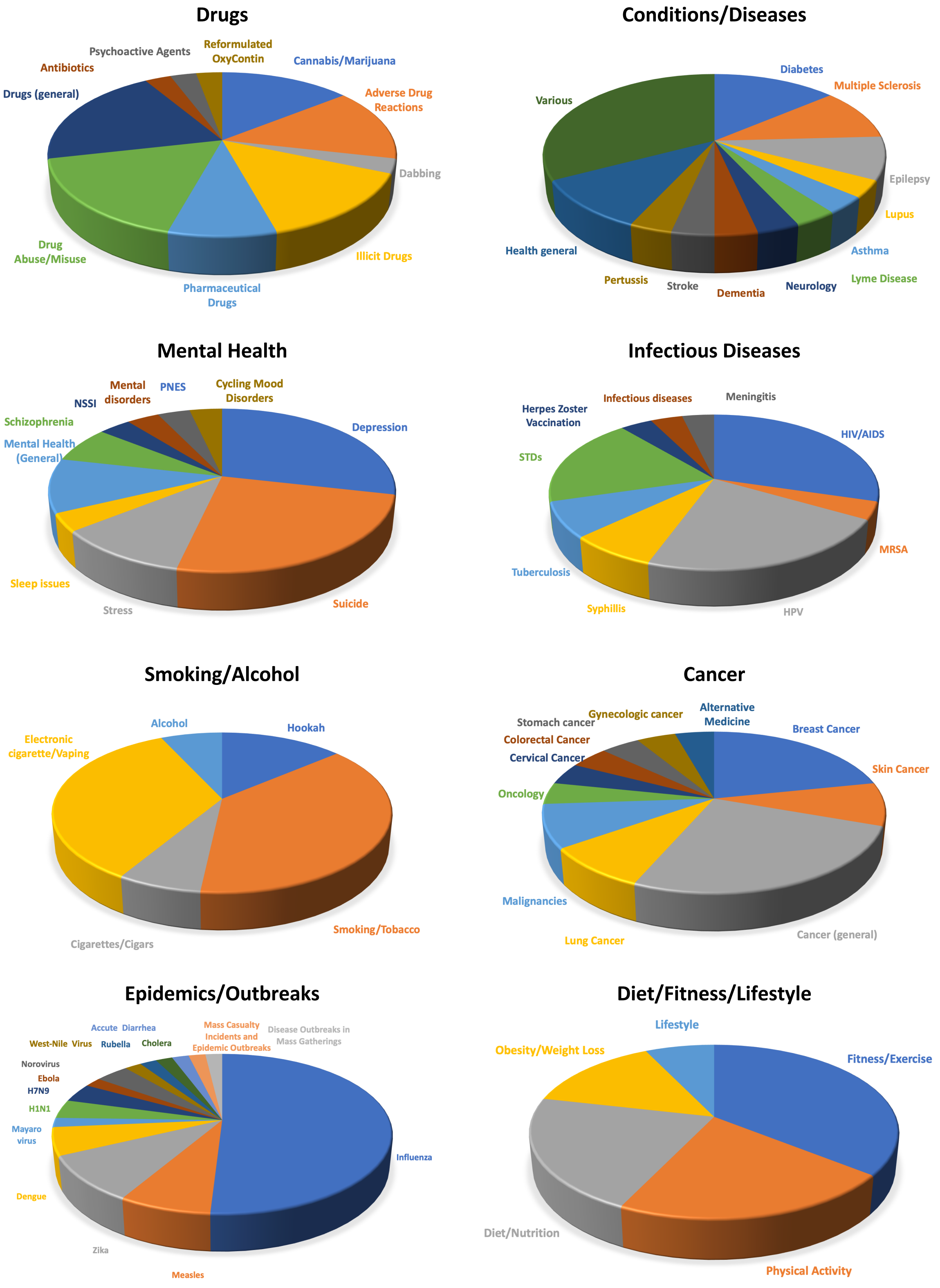

Supplement: Multimedia Appendix 2 [file jmir_v22i4e16206_app2.pdf]
